# Comprehensive and deep evaluation of structural variation detection pipelines with third-generation sequencing data

**DOI:** 10.1186/s13059-024-03324-5

**Published:** 2024-07-15

**Authors:** Zhi Liu, Zhi Xie, Miaoxin Li

**Affiliations:** 1 Program in Bioinformatics, Zhongshan School of Medicine, The Fifth Affiliated Hospital, Sun Yat-Sen University, Guangzhou, China; 2 Key Laboratory of Tropical Disease Control (Sun Yat-Sen University), Ministry of Education, Guangzhou, China; 3 State Key Laboratory of Ophthalmology, Zhongshan Ophthalmic Center, Sun Yat-Sen University, Guangzhou, China; 4 Center for Precision Medicine, Sun Yat-Sen University, Guangzhou, China; 5 Department of Psychiatry, The University of Hong Kong, Hong Kong, SAR China; 6 Guangdong Provincial Key Laboratory of Biomedical Imaging and Guangdong Provincial Engineering Research Center of Molecular Imaging, The Fifth Affiliated Hospital, Sun Yat-Sen University, Zhuhai, China

**Keywords:** Structural variation, Long-reads, Third-generation sequencing, Sequence aligner, SV caller, Pipeline evaluation

## Abstract

**Background:**

Structural variation (SV) detection methods using third-generation sequencing data are widely employed, yet accurately detecting SVs remains challenging. Different methods often yield inconsistent results for certain SV types, complicating tool selection and revealing biases in detection.

**Results:**

This study comprehensively evaluates 53 SV detection pipelines using simulated and real data from PacBio (CLR: Continuous Long Read, CCS: Circular Consensus Sequencing) and Nanopore (ONT) platforms. We assess their performance in detecting various sizes and types of SVs, breakpoint biases, and genotyping accuracy with various sequencing depths. Notably, pipelines such as Minimap2-cuteSV2, NGMLR-SVIM, PBMM2-pbsv, Winnowmap-Sniffles2, and Winnowmap-SVision exhibit comparatively higher recall and precision. Our findings also show that combining multiple pipelines with the same aligner, like pbmm2 or winnowmap, can significantly enhance performance. The individual pipelines’ detailed ranking and performance metrics can be viewed in a dynamic table: http://pmglab.top/SVPipelinesRanking.

**Conclusions:**

This study comprehensively characterizes the strengths and weaknesses of numerous pipelines, providing valuable insights that can improve SV detection in third-generation sequencing data and inform SV annotation and function prediction.

**Supplementary Information:**

The online version contains supplementary material available at 10.1186/s13059-024-03324-5.

## Background

Structural variations (SVs) refer to the variation length exceeding 50 bp in the genome belonging to a broad category of genomic variations [1–4]. The types of SV usually include DEL (deletion), INS (insertion), INV (inversion), DUP (duplication), TRA (translocation), and complex SVs. SVs contribute to the genetic diversity of human genomes, potentially influencing genes or regulatory regions, thus leading to phenotypic variation or susceptibility to diseases [3, 5, 6]. However, precisely detecting SVs is much more complex than detecting single-nucleotide variants due to their structural complexity and variable lengths [7–9]. The second-generation sequencing (SGS) technology, widely employed for sequencing purposes, often encounters difficulties in accurately identifying SVs owing to its limited read length [2, 8–10]. The emergence of third-generation sequencing (TGS), characterized by its ability to generate long reads, holds promise for more accurate SV detection [7–12].

Multiple methods and tools have been developed to detect SVs based on TGS. Most tools are built on the alignment strategy for SV detection, owing to lower resource consumption and higher speed than other strategies, such as genome-wide de novo assembly [11–14]. Under this strategy, an SV detection pipeline typically includes an aligner and a caller. There are five commonly used aligners to align long reads (including LRA [15], minimap2 [16, 17], NGMLR [13], pbmm2 (https://github.com/PacificBiosciences/pbmm2), and winnowmap [18, 19]). Ren and Chaisson developed LRA, which utilizes SDP with a concave-cost gap penalty, demonstrating improved sensitivity and specificity for SVs larger than 1 kb [15]. Minimap2 employs the seed-chain-align strategy to enhance alignment speed and incorporates heuristic methods to improve the accuracy of alignments [16]. NGMLR breaks down long reads into shorter fragments, aligns them to the genome, and then determines the optimal combination of these fragments, providing advantages in resolving SVs [13]. Pbmm2 and winnowmap are improvements to minimap2. Pbmm2 is specifically designed to handle PacBio data and achieve more accurate alignments. At the same time, winnowmap optimizes alignments of reads to repetitive regions in the genome [18, 19]. Meanwhile, caller tools are continuously being developed, for example, cuteSV [11], cuteSV2 [20], DeBreak [12], DELLY [14], and SVision [21]. Among them, cuteSV utilizes a clustering-and-refinement method to analyze signatures, enabling sensitive detection of SVs [11]. DeBreak detects large SVs using a local de novo assembly approach [12]. DELLY was initially designed for SGS and has been enhanced to detect SV in TGS data [14]. SVision utilizes an artificial neural network to enhance SV detection, particularly excelling in resolving complex SVs [22]. However, due to the complexity of SVs and noise in TGS data, tools based on various assumptions and models often exhibit varying performances and relatively low consistency in SV detection. Accurately detecting all SV sites and genotypes from TGS data remains a significant challenge for most existing tools [22, 23]. Therefore, a more thorough comparative analysis of these methods is required to effectively select aligners and callers in practical applications.

Despite previous evaluations bringing attention to SV pipeline calling, assessments based on TGS are still limited and lack comprehensiveness, indicating the need for further improvement and supplementation. For example, the evaluation of Zhou et al. provided interesting insights into the usage and performance of pipelines at an earlier stage. However, the aligners (e.g., GraphMap [24] and LAST [25]) they assessed are now less commonly used due to the evolution of tools and technologies [26]. Moreover, Bolognini and Magi’s pipeline evaluation [23] and the above studies did not consider the genotype accuracy regarding Mendelian error rate (MIER). Due to the lack of precise reference data, the MIER assessment may offer a favorable method to evaluate pipelines’ detection capabilities. Additionally, most studies overlooked the length and breakpoint deviation of SVs detected by pipelines, which is crucial for SV functionality annotation and prediction. While Kosugi et al. considered breakpoint and length deviation in their evaluation, the primary focus was on SGS calling algorithm performance rather than TGS [27]. Dierckxsens et al. developed Sim-it, a tool for simulating SVs and long reads, and evaluated the strengths and weaknesses of 7 callers and long-read sequencing platforms [28]. Their study introduced a new method called combiSV, which combines results from SV callers into a higher-quality call set with improved recall and precision. However, these evaluations mainly focused on callers and a few aligners, lacking analysis of the impact of aligners on SV detection. In addition to existing evaluation studies, available benchmark resources for SVs are gradually increasing, such as Genome in a Bottle (GIAB) [29], Human Genome Structural Variation Consortium (HGSV) [30], and The Human Pangenome Reference Consortium (HPRC) [31]. Although the studies and datasets mentioned above have provided many insights and assistance for better SV detection, a comprehensive evaluation of SV detection pipelines remains essential.

In this study, we evaluated the performance of 53 SV detection pipelines. These pipelines were established using five aligners and 12 callers. We used SVs collected from public databases as the SV benchmark for the evaluation datasets to simulate TGS data using Visor. For real data, the SV benchmarks and sequencing data were derived from HG002 (GIAB [29]), CHM13 (HPRC [30]), HG00096, HG00512, and NA12878 (HGSV [31]). Next, we investigated the performance of these pipelines in detecting various types of SVs in the samples. We explored different scenarios, focusing on 12 aspects, including length deviation, breakpoint accuracy, and Mendelian error rate (MIER) [32–34]. Finally, we discussed the performance improvements gained from merging multiple pipelines compared to using a single pipeline.

## Results

### Study design review

We initially assessed and compared the performance of 72 genomic SV detection pipelines. These pipelines were constructed by using six aligners (lordfast [35], LRA [15], minimap2 [16, 17], NGMLR [13], pbmm2 (https://github.com/PacificBiosciences/pbmm2), winnowmap [18, 19]) and 12 callers (cuteSV [11], cuteSV2 [20], DeBreak [12], DELLY [14], pbsv (https://github.com/PacificBiosciences/pbsv), Picky [36], NanoSV [37], NanoVar [22], Sniffles [13], Sniffles2 [34], SVIM [38], SVision [21]). These pipelines were executed within our laboratory server environment and tested against multiple benchmark samples (details in the “Methods” section). However, the output of pbmm2 lacks the “AS” tag required by the NanoVar caller in the BAM file. Additionally, lordfast led to too low accuracy in our testing datasets (Additional file 1: Fig. S1). Furthermore, we observed compatibility issues between certain callers (Sniffles, DELLY, Picky, NanoVar, NanoSV, and pbsv) and the LRA aligner’s BAM file. Consequently, we excluded pipelines such as LRA-Sniffles, LRA-DELLY, LRA-Picky, LRA-NanoVar, LRA-NanoSV, LRA-pbsv, pbmm2-NanoVar, and those associated with lordfast. Subsequently, we comprehensively analyzed and evaluated the remaining 53 pipelines (Additional file 2: Table S1). The accuracy, recall, and F1 score of these pipelines were assessed using Truvari (v2.1) [39] against high-quality SV benchmarks (see “Methods” and Supplementary Data). Performance comparison was based on the F1 measure, aggregating F1 scores across different SV types (DEL, INS, INV, DUP, BND) and precision and recall measures for each SV type, ranging from 0 to 5. Currently, many callers report translocations (TRA) in the form of breakpoints (BND), lacking complete TRA information. Therefore, referring to the work of Jiang et al. [11], we used the BND records in the VCF file, which might represent the TRA type. Our evaluation considered 12 critical factors: SV length deviation, breakpoint deviation, SV types, SV lengths, sequencing platform, sequencing depth, genotyping accuracy, Mendelian error rate, minimum supporting read number, reference genomes, computation consumption, and merging strategies.

The evaluation was conducted using benchmark SVs from both simulated and real datasets. The simulated dataset comprised 20,541 non-overlapping SVs, including 7214 deletions, 9989 insertions, and 51 inversions identified within the CHM1 sample call set (dbVAR database accession nstd137 [40], which includes INS sequence). Additionally, 2919 duplications and 368 translocations extracted from the KWS1 sample call set (dbVAR database accession nstd106 [41]) were included. The length distributions of deletions and insertions are similar, characterized by two main peaks at 50 bp and 300 bp. Duplication lengths are primarily concentrated in 50 bp and 3000 bp, while inversions exhibit peaks at 360 bp and 10 kb (Additional file 1: Fig. S2). All simulated translocation lengths are 10 kb [11]. The distribution of simulated SVs across the genome is generally consistent with that observed in real data (Additional file 1: Fig. S3). Due to the limited availability of real ground-truth SV datasets, pipelines were benchmarked only against insertion and deletion discovery in samples HG002 (GIAB [29]), CHM13 (HPRC [30]), HG00096, HG00512, and NA12878 (HGSV [31]). However, no benchmarking was conducted for duplications, inversions, or translocations for real samples [12]. As the SVs in HG002 are based on GRCh37, we utilized LiftOver to convert them to the GRCh38 version. Following conversion, we obtained 5418 deletions and 7266 insertions. For CHM13, there are 7622 deletions and 12,448 insertions. In HG00096, there are 6151 deletions and 10,091 insertions. HG00512 exhibits 6135 deletions and 10,118 insertions, while NA12878 displays 6115 deletions and 9956 insertions (Additional file 2: Table S2).

Reads utilized for SV detection pipelines were sourced from both simulated and real datasets. The VISOR (v1.1) [42] tool was employed to generate simulation reads (PacBio: CCS, CLR; Nanopore: R9.4, R10.4) using the human reference genome (version: GRCh38). Simulated reads from two platforms, PacBio and Nanopore, had mean read lengths of 8 kb, with default error models. Real read datasets for PacBio CCS included samples from CHM13, HG00096, HG002, HG003, HG004, HG00512, HG005, HG006, HG007, and NA12878, with average read lengths ranging from 10 to 18 kb (Additional file 2: Table S3). The CLR datasets encompassed samples from CHM13, HG002, HG003, HG004, HG00512, HG005, HG006, and HG007, with average read lengths ranging from 8 to 25 kb. Nanopore datasets included samples from CHM13, HG00096, HG002, HG003, HG004, HG00512, and NA12878, with average read lengths ranging from 8 to 56 kb (Additional file 2: Table S3). Additionally, real family data samples were utilized for assessment: Nanopore (HG002 (son), HG003 (father), HG004 (mother)), PacBio (HG002 (son), HG003, HG004), and (HG005 (son), HG006 (father), HG007 (mother)).

### Pipeline performance in simulated datasets

**Fig. 1:**
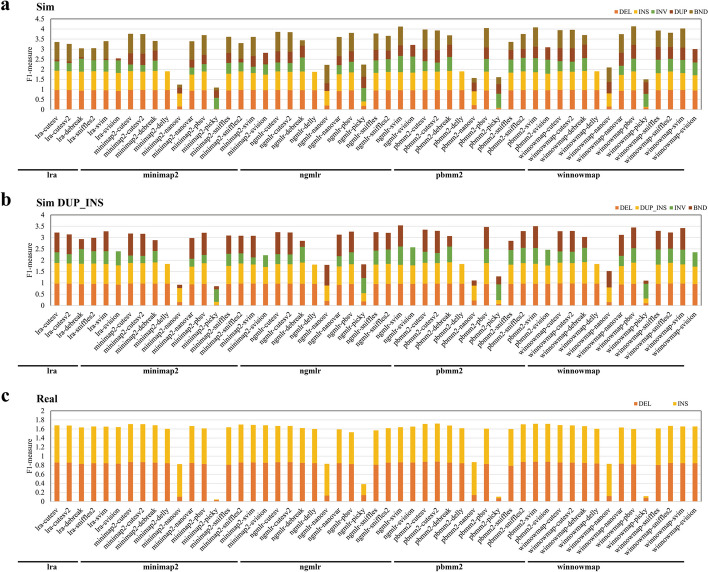
Performance of SV detection pipelines in different SV types (CCS). Precision and recall of DEL, DUP, INS, INV, and BND were determined with the simulated (**a**, **b** (DUP_INS)) and the real data (**c**). F1 measures, which combine precision and recall statistics (see the “Methods” section for details), are depicted for pipelines distinguished by different colored bars. Pipelines are categorized according to the alignment tools (lra, minimap2, ngmlr, pbmm2, winnowmap)

We first evaluated the performance of 53 pipelines with relatively high coverage of 25 × on the CCS platform, known for its higher sequencing accuracy according to previous studies [28, 43–46]. In the simulated data, 26 pipelines achieved an aggregated F1 measure exceeding 3.5, indicating superior performance (Fig. 1). Notable pipelines include winnowmap-pbsv, NGMLR-SVIM, pbmm2-SVIM, pbmm2-pbsv, and winnowmap-SVIM. However, SVision’s lack of TRA/BND reports leads to a lower aggregated F1 measure (< 3.5). The frequencies of different callers in the top 26 pipelines showed no large discrepancy. CuteSV, cuteSV2, pbsv, and SVIM each comprised 15.4%, followed by Sniffles and Sniffles2 at 11.5%, and DeBreak and NanoVar at 7.7%. In contrast, the aligners were dominated by four out of the five: winnowmap (30.8%), NGMLR (26.9%), pbmm2 (23.1%), and minimap2 (19.2%). Nevertheless, NGMLR-SVision, pbmm2-SVision, and winnowmap-SVision maintained high F1 measures (> 3) for the other four SV types (DEL, INS, DUP, and INV). Interestingly, the top-ranked pipelines varied by SV type. For DEL variant detection, winnowmap-cuteSV, minimap2-cuteSV, winnowmap-cuteSV2, winnowmap-Sniffles2, and minimap2-Sniffles2 exhibited top-tier performance (F1 > 0.97). For INS variant detection, LRA-cuteSV, minimap2-DeBreak, pbmm2-DeBreak, winnowmap-DeBreak, and LRA-DeBreak had higher F1 scores (> 0.94). For INV detection, the pipelines NGMLR-SVIM, NGMLR-SVision, pbmm2-Picky, pbmm2-SVIM, and pbmm2-SVision demonstrated superior performance (F1 > 0.69). Similarly, winnowmap-pbsv, winnowmap-DeBreak, winnowmap-SVision, winnowmap-cuteSV2, and winnowmap-cuteSV demonstrated superior performance in DUP detection (F1 > 0.65). Lastly, in BND detection, minimap2-pbsv, pbmm2-cuteSV2, minimap2-cuteSV2, minimap2-cuteSV, and pbmm2-cuteSV achieved outstanding F1 scores (F1 > 0.97).

The top-performing pipelines also exhibit variations based on precision and recall metrics. The top 5 pipelines for DEL variant detection with the highest precision include NGMLR-cuteSV, NGMLR-cuteSV2, winnowmap-cuteSV2, winnowmap-cuteSV, and LRA-cuteSV2 (precision > 0.99) (Additional file 1: Fig. S4, Additional file 4: Table S5). Regarding INS variant detection, the top 5 pipelines with the highest precision are LRA-cuteSV, pbmm2-pbsv, minimap2-pbsv, NGMLR-cuteSV, and LRA-cuteSV2 (precision > 0.98). Notably, cuteSV and cuteSV2 consistently demonstrate higher performance across F1-based prioritization as well. Regarding recall rate, the top 5 pipelines for DEL variant detection are LRA-SVision, winnowmap-SVision, minimap2-SVision, winnowmap-DeBreak, and NGMLR-SVision (recall > 0.96). For INS variant detection, the top 5 pipelines with the highest recall are LRA-SVision, pbmm2-DeBreak, pbmm2-SVision, minimap2-DeBreak, and winnowmap-DeBreak (recall > 0.94). SVision and DeBreak callers appear to exhibit higher recall rates than other callers.

Furthermore, we conducted an in-depth analysis of INS and DUP types using simulation, as some tools do not distinguish between them. Initially, we observed that a significant fraction of DUP events were incorrectly identified as INS events by callers (30 ~ 60%). In contrast, a minority of INS events were erroneously reported as DUP events by callers (0.153%) (Additional file 3: Table S4). Moreover, minimap2 was found to exacerbate the proportion of DUP events reported as INS by callers (~ 70%) compared to other aligners. Conversely, NGMLR increased the proportion of INS events reported as DUP by callers compared to other aligners (~ 10%). To address this discrepancy, we re-evaluated them using a merged DUP_INS type in simulated data (i.e., transforming all duplications and insertions into insertions for evaluation). After excluding the aligner LRA due to its poor performance, we observed a high consistency in the F1 measure levels of pipelines between DUP_INS and non-DUP_INS scenarios (Spearman correlation *R* = 0.82, Pearson correlation *R* = 0.9, *R*^2 = 0.95, Additional file 1: Fig. S5). Among the top 10 pipelines based on the F1 measure, eight were consistent for DUP_INS and non-DUP_INS scenarios (Fig. 1). This high consistency suggests that the relative performance of most pipelines may not be significantly affected by the misclassification of DUP and INS events.

Due to breakpoint deviations, length discrepancies, and the lack of INS sequences from some callers, most researchers do not consider sequence differences when evaluating pipeline performance on INS. Therefore, we initially assessed pipeline performance without considering INS sequence consistency, as shown in Additional file 1: Figs. S6–7. We then analyzed performance changes under varying levels of INS sequence consistency. Our findings indicated that in simulated data, pipelines exhibited smaller declines in F1 scores when INS sequence consistency ranged from 0.3 to 0.5 compared to 0.6 to 0.9 (Additional file 1: Fig. S7a). A similar performance decrease pattern was observed in real data. The decline in F1 scores was smaller when INS sequence consistency ranged from 0.3 to 0.5 compared to 0.6 to 0.9 (Additional file 1: Fig. S7b). This suggests that while most pipelines detect INS positions accurately, they often do not achieve high sequence consistency.

### Pipeline performance *in real* datasets

In real data, 26 pipelines exhibit F1 measures (for DEL and INS variant detection) exceeding 1.65 for the TGS data with coverage of 25 × produced by the CCS platform (Fig. 1). Notable performers include LRA-cuteSV, minimap2-DeBreak, pbmm2-DeBreak, winnowmap-DeBreak, and pbmm2-cuteSV. Within these pipelines, pbmm2-cuteSV2, pbmm2-SVIM, pbmm2-cuteSV, pbmm2-SVision, and pbmm2-Sniffles2 demonstrate top-tier performance for DEL variant detection (F1 > 0.87). Similarly, for INS variant detection, minimap2-cuteSV2, pbmm2-cuteSV2, minimap2-Sniffles2, minimap2-cuteSV, and pbmm2-SVision stand out (F1 > 0.83). The distribution of different callers among the 26 higher-performance pipelines includes cuteSV (19.2%), cuteSV2 (19.2%), Sniffles2 (15.4%), SVIM (15.4%), SVision (15.4%), DeBreak (11.5%), and NanoVar (3.8%). Regarding aligners, minimap2 (26.9%), pbmm2 (23.1%), winnowmap (23.1%), LRA (15.4%), and NGMLR (11.5%) are represented among these pipelines.

Regarding precision, the top five pipelines for DEL variant detection in real samples are pbmm2-cuteSV, NGMLR-cuteSV, minimap2-cuteSV, LRA-cuteSV, and LRA-cuteSV2 (precision > 0.91). For INS variant detection, NGMLR-Picky, minimap2-cuteSV, NGMLR-Sniffles, NGMLR-cuteSV, and pbmm2-cuteSV demonstrate precision exceeding 0.89 (Additional file 1: Fig. S4). Notably, although NGMLR-Picky has a lower F1 score, its precision remains high. Concerning recall, pbmm2-SVIM, pbmm2-SVision, minimap2-SVIM, minimap2-SVision, and pbmm2-Sniffles2 rank highest for DEL variants (recall > 0.85), while pbmm2-NanoSV, minimap2-SV, minimap2-SVision, pbmm2-SVision, and minimap2-SVIM excel for INS variant recall (recall > 0.85). Thus, the overall trends mirror those observed in simulated data, with cuteSV and cuteSV2 exhibiting higher precision and SVision showing a higher recall rate.

### Runtime and memory usage of aligners and callers of the pipelines

In large-scale tasks such as analyzing SV samples from populations, the computing resources utilized by the pipelines play a crucial role. We assessed the runtime and memory consumption of aligners and callers in the HG002 samples. Among the three sequencing technologies, minimap demonstrated the largest speed (CCS: 10 min; CLR, ONT: ~ 15 min) based on 5 × sequencing depth, while NGMLR was the slowest (CCS: 400 min, CLR: 80 min, ONT: 90 min, Additional file 1: Fig. S8a). Our findings align with the trend in Ren and Chaisson’s results [15], where the same aligner typically demonstrates a sequence of CLR > ONT > CCS regarding runtime across different data types. As expected, longer read lengths resulted in increased runtime for the aligner, with this influence being more pronounced for NGMLR (Additional file 1: Fig. S8a,e). In terms of memory consumption, LRA performed the best (CCS: 40 GB, CLR: 27 GB, ONT: 25 GB), while winnowmap consumed the most memory in CCS at 110 GB (Additional file 1: Fig. S8b). Among callers, Sniffles2 emerged as the fastest and the least memory-consuming compared to Sniffles, making it ideal for large-scale SV analysis. Additionally, cuteSV (1.6 min), cuteSV2 (1.5 min), and DeBreak (3 min) also performed well in terms of speed (Additional file 1: Fig. S8c). Among the callers, Sniffles2, SVIM, Sniffles, DeBreak, Picky, SVision, cuteSV, and cuteSV2 consumed less than 3.5 GB of memory, while pbsv consumed the most memory (30 GB, Additional file 1: Fig. S8d).

### Impact of sequencing platforms, depth, SV sizes, and supporting reads on the detection performance pipeline

We conducted a comparative analysis of Nanopore (R9, R10) and PacBio (CCS, CLR) data across multiple sample datasets using various pipelines (Additional file 1: Fig. S9). Our findings revealed that for DEL variant detection, the pipelines performed significantly better on CCS datasets than R9 and CLR (*p* = 8.1e − 8, *p* = 8.9e − 5, *t*-test) (Additional file 1: Fig. S9a). Similarly, the F1 score of pipelines for INS variant detection on real CCS datasets was significantly better than R9 and CLR (*p* = 2.9e − 11, *p* = 7.6e − 16, *t*-test) (Additional file 1: Fig. S9b). Additionally, while no significant differences were observed between the R9 and CLR datasets for DEL variant detection, significant differences were evident for INS variant detection (*p* = 5.7e − 9, *t*-test). However, no significant differences were observed in simulated data among the R9, R10, CLR, and CCS datasets for every SV type (Additional file 1: Fig. S9a–e).

We also investigated the effect of sequencing depth on the performance of the pipelines. Our findings indicate that higher sequencing depth can enhance pipeline recall by providing better coverage of SV signals. We evaluated four sequencing depths (5 × , 10 × , 15 × , 25 ×), revealing that the recall and F1 score at 10 × sequencing depth was approximately 17% and 8% higher than those at 5 × sequencing depth (Additional file 1: Fig. S10a,c). Moreover, increasing the sequencing depth from 10 × to 15 × and 25 × further improved recall and F1 score (recall: 4%, 3%; F1: 3%, 2%). Our results suggest that in scenarios where the cost of sequencing is directly proportional to the sequencing depth, opting for a sequencing depth of 10 × may offer a cost-effective solution (Additional file 1: Fig. S10). However, we highly recommend considering a sequencing depth of 15 × or higher for optimal performance if feasible.

We further explored the sensitivity of different aligners or callers to performance variations. Our analysis revealed that F1 measure fluctuations are greater for pipelines using the same aligner than those using the same caller in simulated and real data (Additional file 1: Fig. S11). This observation suggests that the choice of caller may be more influential than the choice of aligner in our current pipelines. Notably, pipelines utilizing callers such as cuteSV, cuteSV2, Delly, and Sniffles2 appear to be less affected by variations among aligners.

Our analysis examined the F1 scores for detecting SVs across five length range groups (50–100 bp, 100–500 bp, 500–1 kb, 1–2.5 kb, > 2.5 kb). Overall, most pipelines demonstrated consistent performance across varying SV sizes, indicating a lack of sensitivity to SV size. The F1 scores for different length ranges were largely similar (ranging from 0.7 to 0.8) among the pipelines, although they exhibited a slight increase for SVs longer than 2.5 kb (approximately 0.85–0.9, Additional file 1: Fig. S12). However, a few pipelines exhibited slightly higher sensitivity to SV size. For instance, NGMLR-DeBreak performed slightly worse than other pipelines in detecting deletions longer than 2.5 kb. NGMLR-SVIM showed lower F1 scores for insertions over 2.5 kb compared to other length ranges.

We also investigated how varying thresholds of minimal supporting reads affect performance in terms of the F1 score. In this analysis, we adjusted the filtering thresholds of minimal supporting reads for an SV from 2 to 20 across all simulated and real sample datasets with an average coverage of 25 in the CCS platform. The F1 score of most pipelines decreased as the minimal supporting read threshold increased, primarily due to a decrease in the recall rate (Additional file 1: Fig. S13c). A minimal supporting read threshold of 2–3 would be a suitable choice for quality control for a sequencing sample. Therefore, most evaluations in this paper were conducted based on the thresholds of minimal supporting reads of 2 unless specifically stated. However, for some pipelines, the F1 scores initially increased and then decreased with an increase in minimal supporting reads for INS variants, owing to the balance between precision and recall. This pattern was observed in pipelines associated with NanoSV, minimap2-SVision, and winnowmap-SVision. The optimal minimal supporting read threshold for detecting INS variants with these pipelines was 4–5 (Additional file 1: Fig. S13d).

### The accuracy of SV called breakpoint and length

**Fig. 2:**
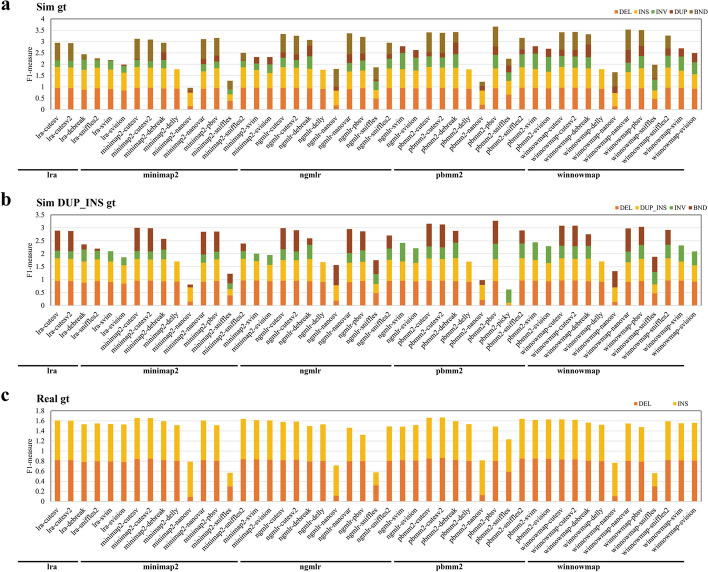
Performance of SV detection pipelines in different SV types (CCS). Precision and recall of DEL, DUP, INS, INV, and BND were determined with the simulated (**a**, **c** (consider genotypes)) and the real data (**b**, **d** (consider genotypes)). F1 measures, which combine precision and recall statistics (see the “Methods” section for details), are depicted for pipelines distinguished by different colored bars. Pipelines are categorized according to the alignment tools (lra, minimap2, ngmlr, pbmm2, winnowmap)

We also evaluated the deviations of breakpoints using Truvari on simulated data [39]. Across most pipelines, SV breakpoint deviations were detected on both the left and right sides within the − 50 to 50 bp range (Additional file 1: Figs. S14–15). When considering various types of SVs, we observed that the Sniffles and Sniffles2 callers performed exceptionally well for DEL variants, exhibiting more accurate breakpoint detection with fewer errors than other tools. Pipelines incorporating Pbsv demonstrated that 90% of breakpoint deviations for INS variants were concentrated between − 10 and + 10 bp. For INV SVs, pipelines related to Sniffles2, Picky, and SVIM displayed a high proportion of zero breakpoint deviations, ranging from 30 to 40%. Lastly, for DUP variants, pipelines associated with cuteSV, cuteSV2, Sniffles, Sniffles2, NanoSV, and Picky showcased high proportions of zero breakpoint deviations ranging from 40 to 60% (Fig. 2).

**Fig. 3:**
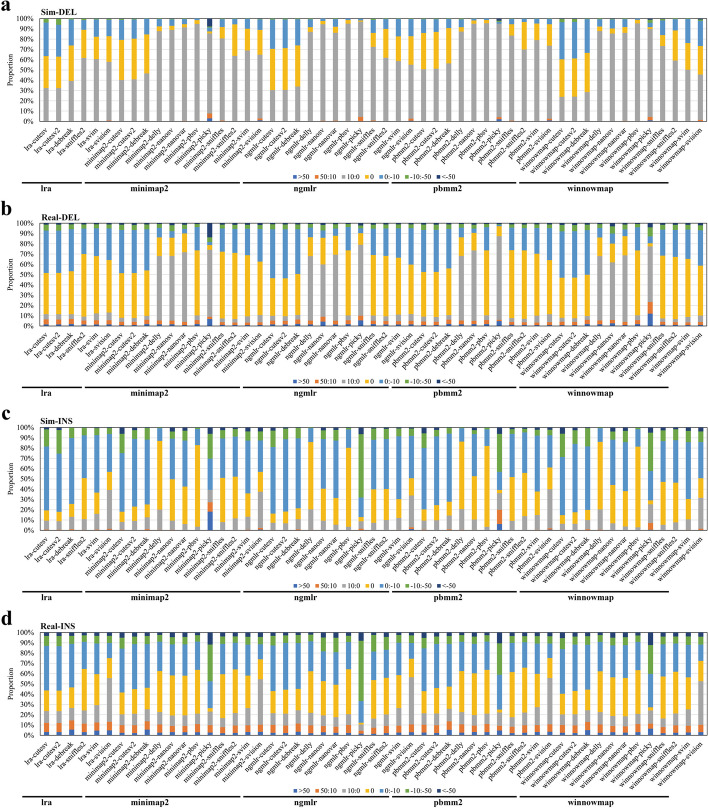
SV size errors of SV detection pipelines. SV size errors were determined with pipelines TP and reference SV difference from simulated (**a** DEL, **c** INS) and real data (CCS) (**b** DEL, **d** INS). The SV size error of pipelines TP SV was divided into seven groups (TP SV errors: 0:10, 10:50, > 50, 0, − 10:0, − 10: − 50, <  − 50). Statistics of SV size error (see the “Methods” section for details)

We then analyzed the length deviations called SVs. In simulated data, pipelines containing the callers cuteSV, cuteSV2, and DeBreak detected the highest proportion of DELs with zero SV size deviation, at approximately 40% (Fig. 3). Following them, the Sniffles2, SVIM, and SVision pipelines also identified a relatively high proportion of DELs with zero SV size deviation, ranging from 20 to 30%. In real data, pipelines linked to the callers cuteSV, cuteSV2, DeBreak, Sniffles2, SVIM, and SVision performed very well, detecting 40 to 60% of DELs with zero SV size deviation. For INS variants in simulated data, pipelines associated with the callers Delly, NanoSV, Nanovar, Pbsv, Sniffles, and Sniffles2 exhibited smaller length deviations (Additional file 1: Fig. S12). Among these pipelines, the proportion of INS with zero deviation ranged from 20 to 70%. Specifically, pipelines linked to the callers Delly and Pbsv demonstrated the best performance, with 60 to 70% of INS having zero deviation. Similarly, in real data, pipelines associated with Delly and Pbsv also detected a higher proportion of INS with zero SV size deviation, ranging from 60 to 70%.

**Fig. 4:**
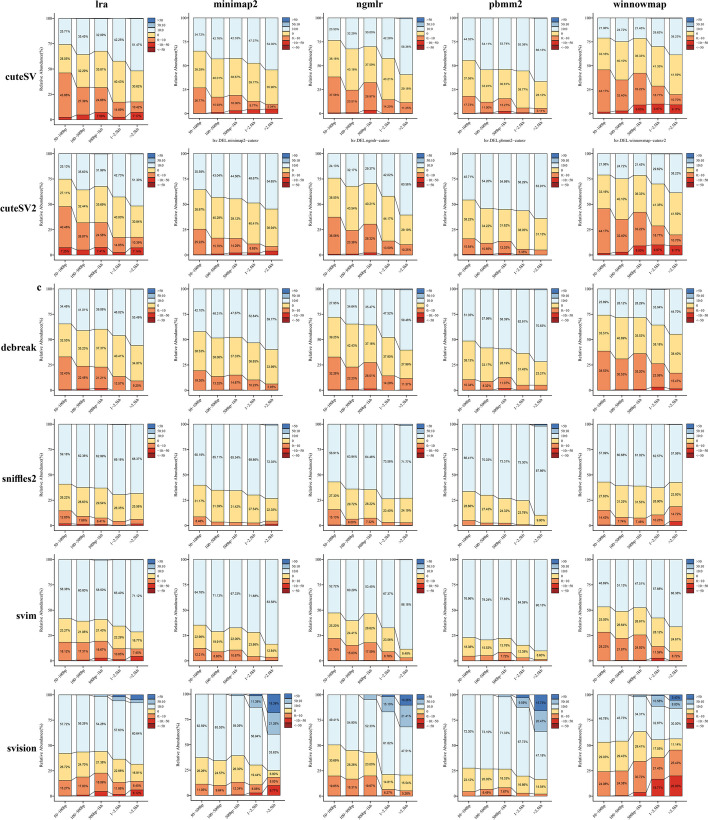
Distribution of SV length range and length deviation of DEL in different pipelines. The legend depicts colors ranging from deep blue to red, representing different deviation scales (> 50 bp, 50:10 bp, 10:0 bp, 0 bp, 0: − 10 bp, − 10: − 50 bp, <  − 50 bp). The x-axis represents five length intervals of SV size (50–100 bp, 100–500 bp, 500–1 kb, 1–2.5 kb, > 2.5 kb). The y-axis represents the proportion of different deviation scales within the corresponding length ranges

We also observed that the length of the SV influences the size and breakpoint deviations of SVs detected by pipelines. In simulated data, for DEL variant detection, pipelines such as LRA-cuteSV, LRA-cuteSV2, winnowmap-cuteSV, winnowmap-cuteSV2, LRA-SVision, minimap2-SVision, and winnowmap-SVision exhibit greater SV size deviations when the SV length exceeds 2.5 kb (Fig. 4). In these cases, the proportion of DELs with length deviations between − 10 and − 50 bp and greater than 50 bp ranges from 6 to 20%. However, pipelines associated with DeBreak, Sniffles2, and SVIM show more stable SV size deviations across different SV length ranges, primarily fluctuating within the − 10 to 10 bp range, indicating smaller deviations. For INS variant detection in simulated data, pipelines from callers cuteSV, cuteSV2, and Sniffles2 detect INS with a higher proportion of SV size deviations in the − 10 to − 50 bp range when the SV length exceeds 2.5 kb (Additional file 1: Fig. S16). These findings suggest that INS detected by pipelines tend to have larger length deviations than DELs at greater SV lengths (> 2.5 kb). Similarly, breakpoint deviations in pipelines are also affected by SV length. Pipelines utilizing aligners NGMLR and LRA exhibit a higher proportion of breakpoint deviations in the ranges of 10 to 50 bp and − 10 to − 50 bp for both DELs and INS compared to other pipelines, and these deviations are more influenced by SV length (Additional file 1: Fig. S17).

### Accuracy of SV genotype calling

In genetic and functional studies of SVs, accuracy in SV genotyping is crucial, alongside the ability to distinguish SV loci. Therefore, we evaluated the performance of SV callers from the perspective of genotype consistency (Fig. 2, Additional file 1: Fig. S18). In the simulated dataset, pbmm2-pbsv exhibited the highest accumulated F1 measure (~ 3.6) for the five type SVs, establishing itself as the top genotype calling pipeline in the evaluation (Fig. 2). Additionally, pipelines utilizing callers such as cuteSV, cuteSV2, DeBreak, pbsv, and Sniffles2 also demonstrated strong performance in SV genotyping accuracy (F measure > 3). Notably, DeBreak-related pipelines exhibited high F1 measure levels, particularly pbmm2-DeBreak (2.96) and winnowmap-DeBreak (2.87), when the BND type was not included. Remarkable F1 measure levels for DEL’s and INS’s genotype calling were observed in pipelines such as pbmm2-cuteSV, LRA-cuteSV, minimap2-Sniffles2, and pbmm2-Sniffles2. For INV’s genotype calling, NGMLR-SVIM (0.71), pbmm2-SVIM (0.68), and pbmm2-SVision (0.65) exhibited better performance compared to other pipelines. The evaluation results in real data mirrored those in the simulated dataset. We found that pipelines using callers like cuteSV, cuteSV2, and Sniffles2 demonstrated high F1 measure levels for DEL and INS variants in real data (F measure > 1.6) (Fig. 2). Strong performers among these pipelines included pbmm2-cuteSV2 (1.67), pbmm2-cuteSV (1.66), minimap2-cuteSV (1.66), minimap2-cuteSV2 (1.66), and minimap2-Sniffles2 (1.64).

### MIER level of different detection pipelines

**Fig. 5:**
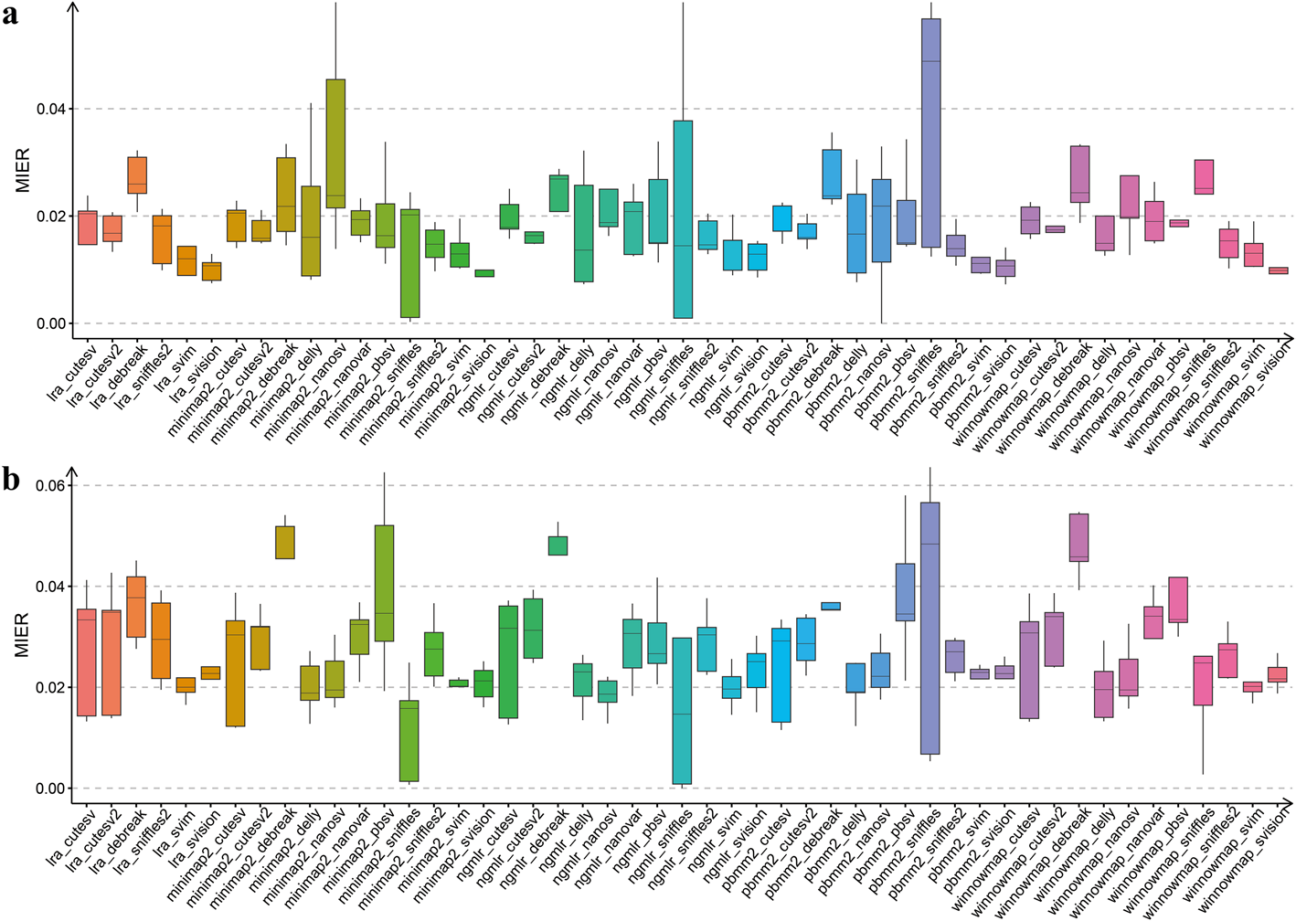
SV MIER (Mendelian error rate) of SV detection pipelines in pedigree. SV detection pipelines MIER was determined with pedigree data (ONT, CCS, CLR: HG002, HG003, HG004; CCS: HG005, HG006, HG007) in different SV types (DEL (**a**), INS (**b**)). Statistics MIER (see the “Methods” section for details)

We further compared the MIER of different pipelines to evaluate the accuracy of their genotyping in trios with real sequencing data. Our findings indicate that the caller is the primary factor influencing MIER levels. Overall, the evaluated pipelines exhibited MIER levels of less than 10%, with some outstanding pipelines achieving MIER levels of around 2% (Fig. 5). Specifically, for detecting DELs, pipelines associated with the callers cuteSV2, Sniffles2, SVIM, and SVision demonstrated MIER levels below 2%. Similarly, for INS variant genotyping, pipelines associated with SVIM and SVision showed low MIER levels (~ 2%) and robust performance (Fig. 5). Generally, the MIER levels for INS variant detection were slightly higher than those for DEL variant detection, as observed in pipelines like minimap2-cuteSV2, minimap2-SVIM, and NGMLR-SVision. Additionally, in the detection of INV and DUP, pipelines associated with the callers DeBreak, Sniffles2, SVIM, and SVision exhibited very low MIER levels (~ 0%), such as LRA-cuteSV2, minimap2-cuteSV2, NGMLR-NanoSV, NGMLR-Sniffles2, pbmm2-DeBreak, and pbmm2-SVision (Additional file 1: Fig. S19). The lower MIER levels for INV and DUP variant detection than DEL and INS detection are likely due to the lower number of detected SVs in INV and DUP categories.

In addition to the pipelines’ impact on MIER levels, other factors also play a role, such as the choice of “minimum support read number” and the reference genome version. First, we observed the impact of the “minimum support read number” on the MIER levels of the pipelines. For DEL variants, the MIER initially decreased as the minimum support read number increased from 2 to 5, then increased as the number rose from 5 to 8. For INS variants, the MIER gradually decreased with increasing minimum support read number. Finally, for INV and DUP, increasing the minimum support read number led to a slow rise in MIER (Additional file 1: Fig. S20). Regarding the influence of the reference genome on the MIER levels of the pipelines, we selected a set of high-performing pipelines (aligners: minimap2, winnowmap; callers: cuteSV, DeBreak, Sniffles, SVision) to evaluate their MIER levels based on the T2T genome. We found that the MIER levels of these pipelines for detecting of DEL and INS variants were consistent with those based on the GRCh38 genome. However, for DUP and INV, these pipelines exhibited MIER levels 10–30% lower than those based on the GRCh38 genome (Additional file 1: Figs. S19 and S21).

### Pipeline results merging to improve performance

**Fig. 6:**
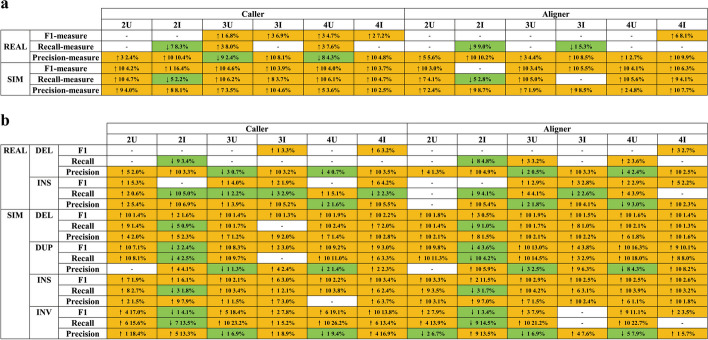
The significance of F1, recall, and precision for different SV types in the top 10 combined pipelines compared to the individual pipelines that constitute the combination. **a** Overall improvement levels of DEL and INS in the top 10 combined pipelines across different combination methods in both simulated and real datasets. **b** Improvement levels of different SV types in the top 10 combined pipelines across various combination methods in simulated and real datasets. “Caller” represents combined pipelines based on the same aligner with different callers. In contrast, “Aligner” represents combined pipelines based on the same caller with different aligners. The table header format, such as “2U, 2I, 3U…,” indicates the combination method: the first number represents the number of combined pipelines, and the second character indicates whether the combination is based on union (U) or intersection (I). On the left side of the table, “REAL” denotes real data, and “SIM” denotes simulated data. The arrows in the table represent whether the performance of the combined pipeline increased or decreased compared to the individual pipelines that constitute the combination. The integer following the arrow indicates the number of combined pipelines with and without significance after merging in the top 10 combinations. The final percentage represents the extent of the performance improvement of the combined pipeline compared to the individual pipelines. Lastly, white shading represents combinations with no significance, orange indicates a significant improvement in performance, and green denotes a significant decrease in performance

Some studies employ a combination of multiple pipelines or algorithms to enhance the detection accuracy of SV calling. However, the optimal strategy for merging multiple pipelines based on TGS data has not been systematically investigated. To address this gap, we calculated the accuracy, recall, and median F1 scores of merged pipelines consisting of two, three, and four individual pipelines, employing different merge strategies for various SV types (DEL, INS, INV, DUP; Fig. 6). Specifically, to assess the influence of aligners and callers on the merged results, we categorized our pipeline combinations into two groups: pipelines with the same aligner but different callers (the caller-based combination group) and pipelines with the same caller but different aligners (the aligner-based combination group). Moreover, considering that the choice of merge strategy (intersection or union) can significantly affect the results, we employed “minimum number of supporting caller” values of 1 (union) and 2 (intersection) when using SURVIVOR to combine the results of two or more pipelines. Consequently, the combination categories were designated as < caller/aligner >  < pipelines number >  < union/intersection > , for example, caller_2U, caller_2I, aligner_3U, etc. Due to the large number of possible combinations resulting from the 41 pipelines (see “Methods”), we ranked them based on the median F1 score of the combined pipelines and focused our analysis on the top 10 combinations.

The performance enhancement achieved by combined pipelines was more pronounced in simulated data than in real data, although pipeline combinations consistently improved performance (Fig. 6, Additional file 1: Fig. S22). For instance, in real data, the caller_2U combination improved precision by only 2.4%, with no significant changes in F1 score and recall before and after the pipeline merge. In contrast, in simulated data, there were moderate improvements across all metrics: F1 score (4.7%), recall (4.7%), and precision (4%) (Fig. 6a). The enhancement of the caller-based and aligner-based combination groups was very similar in simulated data (Fig. 6, Additional file 1: Fig. S22). However, in real data, these two groups exhibited slight differences. Specifically, the 3U and 4U combinations based on caller focused more on improving recall, while those based on aligners emphasized enhancing precision. Incorporating more pipelines led to greater improvements regarding the number of combined pipelines. In addition, choosing the appropriate merging strategy (union or intersection) based on the number of pipelines being combined was crucial. When the number of pipelines was small, for instance, two, using the union strategy might result in more significant improvements than the intersection strategy. Conversely, if three or more pipelines were used, the intersection strategy enhanced performance (Fig. 6).

The analysis showed that pbmm2 had the highest frequency among the top ten combined pipelines regardless of the sequencing platforms (ONT: 0.451, CCS: 0.452, CLR: 0.455, Additional file 1: Fig. S23a). Following pbmm2, LRA (CCS: 0.153), minimap2 (CLR: 0.261), and winnowmap (ONT: 0.19) were the aligners with the second-highest frequencies in the ten combined pipelines. Additionally, we noticed that the choice of aligner in combined pipelines displayed specificity in detecting specific SV types. For instance, pbmm2 was more prevalent in the top 10 merged pipelines for DEL (0.5) and INS variants (0.78) (Additional file 1: Fig. S23c). Conversely, NGMLR (0.97) was the most common aligner in the top 10 merged pipelines for INV, while winnowmap (0.93) was frequently observed in pipelines targeting DUPs.

Finally, we compiled the frequency of variant callers among the top 10 merged pipelines. Among the combined pipelines (sorted by median F1 measure for DEL and INS), callers such as cuteSV (0.17–0.20), cuteSV2 (0.14–0.17), DeBreak (0.23–0.27), and Sniffles2 (0.07–0.16) exhibited higher frequencies (Additional file 1: Fig. S23b). Moreover, the distribution of callers varied among the top 10 merged pipelines for different SV types. Among the top 10 merged pipelines (ranked by median F1 score) for various SV types, cuteSV2 and SVIM were more frequently observed for detecting DEL (cuteSV2: 0.272, SVIM: 0.25). For INS variant detection, callers cuteSV, cuteSV2, and DeBreak had higher frequencies among the top 10 merged pipelines (cuteSV: 0.18, cuteSV2: 0.16, DeBreak: 0.17, Additional file 1: Fig. S23d). Additionally, callers SVIM (0.24) and SVision (0.23) displayed elevated frequencies for INV. In the case of DUP, callers cuteSV (0.18), cuteSV2 (0.19), and pbsv (0.18) exhibited higher frequencies in the top 10 merged pipelines (Additional file 1: Fig. S23d). Consequently, these callers with a high frequency among the top 10 merged pipelines should be given higher priority when considering pipeline combinations to enhance the performance of SV detection in TSG data.

## Discussion

In this study, we conducted a comprehensive performance assessment of 53 widely used SV detection pipelines. These pipelines involved five aligners and 12 callers, all based on TGS. Our comparative study addressed limitations in previous research, which often used limited TGS data or fewer pipelines. We also considered multiple important factors related to SV calling, including SV length, breakpoint deviation, genotyping accuracy, runtime, and memory consumption. Our findings offer valuable insights into detecting SVs in TGS data, helping researchers select appropriate pipelines. Some results from this study align with previous research. First, the relative performance of different pipelines shows moderate consistency across most simulated and real datasets. For example, the Spearman correlation of F1 values of pipelines’ performance ranged from 0.4 to 0.7 for most SV types across different sequencing platforms (Additional file 1: Fig. S24). However, there is a decline in precision and recall when comparing real data to simulated data. Similar observations were reported by previous studies [11, 27]. The complexity of SVs in real data may contribute to this decline, as real SVs tend to be more intricate than simulated ones. Second, most pipelines perform well in detecting DEL and INS (F1: 0.80–0.92) but have lower performance for INV and DUP (F1: 0.6–0.7). The latest SV detection tool, SVision, can identify complex SVs directly [21]. In contrast, previous callers may break down a complex SV into multiple simple types such as DEL, INS, INV, and DUP. Finally, in TGS, pipeline performance is not highly sensitive to SV size. The precision and recall of pipelines only show relatively mild changes when dealing with SVs of different lengths. This observation is generally consistent with conclusions from SV detection algorithms based on SGS [27].

Importantly, our study presents several notable findings compared to previous research [23, 26–28, 33, 47]. First, we reveal various biases in the length and position of SVs detected by different pipelines. Although most called SVs have small lengths and breakpoint deviations (less than 50 bp), the length and location of SVs are crucial parameters in certain tools for SV pathogenicity prediction and annotation [48–53]. We found that the original size of the SV influences the biases in called SV length and breakpoints, and these biases are more sensitive to callers than to aligners. Additionally, the degree of biases varies across different types of SVs. These biases may be attributed to sequencing errors, genomic complexity (repetitive sequences), the inherent complexity of SVs, the accuracy of aligners, and the SV signal processing methods used by callers [7, 10]. Furthermore, we evaluated the performance of pipelines for genotype calling in both simulated data and real family datasets. We found that cuteSV, cuteSV2, Sniffles2, SVision, and SVIM achieved smaller MIERs, ranging from approximately 2 to 7%, indicating more accurate genotype calling than other pipelines.

In our analysis of the performance of 53 pipelines, we found that callers contribute more to the variation in performance than aligners. In a pipeline, the aligner critically influences SV signals’ presence, location, and strength. However, the caller is essential for clustering SV signals, filtering signals, and identifying SV types, which are all crucial for SV detection. This was confirmed when we merged results from multiple pipelines. In our analysis of merging results based on callers and aligners, we found that the performance improvement was similar for both methods. However, combining multiple pipelines based on different callers is more feasible considering runtime efficiency. Additionally, when merging a small number of pipelines (e.g., two pipelines), using a union may be more effective than an intersection. In contrast, when merging more pipelines, the intersection method is more reliable. This is likely because, when combining two pipelines using the union method, the number of correct SVs shared between the two pipelines is greater than the number of incorrect SVs. However, as the number of combined pipelines increases, the rate of incorrect SVs grows faster than that of correct SVs.

When analyzing the pipeline-combination results, we observed that both caller-based combinations (with aligner fixed) and aligner-based combinations can similarly improve performance. However, we also noted that the performance variability due to callers was greater than that due to aligners among the 53 individual pipelines. This difference may stem from the greater variability among the twelve callers compared to the five aligners in the 53 pipelines. Thus, the choice of callers leads to greater performance variability in pipelines than the choice of aligners. Despite this, when merging results based on callers and aligners, we selected combinations from the top 10 performing pipelines, excluding those associated with weaker callers. This selection resulted in similar performance improvements when comparing the caller-based to the aligner-based combination results. Nevertheless, considering computational storage and time constraints, merging multiple pipelines based on different callers is more feasible. Additionally, when merging a small number of pipelines (e.g., two pipelines), using a union method might be more effective than an intersection method. Conversely, the intersection method is more reliable for merging more pipelines. This is likely because, when combining two pipelines using the union method, the number of correctly identified SVs shared between the two pipelines is greater than the number of incorrect SVs. However, as the number of combined pipelines increases, the growth rate of incorrect SVs exceeds that of correct SVs.

Our study has several areas that need improvement. First, due to the lack of benchmark datasets for DUP, INV, and TRA in real data, evaluating pipeline performance for these SV types only based on simulated data may introduce bias. For example, in real data, the detection performance for DEL and INS variants by the pipelines is approximately 10% lower than in simulated data. Additionally, the datasets used in our experiments might be limited in scale and representativeness. Due to resource constraints, we only utilized a few publicly available datasets, which might restrict the generalizability of our findings. Future research should incorporate larger and more diverse datasets to enhance the reliability and generalizability of the results.

## Conclusions

To our knowledge, this study represents the most extensive analysis of genomic SV pipelines based on TGS data to date. Our evaluation demonstrates that the choice of the caller is a critical factor influencing the accuracy of SV detection pipelines more than the choice of the aligner. In our comparison, three callers—cuteSV2, DeBreak, and SVision—performed the best. Regarding computational resources, Sniffles2 exhibited the lowest memory usage and fastest processing speed, making it highly suitable for large-scale population studies. We also found that merging SVs identified by multiple pipelines using the aligners pbmm2 and winnowmap significantly improves accuracy compared to other aligners. However, we noted that the genotype accuracy of SVs in TGS still requires improvement despite the higher recall and precision observed in SV detection pipelines (e.g., minimap2-cuteSV2, winnowmap-NanoVar, and winnowmap-Sniffles2). Additionally, the ranking of specific pipelines highly depends on various factors, such as specific SV types, deviations, and genotyping accuracy, indicating that no universally best pipeline exists. To aid in selecting top-performing pipelines from different perspectives, we have summarized the rankings and performance metrics into a comprehensive online table for flexible queries (http://pmglab.top/SVPipelinesRanking).

## Methods

### SV benchmark datasets

We used Visor software [42] to simulate reads based on the GRCh38 reference genome. First, we used the HACk module in the Visor software to generate genome haplotypes, including virtual SV records. Next, we used the LASeR module to select the corresponding error_model and qscore_model to generate reads for long reads for different platforms (model parameters: ONT10.4: nanore2023; ONT9.4: nanore2020; CLR: pacbio2016; CCS: pacbio2021).

For the analysis of real data reads, we utilized the HG002 family pedigree data (HG002 [son], HG003 [father], HG004 [mother]) comprising ONT, CLR, and CCS technologies sourced from the NCBI Ashkenazim Trio dataset [54]. Additionally, the HG005 family pedigree data [54] (HG005 [son], HG006 [father], HG007 [mother]), which includes ONT, CLR, and CCS technologies, was sourced from the NCBI Chinese Trio database. The ONT and CLR data for the NA12878 sample were sourced from NCBI references [55–58]. We also incorporated samples HG00096 (ONT, CCS) and HG00512 (ONT, CCS, CLR) from the Human Genome Structural Variation Consortium (HGSVC) database [31]. To assess the impact of sequencing depth on SV detection, we established four sequencing depths: 5 × , 10 × , 15 × , and 25 × .

SV benchmark construction for the samples relied on public datasets. For the HG002 sample, DEL and INS variants, along with high-confidence regions, were sourced from the GIAB database [29]. Subsequently, using LiftOver, we converted the HG002’s hs37d5 version SV benchmark and high-confidence regions to the GRCh38 reference. For the CHM13 sample, DEL and INS variants were sourced from the human pangenomics database [59]. Similarly, DEL and INS variants for samples HG00096, HG00512, and NA12878 were obtained from the HGSVC database [60]. For all SV benchmarks, we retained only SV records located on chromosomes 1–22, X, and Y.

### Pipeline construction and SV detection

We generated alignment indexes for the GRCh38 human genome using aligners. The pipelines were constructed using aligners and callers, with most parameters set to default values. The unified parameters for callers were set as follows unless specifically stated: SV length ≥ 30 and minimum support read number ≥ 2 (see supplementary materials for more details). Preliminary experiments were conducted on TGS datasets using the following server environment: 4*Intel(R) Xeon(R) Gold 6148 CPU @ 2.40 GHz, Memory: 1007G, Hard disk storage: 328 T. These experiments aimed to assess the feasibility of pipelines and eliminate slow and infeasible ones. The pipelines that passed the assessment were defined as rules, and we utilized Python to generate analysis scripts for SV detection in the datasets (https://github.com/liuz-bio/SVPipelinesEvaluation.git).

### SV call set filtering

The variation in the output file formats of different callers poses a challenge when comparing pipelines. To mitigate this issue, we standardized the VCF file formats of callers by selectively extracting and formatting essential SV record information, including CHROM, POS, END, SVTYPE, SVLEN, SUPPORTREADS, and GT. Notably, SVision exhibits a more sophisticated ability to identify SV types [21]. In real data, SVision often presents a combination of multiple simple SVs, such as DEL + DUP, DEL + INV + DUP, and INS + tDUP. We decomposed these complex SV combinations into simple types for further evaluation, including DEL, INS, DUP, and INV.

Moreover, if SV records from different callers have “DUP” in the SVTYPE field, they are considered duplications. On the other hand, because some callers might classify DUP as INS, we merge DUP and INS as INS for evaluation, labeled as DUP_INS. Our filtering criteria retained only variants marked as “PASS” in the “FILTER” field (In SVision, the “FILTER” field does not use “PASS”; we choose “Covered” as the “PASS” record.), with a minimum support read number of 2 and genotype of alternative alleles. We created separate VCF files for different SV types and minimum support read number ranges (2–20), facilitating further evaluation and analysis.

### Pipeline evaluation

We employed Truvari [39] to evaluate the accuracy, recall, and F1 score of pipelines across different SV types and their performance regarding varying SV sizes and minimum support read numbers. We generated a BED file for each SV benchmark set covering a 500 bp range upstream and downstream of each SV, which we defined as high-confidence regions. We then compared the pipeline SVs within these high-confidence regions to the benchmark SVs. Using Truvari [39], we computed the accuracy, recall, and F1 scores for DEL, INS, INV, DUP, and DUP_INS SV types. In the case of translocations, SVs detected by the pipeline meeting the conditions of Eq. 1 were considered true positive (TP) calls at the breakpoint level; otherwise, they were classified as false positives (FP). A ground benchmark SV was labeled as a false negative (FN) if no SV call satisfied Eq. 1, following the method proposed by Jiang et al. [11]. Furthermore, we calculated accuracy, recall, and F1 score for BND based on Eqs. 2–4. Additionally, we merged the Truvari results with the “tp-base.vcf” and “tp-call.vcf” files using SURVIVOR [61] to establish correspondence between the SV benchmark and pipeline SVs. By analyzing these correspondences, we computed the length and breakpoint deviations between SV benchmark and pipeline SVs. To further explore the impact of SV length on the accuracy, recall, F1 score, breakpoints, and length deviation of the pipelines, we categorized each SV type into five size gradients: 50–100 bp, 100–500 bp, 500 bp–1 kb, 1–2.5 kb, and > 2.5 kb. We then evaluated the performance of the pipelines within each length range for each SV type. Moreover, we recognized the significance of the minimum supporting read number as a crucial factor influencing pipeline performance. Therefore, we categorized the SVs based on the “minimum supporting read number” for each SV type within the pipeline, ranging from 2 to 20, to assess the performance of the pipelines across different minimum support read number for various SV types.

1 $$\left\{\begin{array}{c}\left|{comp}_{BK1}-{base}_{BK1}\right|\le 1kb\\ \left|{comp}_{BK2}-{base}_{BK2}\right|\le 1kb\\ {comp}_{chr1}={base}_{chr1}\\ {comp}_{chr2}={base}_{chr2}\end{array}\right.$$

where “comp” refers to the pipeline, while “base” refers to the benchmark. “BK1,” “BK2,” “Chr1,” and “Chr2” represent breakpoints and chromosomes.

Prediction is defined as:

2 $$Precision=\frac{TP}{TP+FP}$$

Recall is defined as:

3 $$Recall=\frac{TP}{TP+FN}$$

F1 score is defined as:

4 $$F1=\frac{2*Precision*Recall}{Precision+Recall}$$

### Calculate the runtime and memory usage of aligners and callers

We used a sample HG002 with an average sequencing depth of 5 × for ONT, CLR, and CCS to assess aligners’ and callers’ runtime and memory consumption. Our computer setup included 2*AMD EPYC 7601 CPUs running at 2.2 GHz, 128 threads, and 224 GB of memory. Callers and aligners utilize the maximum number of available threads during runtime if they support multithreading. We used the Linux command “/usr/bin/time -o < output > -v < aligner/caller command > ” to track the memory usage (maximum resident set size) and execution time (elapsed wall clock time). Each command was repeated three times to collect data.

### Mendelian error rate calculation

Children primarily inherit SVs from their parents, with minimal de novo SVs [32, 62, 63]. A comprehensive study involving 2396 families based on SGS data revealed a de novo structural mutation rate ranging from 0.160 to 0.206 events per genome [64]. Therefore, pipeline performance and genotype accuracy can be evaluated by analyzing Mendelian errors in SVs called among family members. In our assessment, we utilized datasets from families (HG002, HG003, HG004 with ONT; HG005, HG006, HG007 with ONT, CLR, and CCS) to gauge the level of MIER for a pipeline. We employed the “merge” function of the SURVIVOR tool to combine SV records of fathers, mothers, and children within each family based on specific pipeline parameters (minimum number of supporting caller: 1, max distance between breakpoints: 100). We selected all SV records with several supporting callers of 3 and non-empty sample genotypes from the merged SV records. Any SV record where the parent’s genotypes did not match the child’s genotype according to Mendelian inheritance laws was classified as a Mendelian error. We used the minimum supporting read number as a filtering parameter to examine its impact on the MIER level. Specifically, for the MIER calculation, we considered only SVs where the minimum supporting read number for both the child and the parents exceeded the threshold ranging from 2 to 20.

### Multi-pipeline results merge

Based on the pipeline evaluation results, we selected 41 pipelines with superior performance for result-merging analysis, comprising nine callers (cuteSV, cuteSV2, DeBreak, NanoVar, pbsv, Sniffles, Sniffles2, SVIM, SVision) and five aligners (LRA, minimap2, NGMLR, pbmm2, winnowmap). Initially, we standardized the results of the 41 pipelines by selectively extracting and formatting the SV record information. We then constructed combination schemes based on caller combinations (with aligner fixed) and aligner combinations (with caller fixed). Next, we divided the combination schemes based on whether SURVIVOR was used to merge multi-pipeline results using the union method (“minimum number of supporting caller: 1”) or the intersection method (“minimum number of supporting caller: 2”). SURVIVOR’s parameter “max distance between breakpoints” was set to 500 bp. We set the number of combined pipelines to 2, 3, and 4. This resulted in 12 combination schemes in total: 2 (caller/aligner) * 2 (“minimum number of supporting caller”: 1 or 2) * 3 (number of pipelines: 2, 3, 4), labeled as < caller/aligner >  < 2,3,4 >  < Union/Intersection > . We used SURVIVOR to merge VCF output files from multiple callers. When merging VCF files from multiple pipelines, SURVIVOR consolidated SVs within the specified “max distance between breakpoints” range into a single SV and used the “minimum number of supporting caller” parameter to determine whether the merged SV met the criteria for output. For example, when merging SV results from 3 pipelines with “minimum number of supporting caller” set to 2, an SV record was retained if at least two pipelines supported it; otherwise, it was removed from the output. We evaluated the merged results on both real and simulated data. The SV (DEL, INS) benchmarks for real data were derived from public datasets. The merging and evaluation of multi-pipeline results were conducted separately for each SV type. After evaluation, we ranked the combined pipelines for real and simulated data based on the median F1 scores across different samples and sequencing data types. The top 10 pipelines for each combination scheme were then collected for analysis. We used the Mann–Whitney *U* test to determine if there were significant differences in F1, recall, and precision between single pipelines and combined pipelines among the top 10 combinations across different samples and sequencing data types. We also counted the number of significant combination pipelines and assessed the level of difference in F1, precision, and recall measures between real and simulated data for evaluation.

### Supplementary Information

Additional file 1. Supplementary figures. It contains all supplementary figures and figure legends.

Additional file 2: Tables S1–S3. It contains versions of aligners and callers (Table S1), the number of SV benchmarks in simulated and real data (Table S2), and the read length of dataset samples (Table S3).

Additional file 3: Table S4. The proportions of pipelines reporting DUP as INS and INS as DUP under different sequencing platforms.

Additional file 4: Table S5. Precision, recall, and F1 scores for different samples from various sequencing platforms at a sequencing depth of 25× under different minimum support numbers.

Additional file 5. Detailed parameters for generating simulated TGS reads and constructing pipelines.

Additional file 6. Review history.

## Data Availability

The code presented in the paper has been implemented and is available for public access on GitHub (https://github.com/liuz-bio/SVPipelinesEvaluation.git) and the Zenodo repository [65] (10.5281/zenodo.11351869). The code is distributed under the MIT open-source license [66]. The data underlying this article are available in the article and its online supplementary material. Other data are stored in the Zenodo (10.5281/zenodo.11351869) repository [65]. The hg38 human reference genome is downloaded from IGSR [67]: ftp://ftp.1000genomes.ebi.ac.uk/vol1/ftp/data_collections/HGSVC2/technical/reference/20200513_hg38_NoALT/hg38.no_alt.fa.gz. HG002 family pedigree data [68–72, 73] (HG002 [son], HG003 [father], HG004 [mother], including ONT, CLR, and CCS) obtained from the NCBI Ashkenazim Trio dataset: https://ftp-trace.ncbi.nlm.nih.gov/giab/ftp/data/AshkenazimTrio/. The HG005 family pedigree data [74–78] (HG005 [son], HG006 [father], HG007 [mother], including ONT, CLR, and CCS) were obtained from the NCBI Chinese Trio database: https://ftp-trace.ncbi.nlm.nih.gov/giab/ftp/data/ChineseTrio/. The data for the CHM13 sample were collected from Nanopore [79] (https://s3-us-west-2.amazonaws.com/human-pangenomics/T2T/CHM13/nanopore/rel2/), PacBio CCS (SRR11292120, SRR11292121, SRR11292122, SRR11292123), and CLR [79] (https://s3-us-west-2.amazonaws.com/human-pangenomics/T2T/CHM13/assemblies/alignments) sources. The NA12878 sample’s ONT and CLR data were obtained from NCBI (https://ftp-trace.ncbi.nlm.nih.gov/giab/ftp/data/NA12878/) [80]. We also introduced samples HG00096 (ONT, CCS) and HG00512 (ONT, CCS, CLR) from the Human Genome Structural Variation Consortium (HGSVC) database [81] (http://ftp.1000genomes.ebi.ac.uk/vol1/ftp/data_collections/HGSVC3). DEL and INS variants and high-confidence regions in the HG002 sample were obtained from the GIAB database [82] (https://ftp-trace.ncbi.nlm.nih.gov/giab/ftp/data/AshkenazimTrio/analysis/NIST_SVs_Integration_v0.6/). DEL and INS variants in the CHM13 sample were obtained from the human pangenomics database [83] (https://s3-us-west-2.amazonaws.com/human-pangenomics/T2T/CHM13/assemblies/variants/CHM13_to_GRCh38/chm13v1.0_with38Y_to_GRCh38.dip.vcf.gz). DEL and INS variants of HG00096, HG000512, and NA12878 samples were obtained from the HGSVC database [84] (https://ftp.1000genomes.ebi.ac.uk/vol1/ftp/data_collections/HGSVC2/release/v1.0/integrated_callset/).
