# Supplementary material for: Comprehensive and deep evaluation of structural variation detection pipelines with third-generation sequencing data
Source: Genome Biol. 2024 Jul 15;25:188. doi: 10.1186/s13059-024-03324-5 (PMC11247875; doi:10.1186/s13059-024-03324-5)
Supplement: Supplementary file 1 — Additional file 1. Supplementary figures. It contains all supplementary figures and figure legends. [file 13059_2024_3324_MOESM1_ESM.pdf]

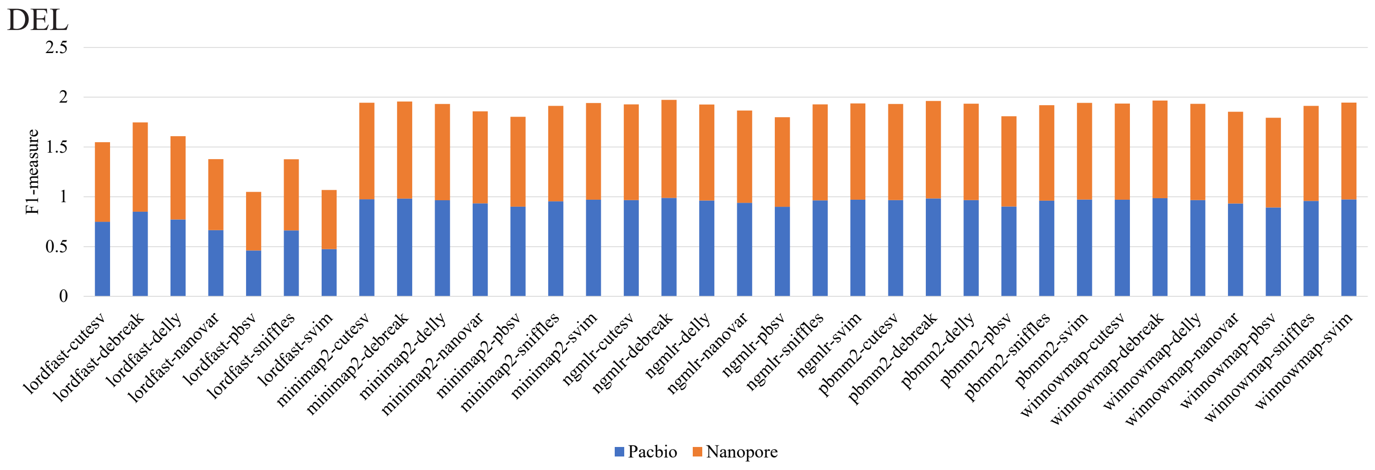


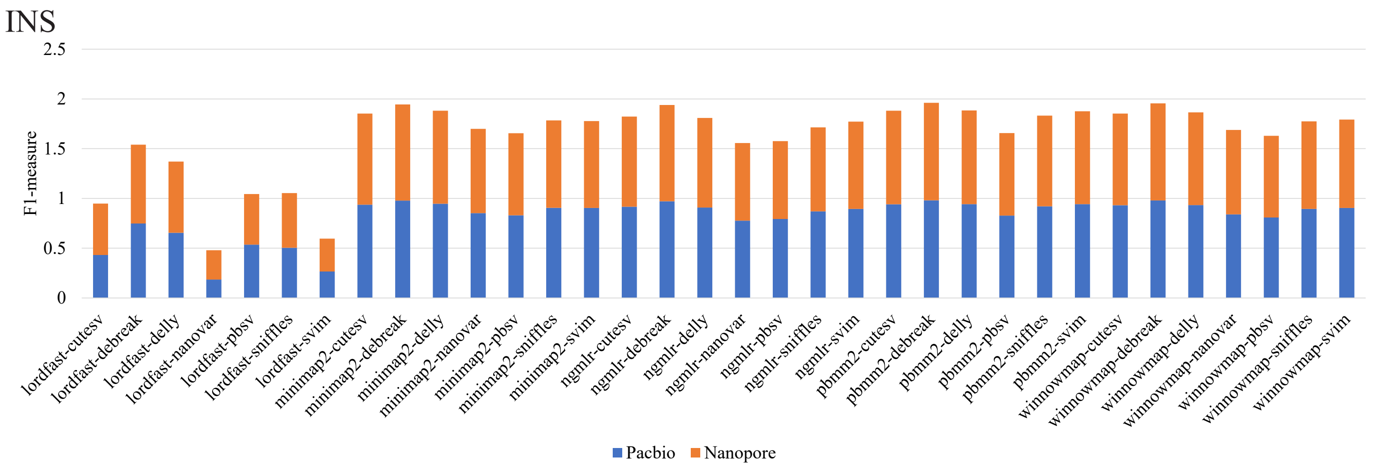


**Fig S1.** Performance testing of aligner-related pipelines for DEL and INS detection.

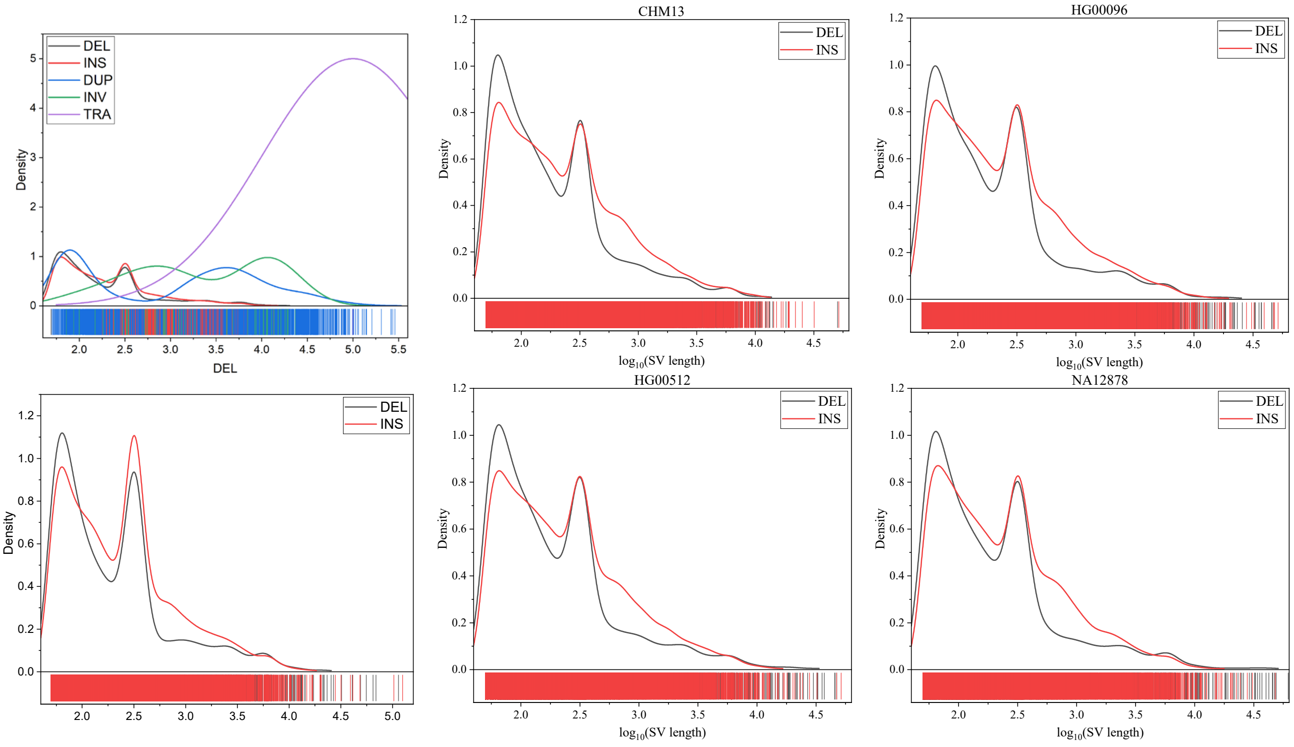


**Fig S2.** The SV length distribution in benchmark datasets includes both simulated (Sim) and real samples.

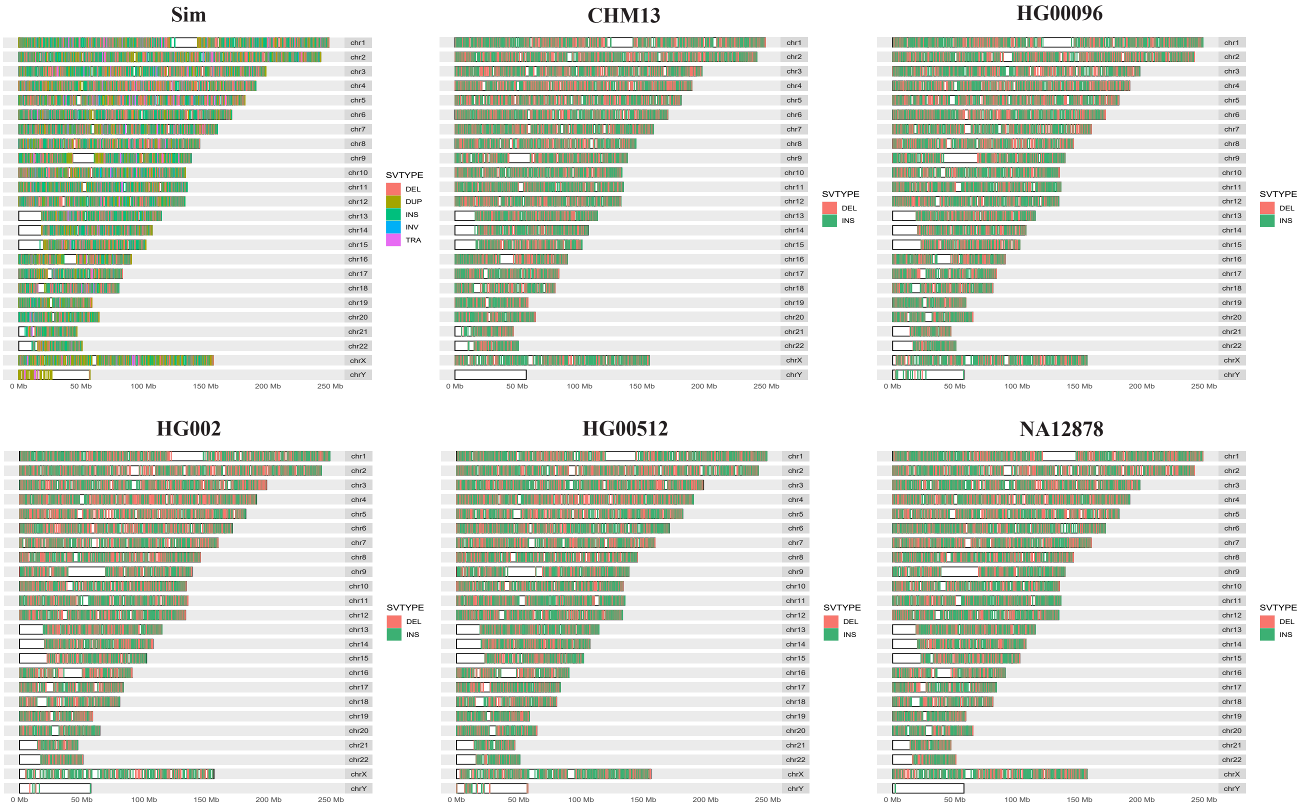


**Fig S3.** Location distribution of SV on the hg38 genome on the benchmark datasets of simulated (Sim) and real samples.

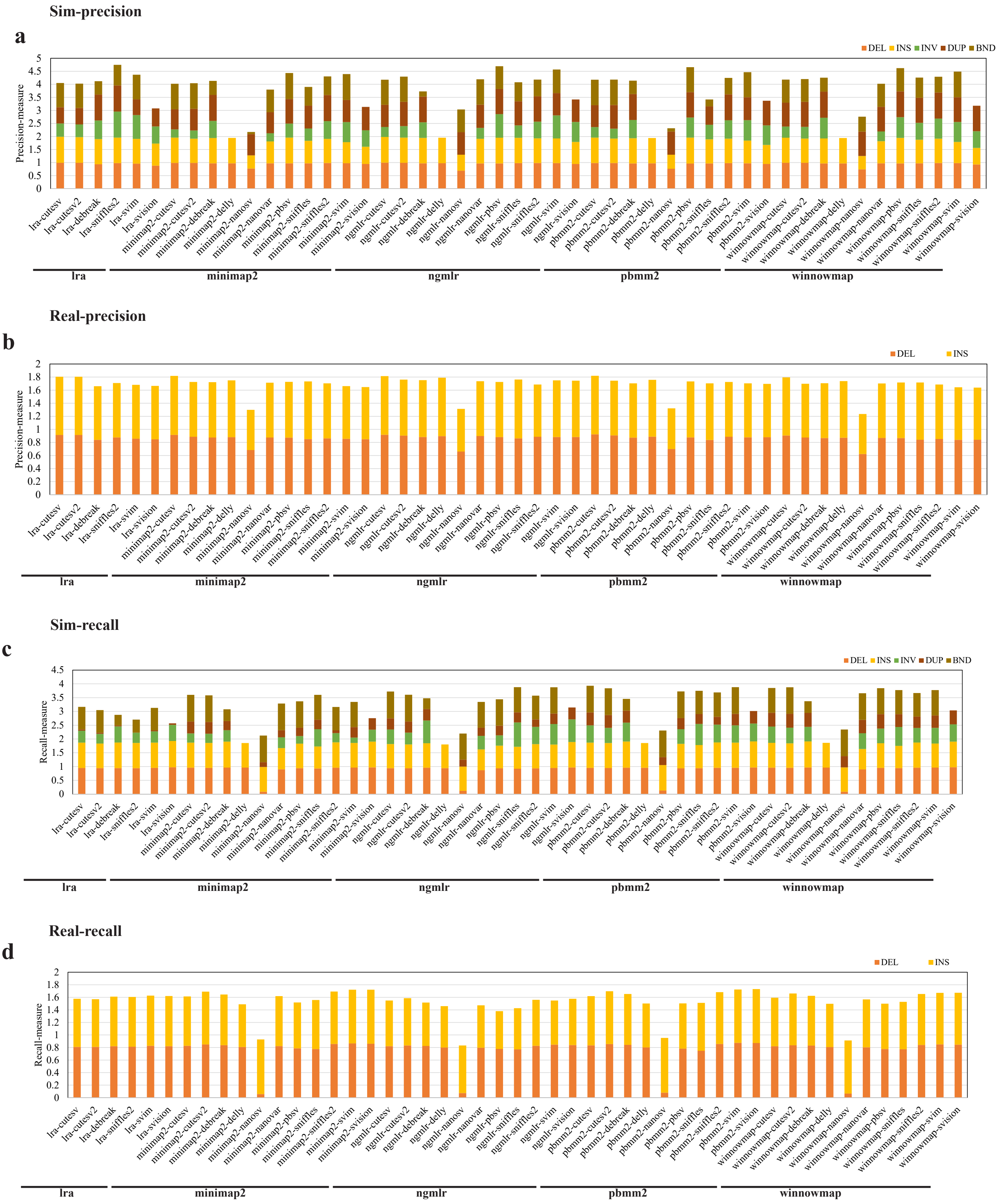


**Fig S4.** Performance of SV detection pipelines in different SV types(CSS). Precision and recall of DEL, DUP, INS, INV and BND were determined with the simulated (**a**(precision), **c**(recall)) and the real data(**b**(precision), **d**(recall)). Precision-measure and recall-measure are shown for the pipelines indicated with orange (for DEL), yellow (for INS), green (for DUP), brown (for INV) and olive(for BND)bars. Pipelines are categorized according to the alignment tools (lra, minimap2, ngmlr, pbmm2, winnowmap).

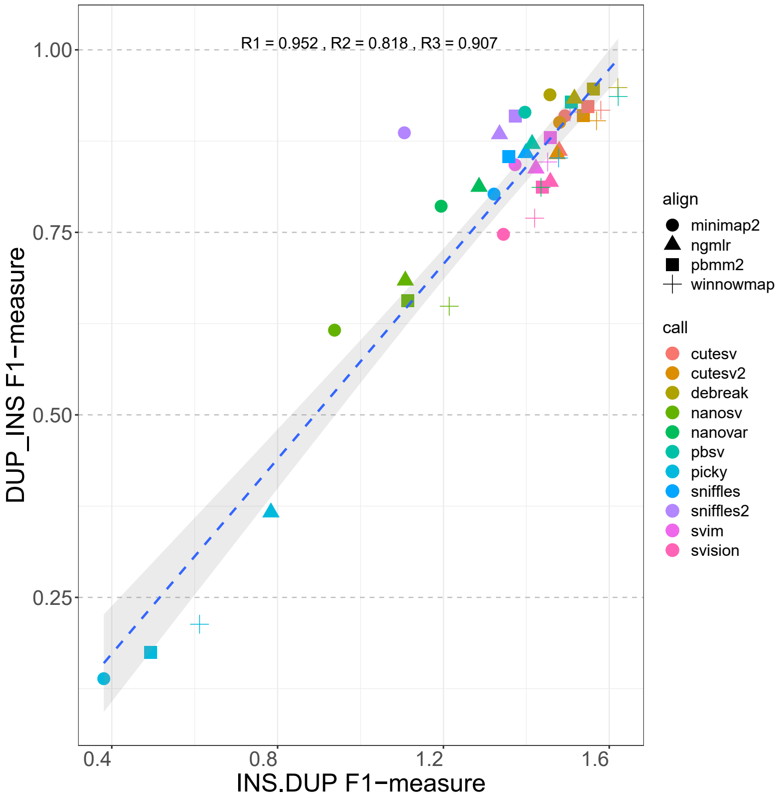


**Fig S5.** In simulated data, the correlation coefficient of F1-measure between pipelines using DUP_INS and non-DUP_INS. R1 represents the Pearson correlation coefficient, R2 represents the Spearman correlation coefficient, and R3 is the square of the Pearson correlation coefficient. The "INS.DUP" F1-measure represents the cumulative F1 scores of DEL and INS. The DUP_INS F1-measure represents the F1 scores of DUP_INS.

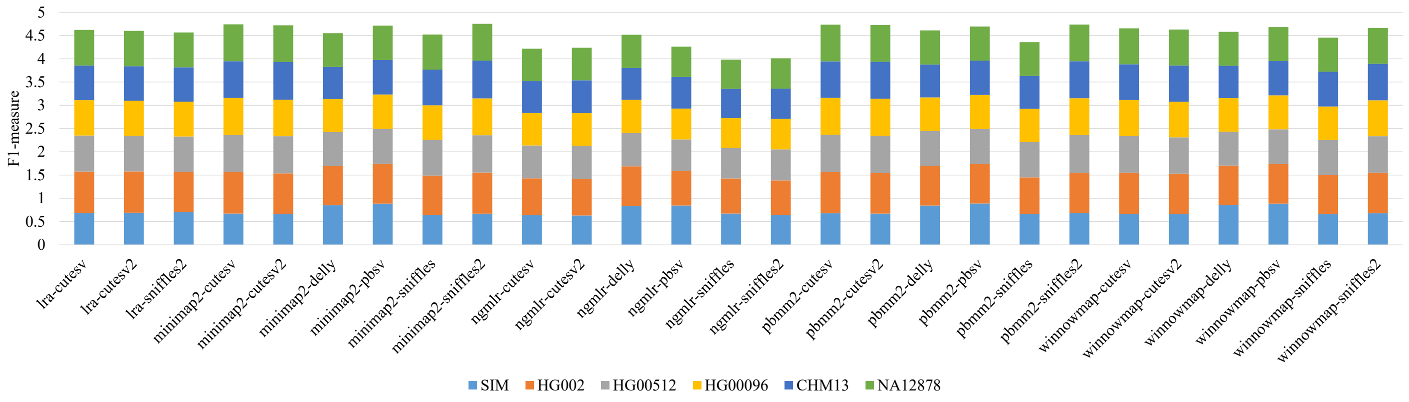


**Fig S6.** The F1 score of insertions considering sequence consistency（0.7; CCS） in simulated and real sample data. The y-axis F1-measure reflects the cumulative INS F1 score across different samples.

**a SIM**

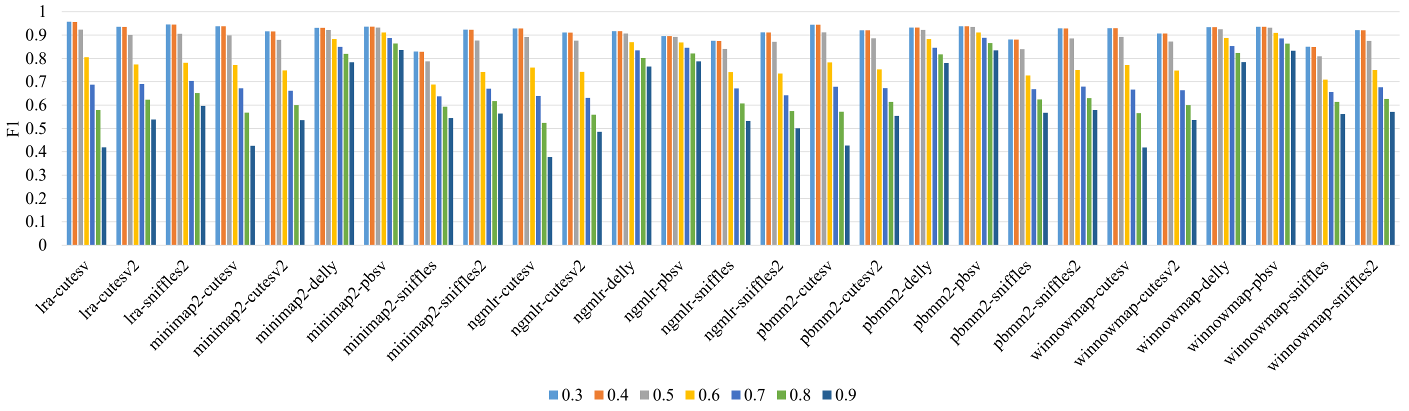


**b HG002**

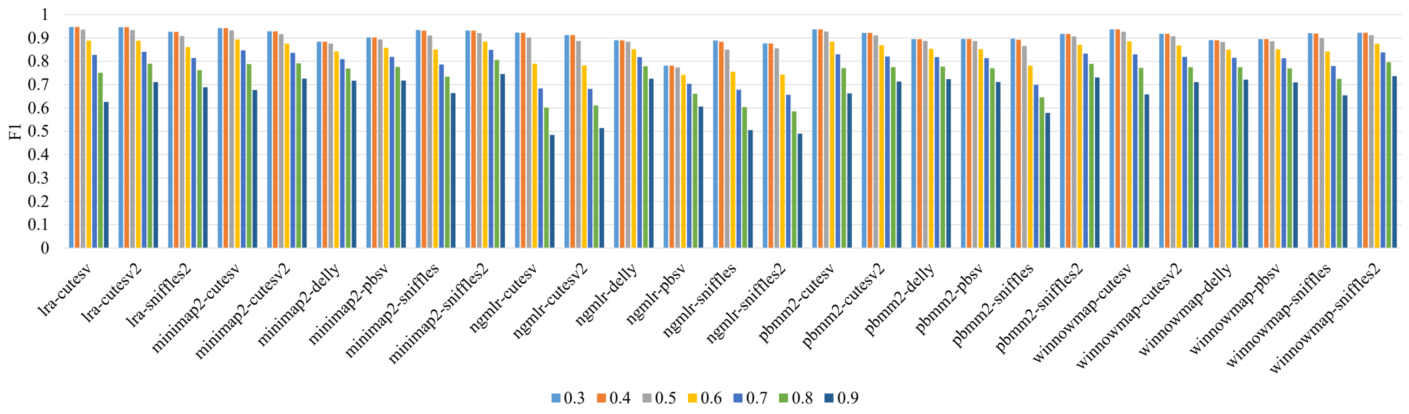


**Fig S7.** The distribution of F1 scores for samples SIM and HG002 under different insertions considering sequence consistency(0.3, 0.4, 0.5, 0.6, 0.8, 0.9).

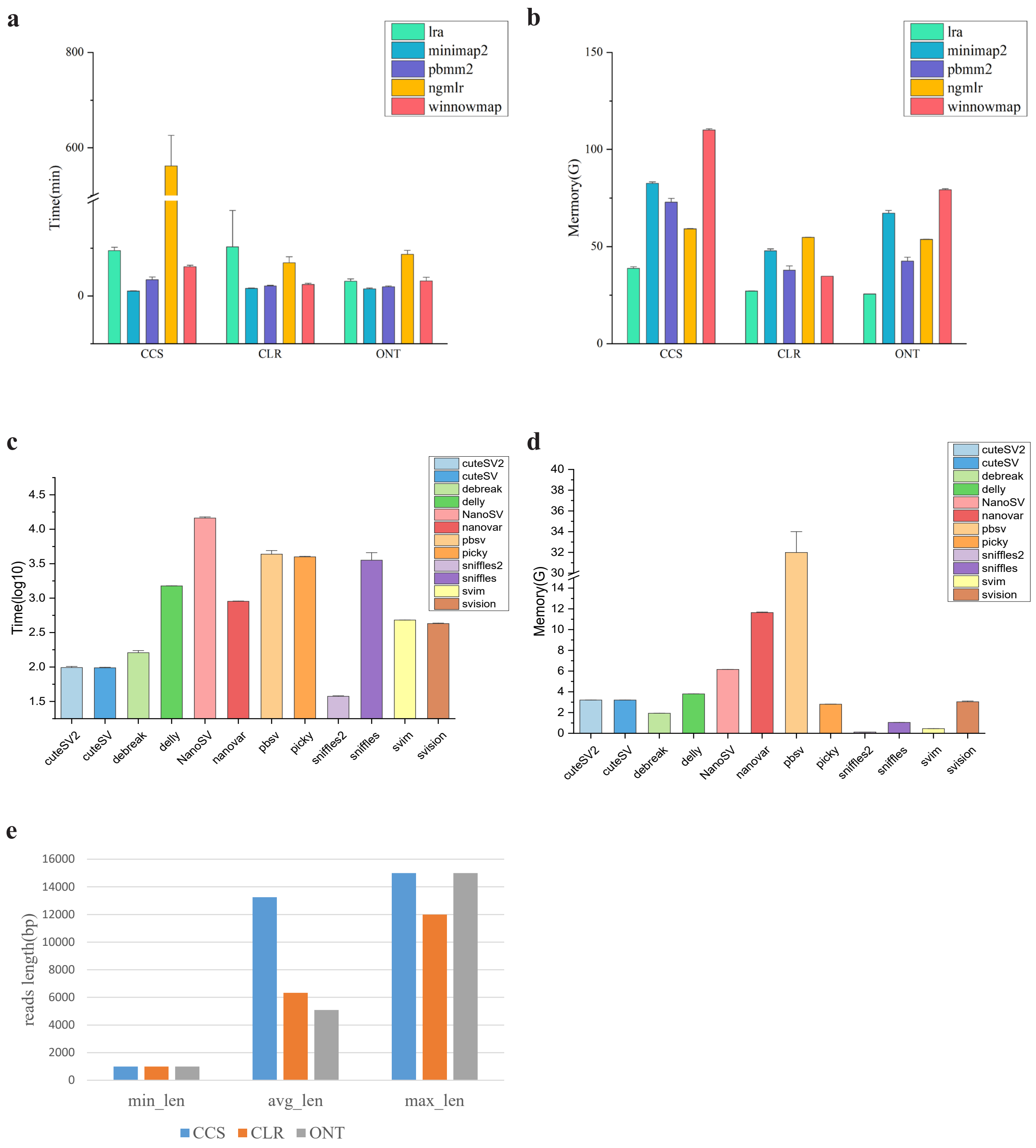


**Fig S8.** The running time and memory usage of aligners and callers in pipelines. **a**. represents the running time of aligners; **b**. represents the memory usage of aligners; **c**. represent the running time of callers; **d**. represents the memory usage of callers; **e**. represents the reads lengths of HG002(depth 5x).

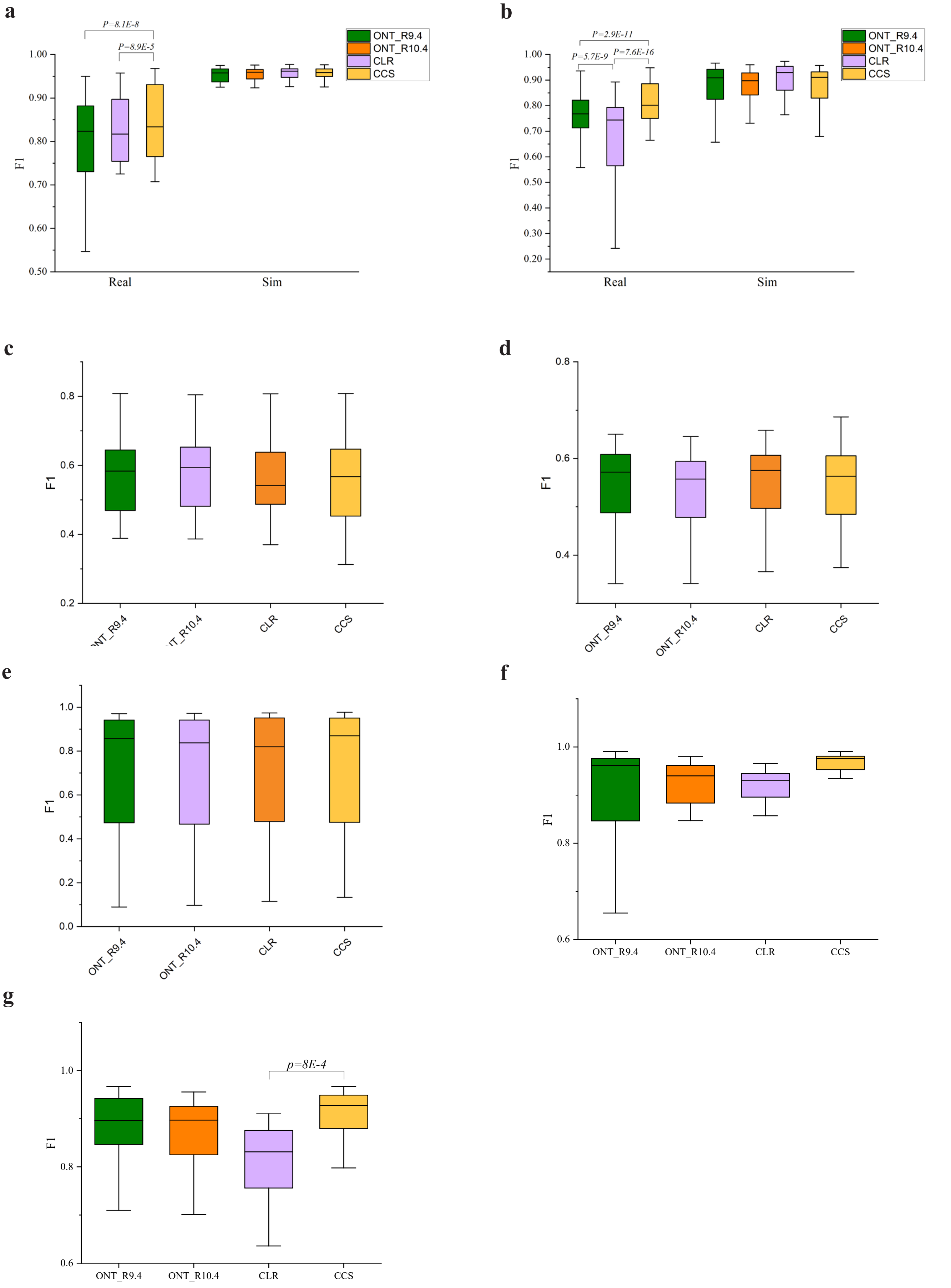


**Fig S9.** F1 Distribution of pipelines in Pacbio and Nanopore sample data under 25x sequencing depth. **a**: DEL; **b**: INS; **c**: INV; **d**: DUP; **e**: BND; **f**: HG002 chr20 DEL, **g**: HG002 chr20 INS.

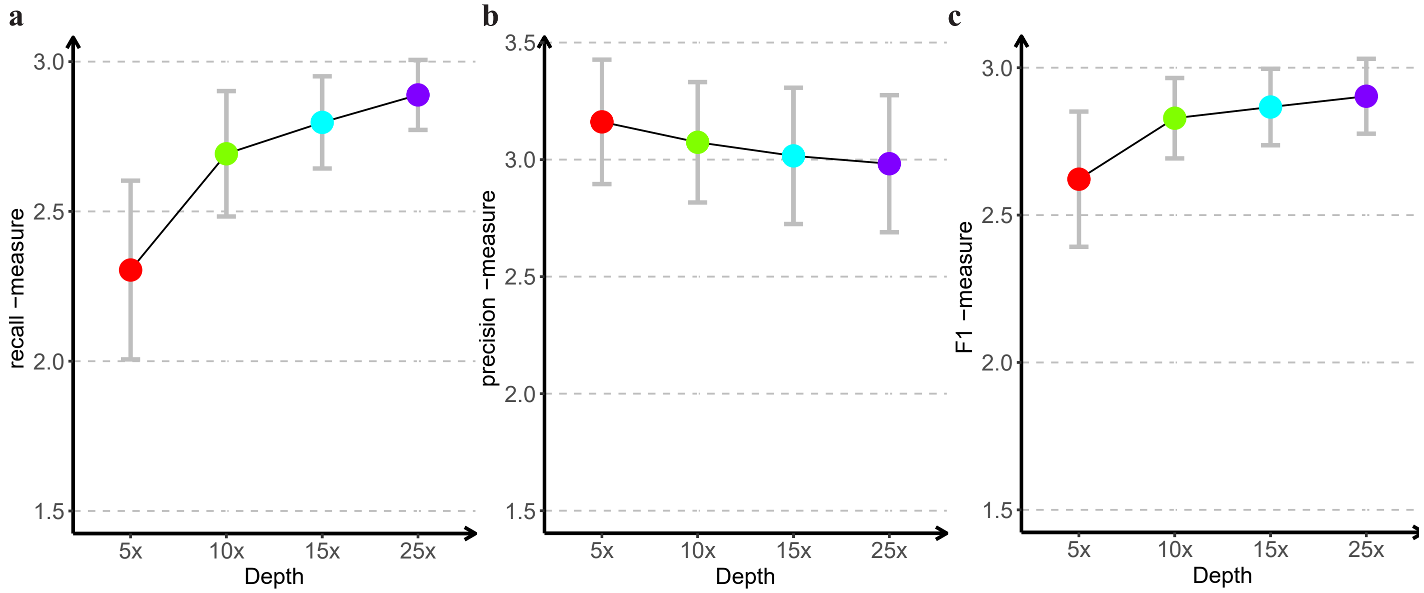


**Fig S10.** Effect of sequencing depth on recall, precision and F1 of different SV detection pipelines. Sequencing depth gradients of 5x,10x,15,25x. **a**: recall; **b**: precision; **c**: F1.

**a**

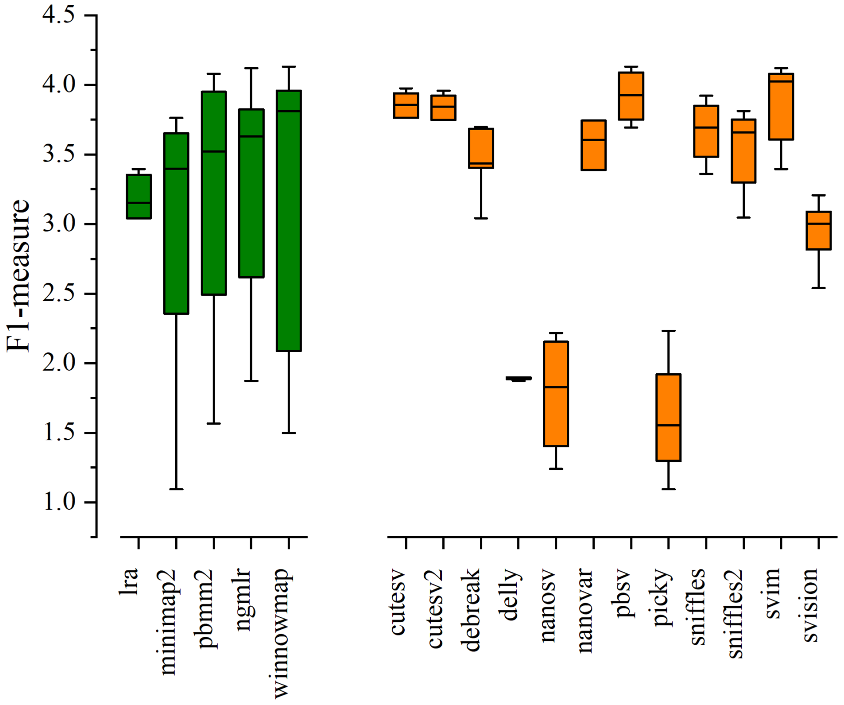


**b**

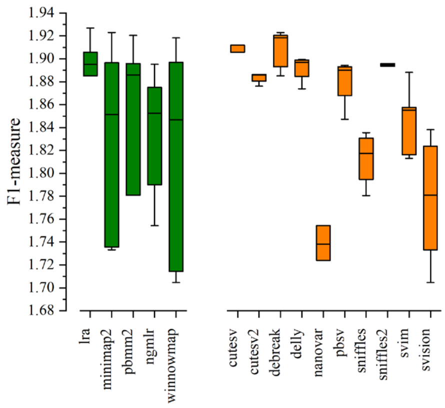


**c**

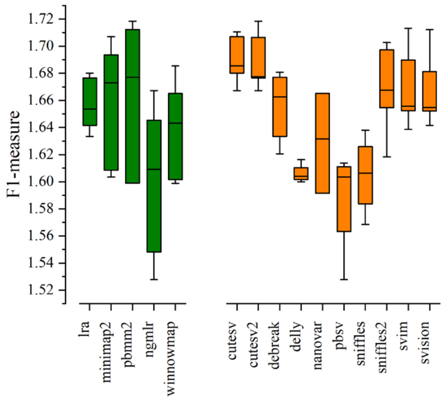


**Fig S11.** The selection of callers and aligners in pipelines and their impact on performance. **a**. The distribution of F1-measure (DEL, INS, INV, DUP, BND) for pipelines belonging to specific aligners or callers in simulated data. **b**. The distribution of F1-measure (DEL, INS) for pipelines belonging to specific aligners or callers in simulated data. **c**. The distribution of F1-measure (DEL, INS) for pipelines belonging to specific aligners or callers in real data.

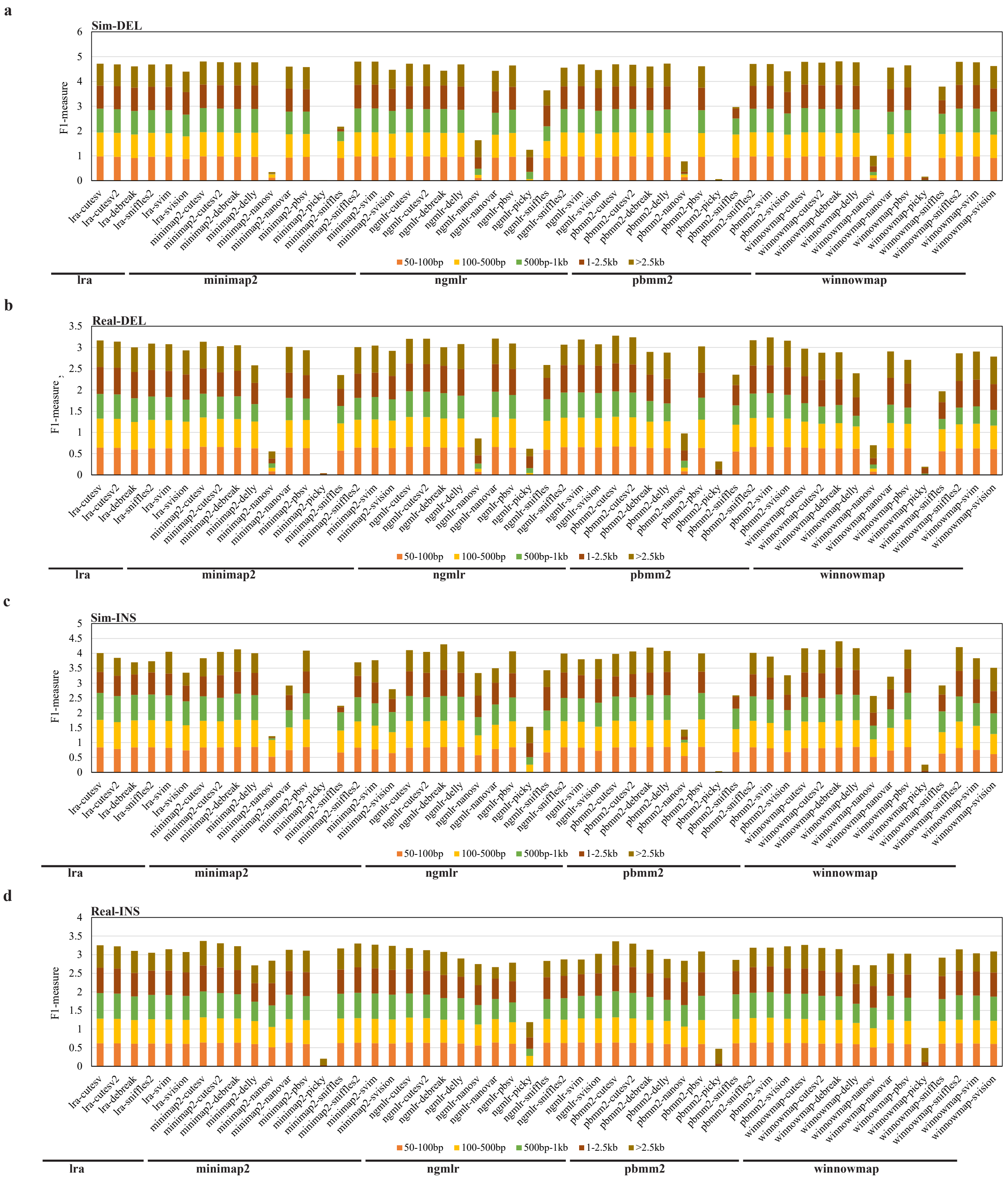


**Fig S12.** SV size specificity of SV detection pipelines(CCS). Precision and recall of different sizes SV were determined using the simulated (**a**: DEL, **c**: INS) and real data(**b**: DEL, **d**: INS). Modified F1-measures (the combined statistics for precision and recall (see the "Methods" section for details)) are shown for the pipelines indicated with orange (for 50-100bp), yellow (for 100-500bp), green (for 500-1kb), and reddish brown (for 1-2.5kb), and yellowish brown(for >2.5kb)bars. Pipelines are categorized according to the alignment tools (lra,minimap2,ngmlr,pbmm2， winnowmap).

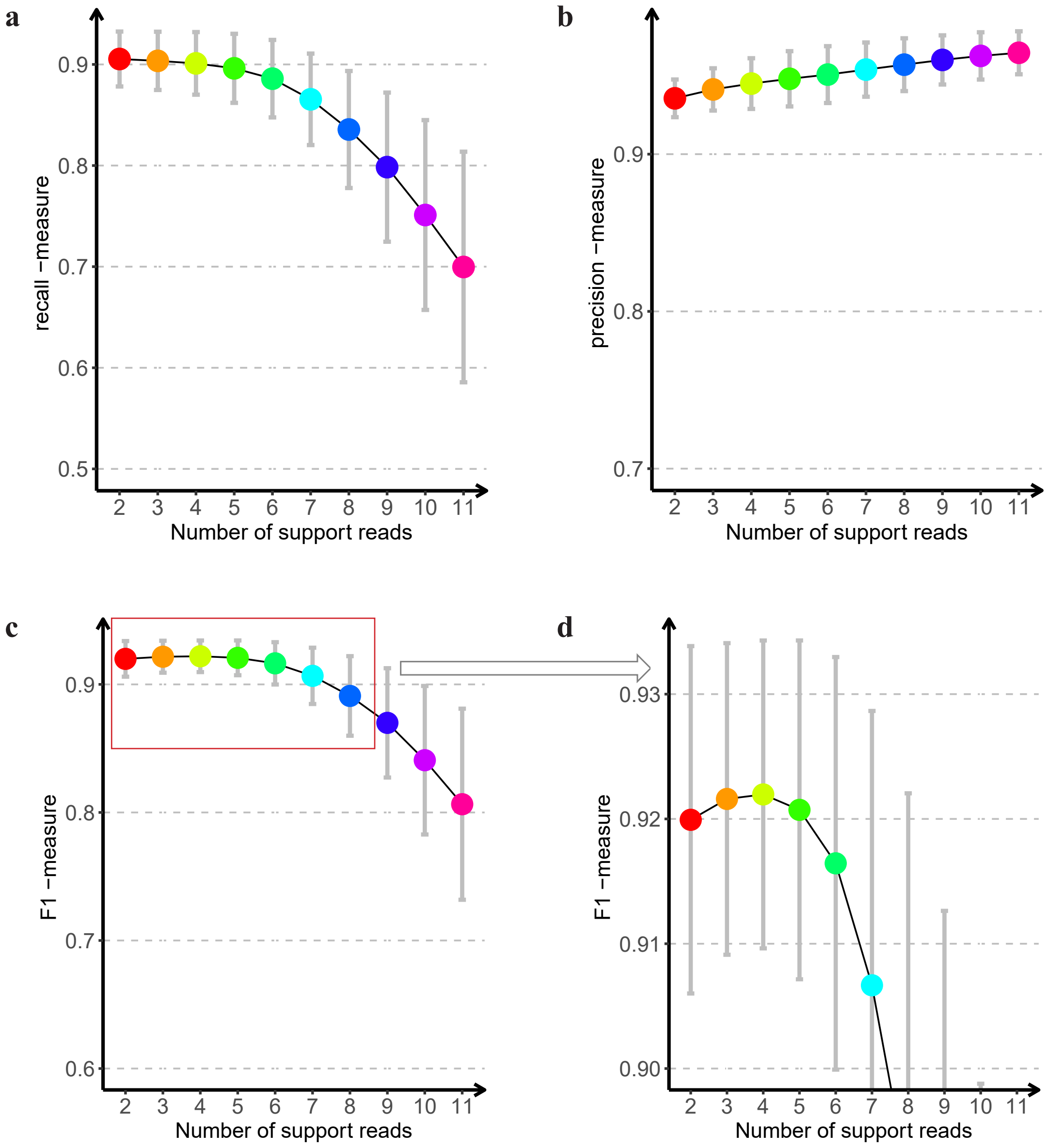


**Fig S13.** Effect of support reads on recall, precision and F1 of different SV detection pipelines. **a**: recall; **b**: precision; **c**: F1; **d**: F1 scores in detailed graphs with fewer support reads.

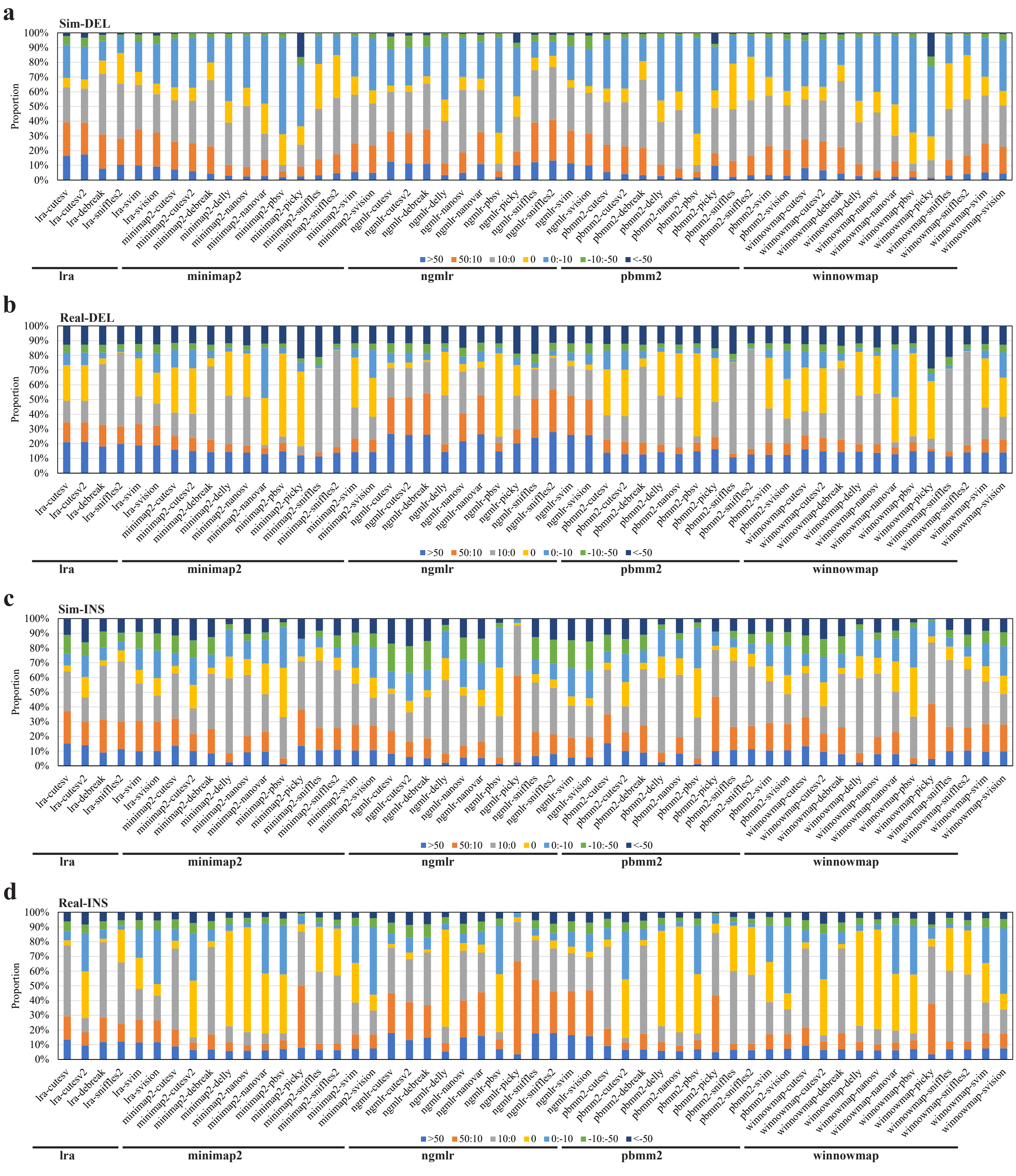


**Fig S14**. Specificity of breakpoint position deviation range in SV detection Pipelines (CCS). Breakpoint position deviations were determined by comparing the pipelines' true positives (TP) to the reference SV, using both simulated data (**a**: DEL, **c**: INS) and real data (**b**: DEL, **d**: INS). The breakpoint position deviations for pipeline TP SVs were categorized into seven groups: 0-10, 10-50, >50, 0, -10-0, -10--50, and <-50. Statistics on the breakpoint position deviations are provided (see the "Methods" section for details).

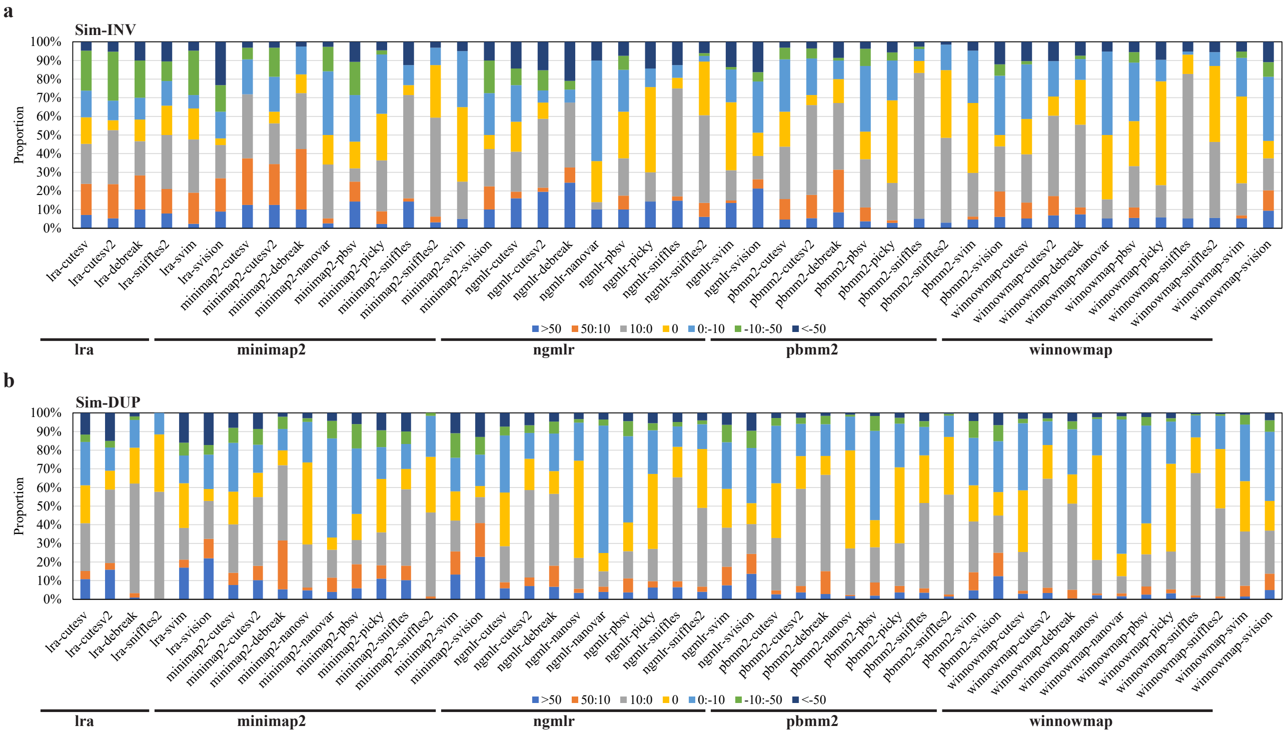


**Fig S15.** Specificity of breakpoint position deviation range in SV detection Pipelines (CCS). Breakpoint position deviation was determined with pipelines TP and reference SV difference from simulated (**a**: INV, **c**: DUP). The breakpoint position deviation of pipelines TP SV was divided into seven groups (TP SV errors: 0:10, 10:50, > 50, 0, -10:0, -10:-50, <-50), Statistics of breakpoint position deviation(see the "Methods" section for details).

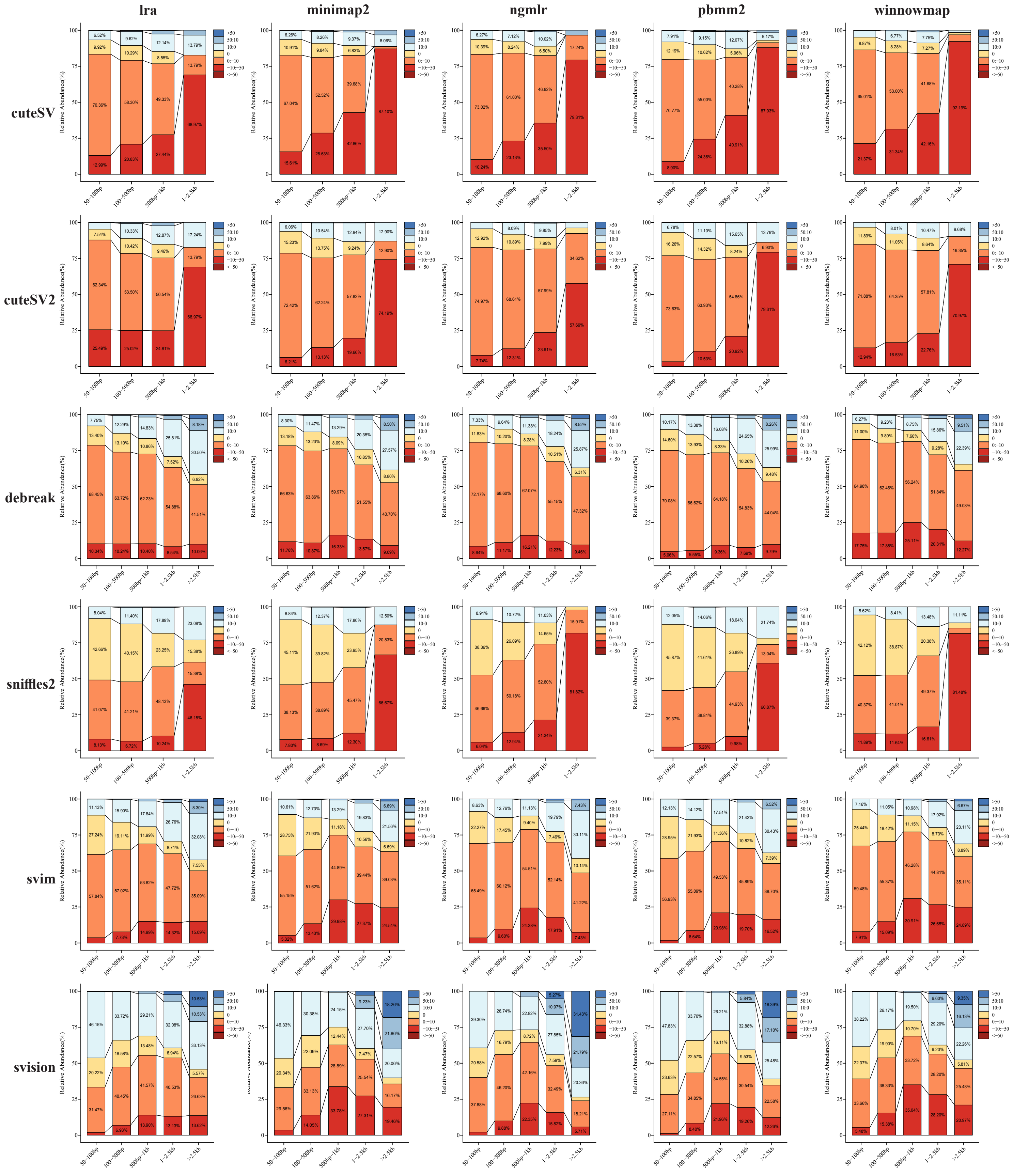


**Fig S16.** Distribution of SV length range and length deviation of INS in different pipelines(CCS SIM). The legend depicts colours ranging from deep blue to red, representing different deviation scales (>50bp, 50:10bp, 10:0bp, 0bp, 0:-10bp, -10:-50bp, <-50bp). The x-axis represents five length intervals of SV size (50-100bp, 100-500bp, 500-1kb, 1kb-2.5kb, >2.5kb). The y-axis represents the proportion of different deviation scales within the corresponding length ranges.

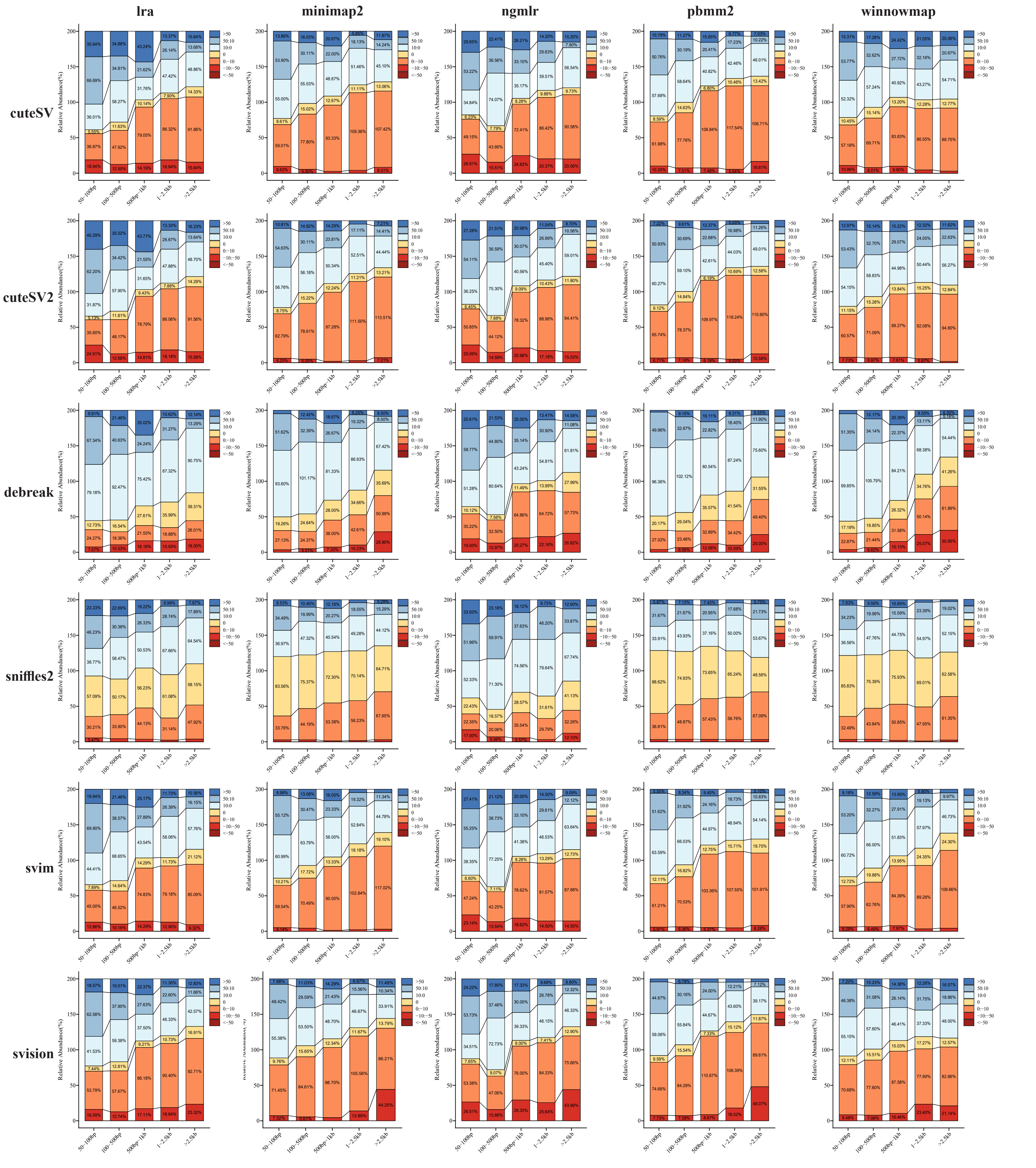


**Fig S17.** Distribution of SV size range and breakpoint deviation of DELand INS in different pipelines(CCS SIM). The legend depicts colours ranging from deep blue to red, representing different deviation scales (>50bp, 50:10bp, 10:0bp, 0bp, 0:-10bp, -10:-50bp, <-50bp). The x-axis represents five length intervals of SV size (50-100bp, 100-500bp, 500-1kb, 1kb-2.5kb, >2.5kb). The y-axis represents the proportion of different deviation scales within the corresponding length ranges.

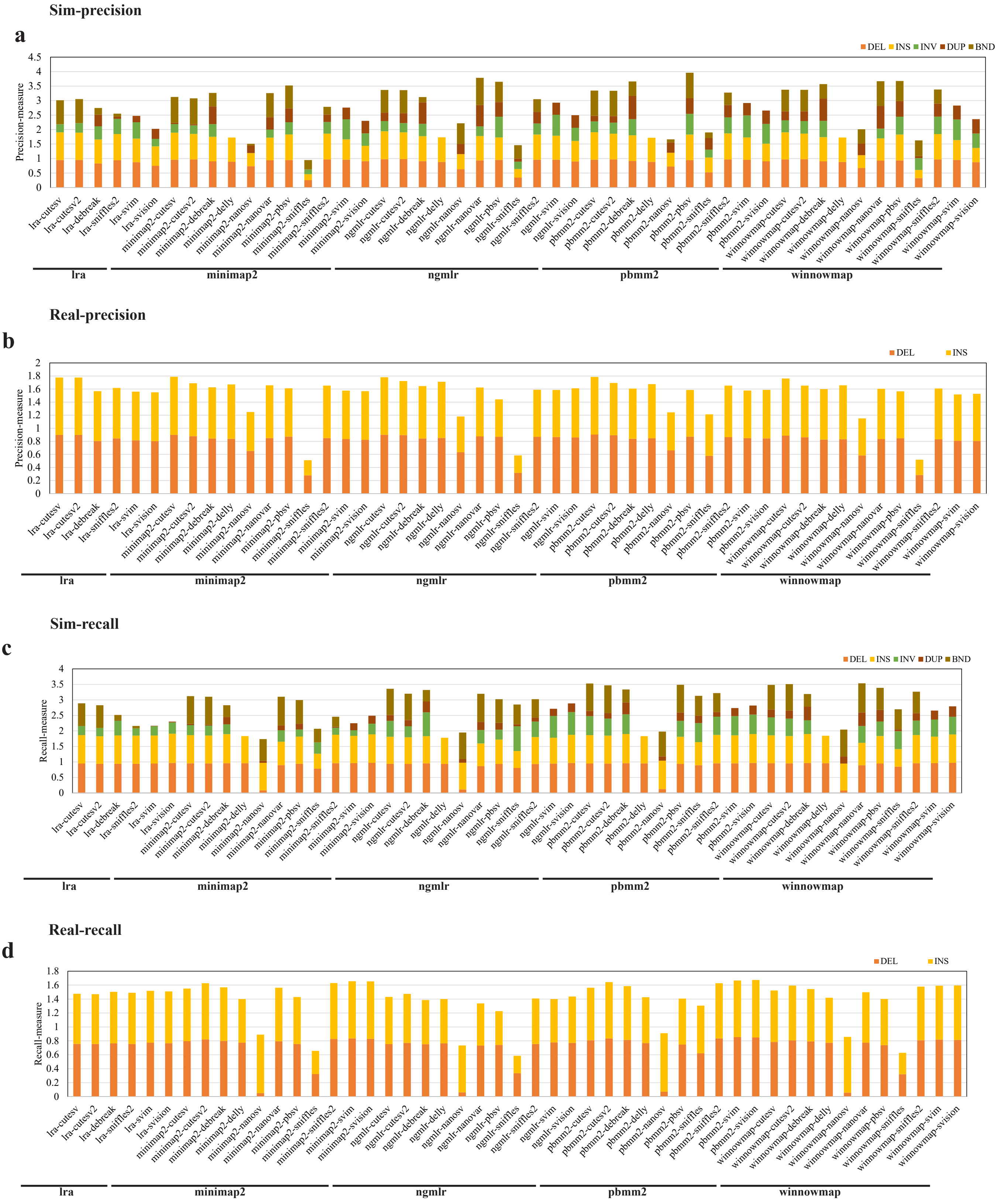


**Fig S18.** Performance of SV detection pipelines in different SV types(CCS GT). Precision and recall of DEL, DUP, INS, INV and BND were determined with the simulated (**a**(precision), **c**(recall)) and the real data(**b**(precision), **d**(recall)). Precision-measure and recall-measure are shown for the pipelines indicated with orange (for DEL), yellow (for INS), green (for DUP), brown (for INV) and olive(for BND)bars. Pipelines are categorized according to the alignment tools (lra, minimap2, ngmlr, pbmm2, winnowmap).

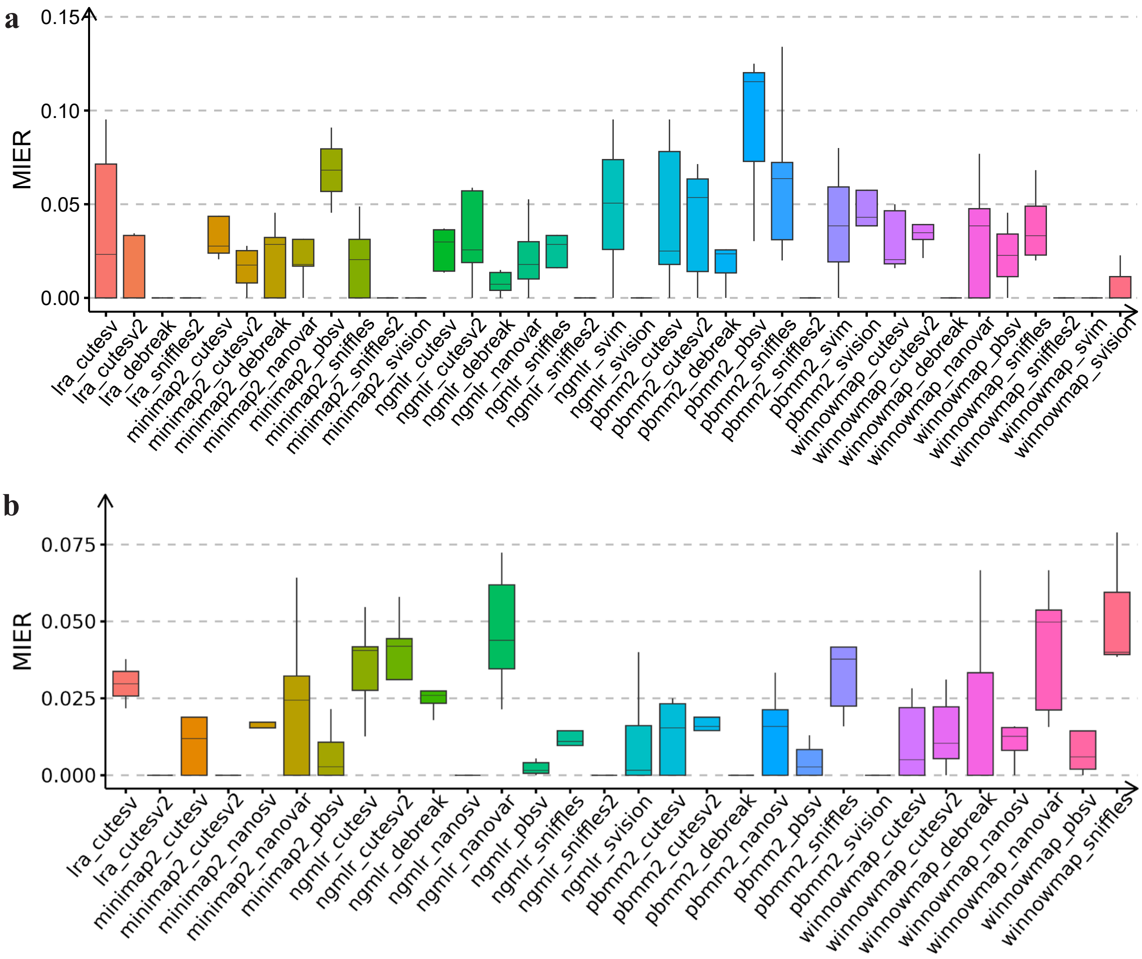


**Fig S19.** SV MIER(Mendelian error rate) of SV detection pipelines in Pedigree based on the hg38 genome. SV detection Pipelines MIER was determined with pedigree data(ONT, CCS,CLR: (HG002, HG003, HG004); CCS: (HG005, HG006, HG007)) in different SV types (INV(**a**), DUP(**b**)). Statistics MIER(see the "Methods" section for details).

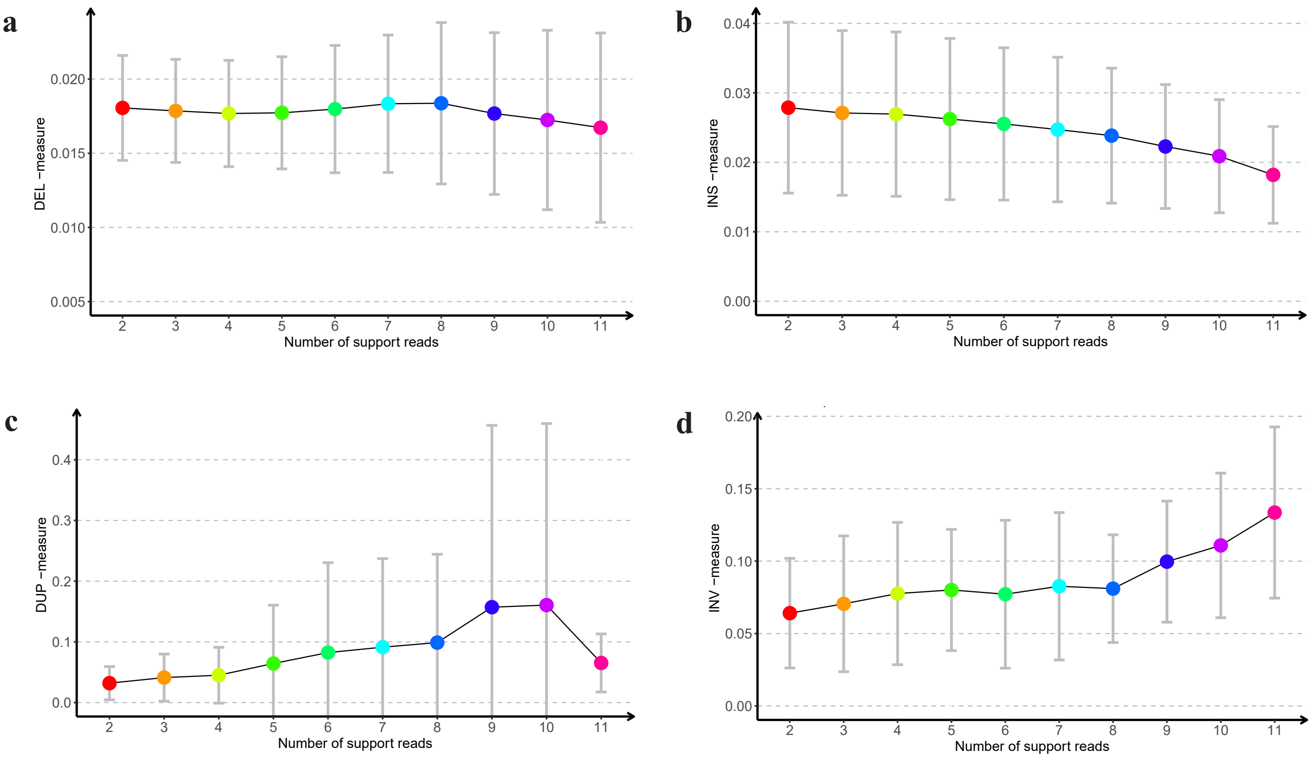


**Fig S20.** Effect of SV "minimum support reads number" on MIER of different SV detection pipelines by DEL(**a**), INS(**b**), DUP(**c**), INV(**d**) (sequence depth: 25x). SV support reads of 2-11.

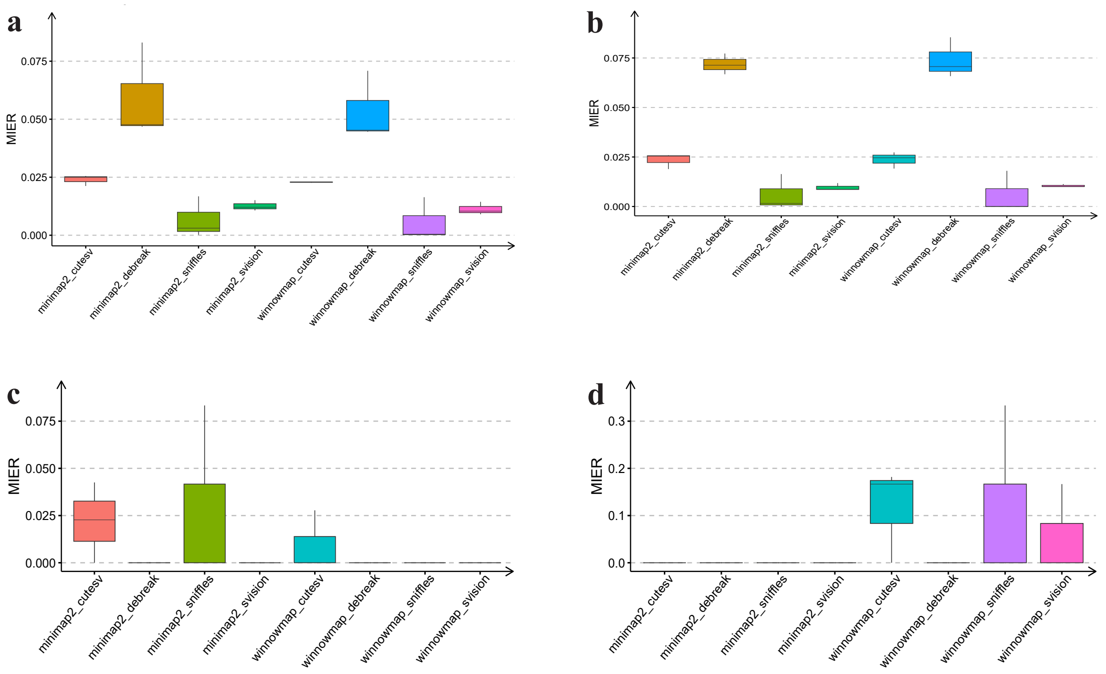


**Fig S21.** SV MIER(Mendelian error rate) of SV detection pipelines in Pedigree based on T2T genome. SV detection Pipelines MIER was determined with pedigree data(Nanopore, Pacbio: (HG002,HG003,HG004); Pacbio: (HG005,HG006,HG007)) in different SV types (DEL(**a**), INS(**b**), DUP(**c**), INV(**d**)). Statistics MIER(see the "Methods" section for details).

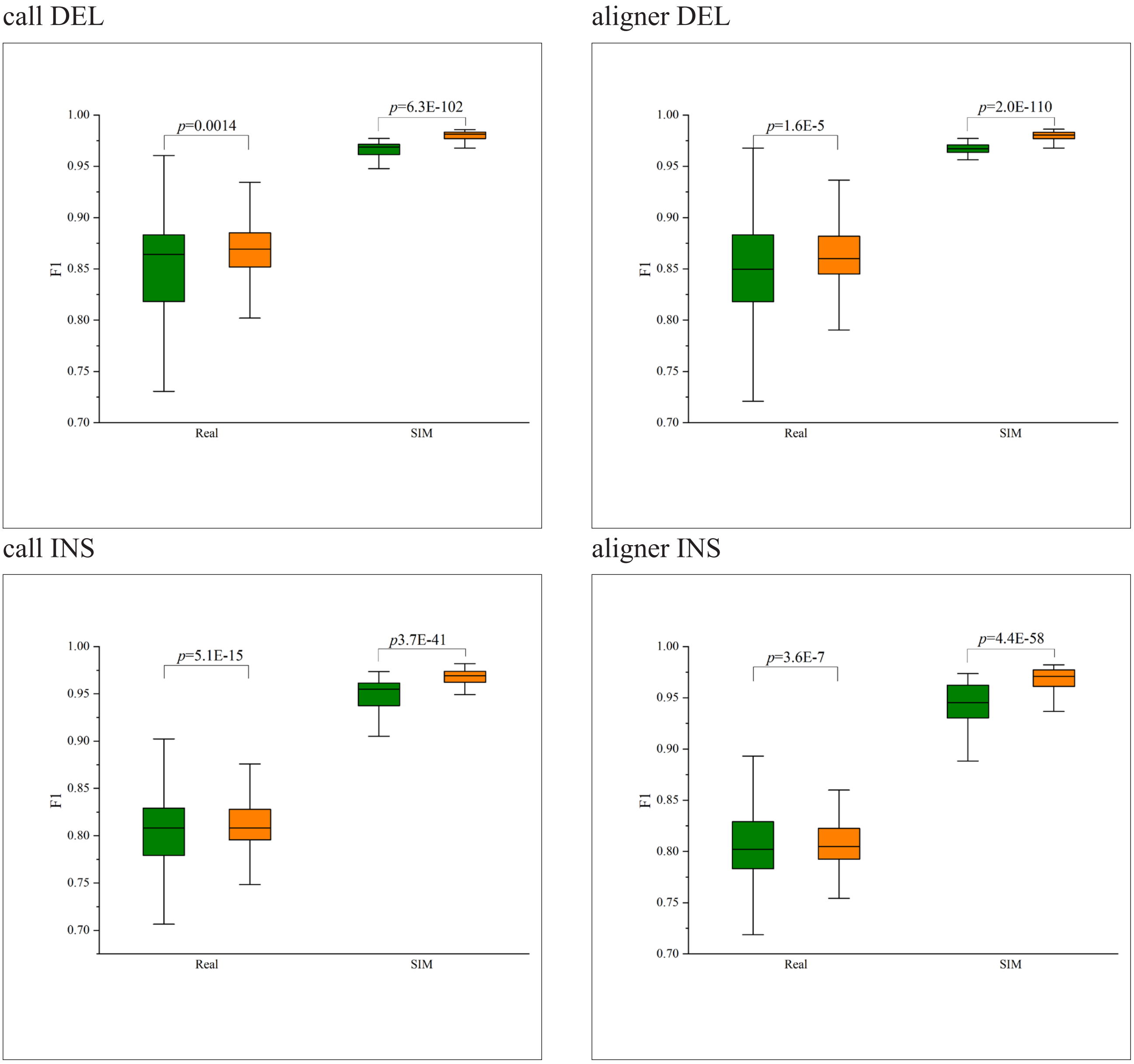


**Fig S22.** The distribution of F1 scores between simulated and real data under pipelines combining strategies based on caller and aligner.

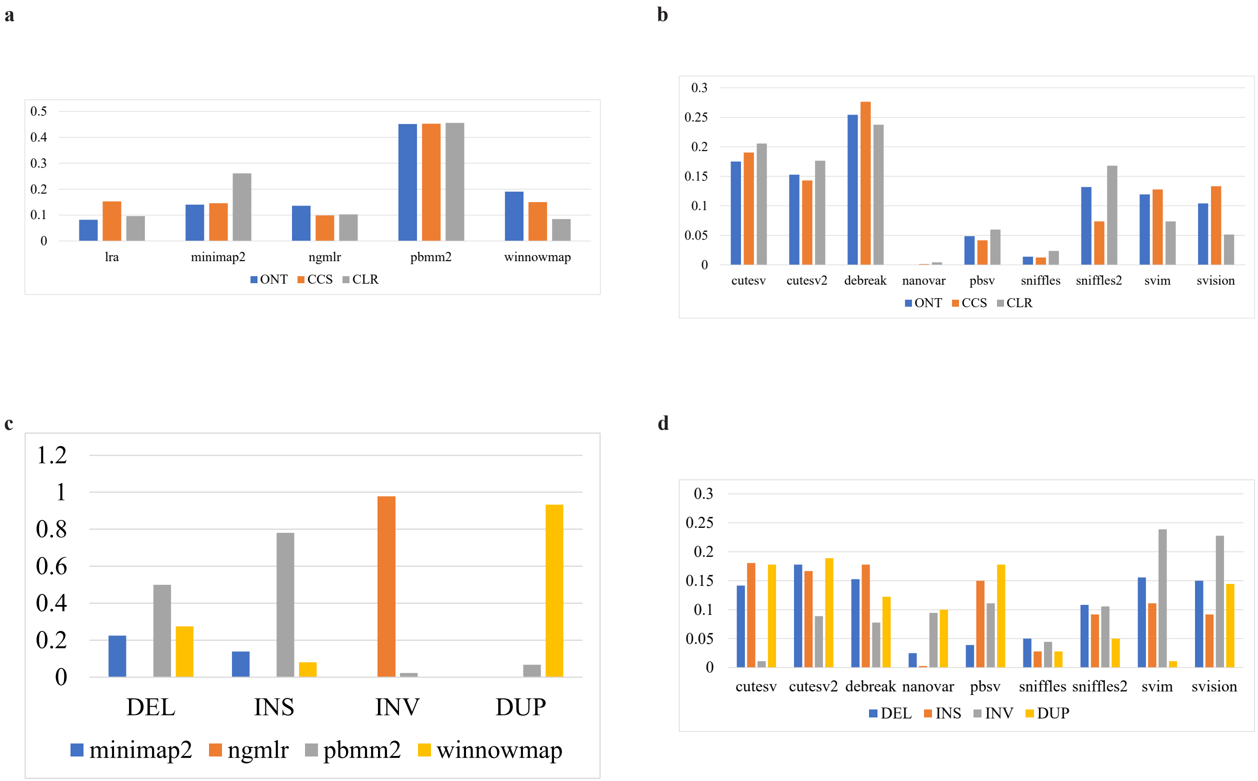


**Fig S23.** The frequency of aligners and callers in the top 10 of Two-pipelines and Three-pipelines. **a-b**: The top 10 multi-pipelines ranked by F1-measure.Calculate the frequency of callers and aligners in the top 10 median pipelines for each data type (ONT, CLR, CCS) in both simulated and real datasets. **c-d**: The top 10 multi-pipelines ranked by F1-measure. of SV types. Calculate the frequency of callers and aligners in the top 10 median pipelines for different SV types in both simulated and real datasets. The frequencies of callers and aligners in the top 10 pipelines from simulated and real datasets are combined for statistical analysis.

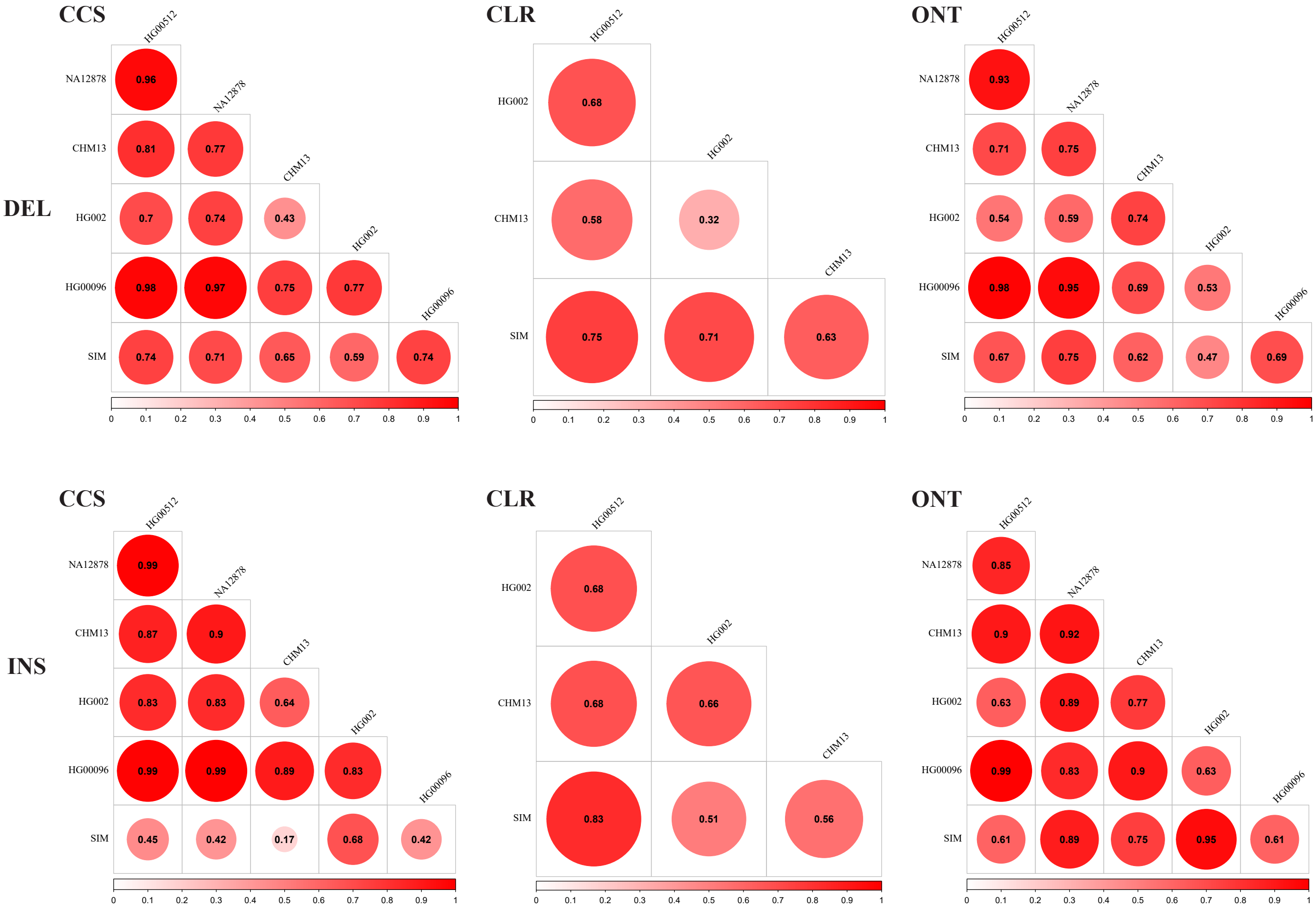


**Fig S24.** Pipelines on simulation(SIM) and real data(HG002,HG00512,HG00096,CHM13,NA12878) for SV detection performance F1 in inter-sample "spearman" correlation.
