# Supplementary material for: Comprehensive and deep evaluation of structural variation detection pipelines with third-generation sequencing data
Source: Genome Biol. 2024 Jul 15;25:188. doi: 10.1186/s13059-024-03324-5 (PMC11247875; doi:10.1186/s13059-024-03324-5)
Supplement: Supplementary file 2 — Additional file 2: Tables S1–S3. It contains versions of aligners and callers (Table S1), the number of SV benchmarks in simulated and real data (Table S2), and the read length of dataset samples (Table S3). [file 13059_2024_3324_MOESM2_ESM.docx]

**Table S1(aligner and caller):**

**Aligners:**

| Name | Time | Version | Cited times(2024.1.29 google Scholar) |
| --- | --- | --- | --- |
| lordfast | 2019 | 0.0.10 | 22 |
| lra | 2021 | 1.3.7.1 | 58 |
| minimap2 | 2018 | 2.17-r941 | 7416 |
| ngmlr | 2018 | 0.2.7 | 1190 |
| pbmm2 | 2018 | 1.3.0 | 494 |
| winnowmap | 2020 | 1.11 | 142 |

**Callers:**

| Name | Time | Version | Cited times(2024.1.29 google Scholar) |
| --- | --- | --- | --- |
| cuteSV | 2020 | 1.0.8 | 172 |
| cuteSV2 | 2022 | 2.0.3 | 3 |
| DeBreak | 2022 | 1.0.2 | 9 |
| DELLY | 2012 | 0.8.1 | 1927 |
| NanoVar | 2020 | 1.3.2 | 85 |
| NanoSV | 2017 | 1.2.4 | 345 |
| pbsv | 2017 | 2.3.0 | 641 |
| Picky | 2018 | 0.2.a | 90 |
| Sniffles | 2018 | 1.0.12 | 1190 |
| Sniffles2 | 2022 | 2.2 | 62 |
| SVIM | 2019 | 1.4.2 | 215 |
| SVision | 2022 | 1.3.8 | 25 |

**Alignment tools * Call SV tools = 6*12 = 72**

**Invalid pipelines:** pbmm2-Nanovar, lra-Sniffles, lra-Picky, lra-delly, lra-NanoVar, lra-NanoSV, lra-pbsv

**Filtered pipelines:** lordfast-related pipelines

**The final pipelines: 72-1-6-12=53**

**Table S2( The number of SV benchmarks of simulated and real data):**

| sample | DEL | DUP | INS | INV | BND |
| --- | --- | --- | --- | --- | --- |
| SIM | 7214 | 2919 | 9989 | 51 | 368 |
| CHM13 | 7622 | - | 12448 | - | - |
| HG00096 | 6151 | - | 10091 | - | - |
| HG00512 | 6135 | - | 10118 | - | - |
| NA12878 | 6115 | - | 9956 | - | - |

**Table S3(The read length conditions of dataset samples)：**

| SampleName | Platform | Depth | Reads mean length(bp) | Reads max length(bp) |
| --- | --- | --- | --- | --- |
| CHM13 | CCS | 25x | 18028 | 47637 |
|  | CLR |  | 12762.3 | 103213 |
|  | ONT |  | 14188.9 | 249985 |
| NA12878 | CCS |  | 9963.3 | 21348 |
|  | ONT |  | 8484.3 | 1433139 |
| HG002 | CCS |  | 13478.4 | 21797 |
|  | CLR |  | 8322.9 | 85977 |
|  | ONT |  | 8097.1 | 883235 |
| HG003 | CCS |  | 15413.4 | 32234 |
|  | CLR |  | 8,078.2 | 78046 |
|  | ONT |  | 11379.3 | 741514 |
| HG004 | CCS |  | 16032.3 | 32661 |
|  | CLR |  | 8039.6 | 77085 |
|  | ONT |  | 19769.5 | 566634 |
| HG005 | CCS |  | 10423.6 | 21085 |
|  | CLR |  | 9651.2 | 150257 |
| HG00512 | CCS |  | 15389.4 | 39474 |
|  | CLR |  | 25142.8 | 319140 |
|  | ONT |  | 56546.3 | 1180079 |
| HG006 | CCS |  | 16313.6 | 32872 |
|  | CLR |  | 9895.1 | 126040 |
| HG007 | CCS |  | 17553.8 | 32797 |
|  | CLR |  | 10248.7 | 109461 |
| HG00096 | CCS |  | 14962.2 | 67892 |
|  | ONT |  | 48954.2 | 1152381 |
| Sim_DEL_INS_INV_DUP | CCS |  | 15005.4 | 198326 |
|  | CLR |  | 14934.2 | 172021 |
|  | ONT_R9.4 |  | 14884.1 | 210815 |
|  | ONT_R10.4 |  | 14958.2 | 193157 |
| Sim_TRA | CCS |  | 15004.9 | 193610 |
|  | CLR |  | 14997.1 | 197490 |
|  | ONT_R9.4 |  | 14888.1 | 179974 |
|  | ONT_R10.4 |  | 14973.1 | 185516 |
| HG002_chr20 | ONT_R9.4 |  | 18793.2 | 900827 |
|  | ONT_R10.4 |  | 12398.2 | 151196 |
