# Supplementary material for: Comprehensive and deep evaluation of structural variation detection pipelines with third-generation sequencing data
Source: Genome Biol. 2024 Jul 15;25:188. doi: 10.1186/s13059-024-03324-5 (PMC11247875; doi:10.1186/s13059-024-03324-5)
Supplement: Supplementary file 5 — Additional file 5. Detailed parameters for generating simulated TGS reads and constructing pipelines. [file 13059_2024_3324_MOESM5_ESM.docx]

**Create simulation data**

1. VISOR HACk -g hg38.fa -b sim_DEL.DUP.INS.INV_h1.bed sim_DEL.DUP.INS.INV_h2.bed -o VISOR_HACk_Sim_DEL.DUP.INS.INV
2. VISOR HACk -g hg38.fa -b sim_TRA.bed -o VISOR_HACk_Sim_TRA
3. echo -e “pacbio2016\npacbio2021\nnanopore2020\nnanopore2023”|while read id;do(VISOR LASeR -g -s VISOR_HACk_Sim_DEL.DUP.INS.INV -b LASeR100.bed -o $id\_VISOR_LASeR_Sim_DEL.DUP.INS.INV --coverage 25 --threads 80 --tag --read_type nanopore --error_model $id --qscore_model $id );done
4. echo -e “pacbio2016\npacbio2021\nnanopore2020\nnanopore2023”|while read id;do(VISOR LASeR -g -s VISOR_HACk_Sim_TRA -b LASeR100.bed -o $id\_VISOR_LASeR_Sim_ TRA --coverage 25 --threads 80 --tag --read_type nanopore --error_model $id --qscore_model $id );done

**Aligners:**

# lordfast alignment:

1. **(ONT, CLR, CCS):** lordfast --search GenomeIndexPath/Genome.fa --seq sample.fq -t ThreadNumber > bamPath/sampleId.sam
2. samtools view -@ 20 -bS bamPath/sampleId.sam -o bamPath/sampleId.bam
3. bamaddrg -r sampleId -s sampleId -b bamPath/sampleId.bam >bamPath/sampleId.rg.bam
4. samtools sort -@ 5 bamPath/sampleId.rg.bam -o bamPath/sampleId.sort.bam
5. samtools index bamPath/sampleId.sort.bam

# lra alignment:

1. **ONT:** catfishq -r sample.fq |seqtk seq -A -|lra align -ONT -t 20 GenomeIndexPath/Genome.fa - -p s --printMD -SkipH --noMismatch | samtools addreplacerg -r "@RG\tID: sampleId \tSM: sampleId" - | samtools sort -@ 10 -T sampleId -O BAM -o bamPath/sampleId.sort.bam –
2. **CCS:** catfishq -r sample.fq |seqtk seq -A -|lra align -CCS -t 20 GenomeIndexPath/Genome.fa - -p s --printMD -SkipH --noMismatch | samtools addreplacerg -r "@RG\tID: sampleId \tSM: sampleId" - | samtools sort -@ 10 -T sampleId -O BAM -o bamPath/sampleId.sort.bam -
3. **CLR:** catfishq -r sample.fq |seqtk seq -A -|lra align -CLR -t 20 GenomeIndexPath/Genome.fa - -p s --printMD -SkipH --noMismatch | samtools addreplacerg -r "@RG\tID: sampleId \tSM: sampleId" - | samtools sort -@ 10 -T sampleId -O BAM -o bamPath/sampleId.sort.bam -
4. samtools index bamPath/sampleId.sort.bam

# minimap2 alignment:

1. **ONT:** minimap2 GenomeIndexPath/Genome.fa sample.fq -a -x map-ont --MD -Y -o bamPath/sampleId.sam -R '@RG\tID:sampleId\tSM:sampleId' -t ThreadNumber **CCS:** minimap2 GenomeIndexPath/Genome.fa sample.fq -a -x map-hifi --MD -Y -o bamPath/sampleId.sam -R '@RG\tID:sampleId\tSM:sampleId' -t ThreadNumber
2. **CCS:** minimap2 GenomeIndexPath/Genome.fa sample.fq -a -x map-hifi --MD -Y -o bamPath/sampleId.sam -R '@RG\tID:sampleId\tSM:sampleId' -t ThreadNumber
3. **CLR:** minimap2 GenomeIndexPath/Genome.fa sample.fq -a -x map-pb --MD -Y -o bamPath/sampleId.sam -R '@RG\tID:sampleId\tSM:sampleId' -t ThreadNumber
4. samtools view -@ 20 -bS bamPath/sampleId.sam -o bamPath/sampleId.bam
5. samtools sort -@ 5 bamPath/sampleId.bam -o bamPath/sampleId.sort.bam
6. samtools index bamPath/sampleId.sort.bam

# ngmlr alignment:

1. **ONT:** ngmlr -t ThreadNumber --rg-id sampleId --rg-sm sampleId --rg-pl Nanopore -x ont  -r GenomeIndexPath/Genome.fa -q sample.fq -o bamPath/sampleId.sam
2. **CCS, CLR:** ngmlr -t ThreadNumber --rg-id sampleId --rg-sm sampleId --rg-pl Pacbio -x pacbio -q sample.fq GenomeIndexPath/Genome.fa  -o bamPath/sampleId.sam
3. samtools view -@ 20 -bS bamPath/sampleId.sam -o bamPath/sampleId.bam
4. bamaddrg -r sampleId -s sampleId -b bamPath/sampleId.bam >bamPath/sampleId.rg.bam
5. samtools sort -@ 5 bamPath/sampleId.rg.bam -o bamPath/sampleId.sort.bam
6. samtools index bamPath/sampleId.sort.bam

# pbmm2 alignment:

1. **ONT,CCS:** pbmm2 align GenomeIndexPath/Genome.fa sample.fq bamPath/sampleId.bam --preset CCS --rg '@RG\tID:sampleId\tSM:sampleId'
2. **CLR:** pbmm2 align GenomeIndexPath/Genome.fa sample.fq bamPath/sampleId.bam --preset SUBREAD --rg '@RG\tID:sampleId\tSM:sampleId'
3. bamaddrg -r sampleId -s sampleId -b bamPath/sampleId.bam >bamPath/sampleId.rg.bam
4. samtools calmd -S --threads 20 --output-fmt BAM --reference GenomeIndexPath/Genome.fa bamPath/sampleId.rg.bam >bamPath/sampleId.rg.MD.bam
5. samtools sort -@ 20 -o bamPath/sampleId.sort.bam bamPath/sampleId.rg.MD.bam
6. samtools index bamPath/sampleId.sort.bam

# winnowmap alignment:

1. **ONT:** winnowmap -Y --MD -x map-ont GenomeIndexPath/Genome.fa sample.fq -a -o bamPath/sampleId.sam -R '@RG\tID:sampleId\tSM:sampleId'
2. **CCS:** winnowmap -Y --MD -x map-pb GenomeIndexPath/Genome.fa sample.fq -a -o bamPath/sampleId.sam -R '@RG\tID:sampleId\tSM:sampleId'
3. **CLR:** winnowmap -Y --MD -x map-pb-clr GenomeIndexPath/Genome.fa sample.fq -a -o bamPath/sampleId.sam -R '@RG\tID:sampleId\tSM:sampleId'
4. samtools view -@ 20 -bS bamPath/sampleId.sam -o bamPath/sampleId.bam
5. samtools sort -@ 5 bamPath/sampleId.bam -o bamPath/sampleId.sort.bam
6. samtools index bamPath/sampleId.sort.bam

**Callers:**

# cuteSV call SV:

**ONT:** cuteSV --genotype --max_cluster_bias_INS 100 --diff_ratio_merging_INS 0.3 --max_cluster_bias_DEL 100 --diff_ratio_merging_DEL 0.3 -t ThreadNumber -s SReads -l SvLength bamPath/sampleId.svTools.sort.bam GenomeIndexPath/Genome.fa outPath/sampleId.vcf outPath

**CCS:** cuteSV --genotype --max_cluster_bias_INS 1000 --diff_ratio_merging_INS 0.9 --max_cluster_bias_DEL 1000 --diff_ratio_merging_DEL 0.5 -t ThreadNumber -s SReads -l SvLength bamPath/sampleId.svTools.sort.bam GenomeIndexPath/Genome.fa outPath/sampleId.vcf outPath

**CLR:** cuteSV --genotype --max_cluster_bias_INS 100 --diff_ratio_merging_INS 0.3 --max_cluster_bias_DEL 200 --diff_ratio_merging_DEL 0.5 -t ThreadNumber -s SReads -l SvLength bamPath/sampleId.svTools.sort.bam GenomeIndexPath/Genome.fa outPath/sampleId.vcf outPath

# cuteSV2 call SV:

**ONT:** cuteSV --max_cluster_bias_INS 100 --diff_ratio_merging_INS 0.3 --max_cluster_bias_DEL 100 --diff_ratio_merging_DEL 0.3 -t ThreadNumber -s SReads -l SvLength bamPath/sampleId.svTools.sort.bam GenomeIndexPath/Genome.fa outPath/sampleId.vcf outPath

**CCS:** cuteSV --genotype --max_cluster_bias_INS 1000 --diff_ratio_merging_INS 0.9 --max_cluster_bias_DEL 1000 --diff_ratio_merging_DEL 0.5 -t ThreadNumber -s SReads -l SvLength bamPath/sampleId.svTools.sort.bam GenomeIndexPath/Genome.fa outPath/sampleId.vcf outPath

**CLR:** cuteSV --genotype --max_cluster_bias_INS 100 --diff_ratio_merging_INS 0.3 --max_cluster_bias_DEL 200 --diff_ratio_merging_DEL 0.5 -t ThreadNumber -s SReads -l SvLength bamPath/sampleId.svTools.sort.bam GenomeIndexPath/Genome.fa outPath/sampleId.vcf outPath

# DeBreak call SV:

**ONT, CCS, CLR:** debreak --bam bamPath/sampleId.sort.bam --depth SqDepth --min_support SReads --thread ThreadNumber --min_size SvLength -r GenomeIndexPath/Genome.fa -o outPath/sampleId -p sampleId

# DELLY call SV:

**ONT:** delly lr -t ALL -y ont -o outPath/sampleId.bcf -g GenomeIndexPath/Genome.fa bamPath/sampleId.svTools.sort.bam

**CCS, CLR:** delly lr -t ALL -y pb -o outPath/sampleId.bcf -g GenomeIndexPath/Genome.fa bamPath/sampleId.svTools.sort.bam

bcftools view outPath/sampleId.bcf >outPath/sampleId.vcf

# NanoSV call SV:

**ONT, CCS, CLR:** NanoSV -t ThreadNumber -b /Ref/hs37d5.bed -o outPath/sampleId.vcf bamPath/sampleId.svTools.sort.bam

# NanoVar call SV:

**ONT:** nanovar -x ont -c SReads -t ThreadNumber -l SvLength bamPath/sampleId.svTools.sort.bam GenomeIndexPath/Genome.fa outPath/sampleId/sampleId

**CCS:** nanovar -x pacbio-ccs -c SReads -t ThreadNumber -l SvLength bamPath/sampleId.svTools.sort.bam GenomeIndexPath/Genome.fa outPath/sampleId/sampleId

**CLR:** nanovar -x pacbio-clr -c SReads -t ThreadNumber -l SvLength bamPath/sampleId.svTools.sort.bam GenomeIndexPath/Genome.fa outPath/sampleId/sampleId

# pbsv call SV:

1. **ONT, CCS, CLR:** pbsv discover bamPath/sampleId.svTools.sort.bam outPath/sampleId.svsig.gz --tandem-repeats /Ref/human_hs37d5.trf.bed
2. **ONT, CCS, CLR:** pbsv call --gt-min-reads SReads -m SvLength -j ThreadNumber GenomeIndexPath/Genome.fa outPath/sampleId.svsig.gz outPath/sampleId.vcf

# Picky call SV:

1. **ONT, CCS, CLR:** samtools sort -n bamPath/sampleId.svTools.sort.bam|samtools view -Sh |perl picky.pl sam2align >outPath/sampleId.align
2. **ONT, CCS, CLR:** cat outPath/sampleId.align|perl picky.pl callSV --fastq sample.fq --genome GenomeIndexPath/Genome.fa --oprefix outPath/sampleId
3. ls outPath/*.xls|grep -v profile.xls|while read id;do(perl picky.pl xls2vcf --re 2 --xls $id >$id.vcf);done
4. cat <(grep \# outPath/*.profile.INS.xls.vcf) <(cat outPath/*xls.vcf|grep -v \#)|vcf-sort >outPath/sampleId.vcf

# Sniffles call SV:

**ONT, CCS, CLR:** sniffles -s SReads -l SvLength -t ThreadNumber -m bamPath/sampleId.svTools.sort.bam -v outPath/sampleId.vcf

# Sniffles2 call SV:

**ONT, CCS, CLR:** sniffles2 --minsvlen SvLength -t ThreadNumber -i bamPath/sampleId.svTools.sort.bam -v outPath/sampleId.vcf

# svim call SV:

**ONT, CCS, CLR:** svim alignment --minimum_depth SReads --min_sv_size SvLength outPath/sampleId/sampleId bamPath/sampleId.svTools.sort.bam GenomeIndexPath/Genome.fa

# svision call SV:

**ONT, CCS, CLR:**SVision -o outPath/sampleId/ -b outPath/sampleId/sampleId bamPath/sampleId.svTools.sort.bam -m /model/svision-cnn-model.ckpt -g GenomeIndexPath/Genome.fa -n sampleId -s SReads --graph --qname -t ThreadNumber

# Parameters description:

| **sample.fq** | **:** | **Sample fastq file** |
| --- | --- | --- |
| **sampleId** | **:** | **Sample ID** |
| **outPath** | **:** | **Output file path** |
| **GenomeIndexPath** | **:** | **Genome index Path** |
| **SqDepth** | **:** | **Sequencing depth** |
| **sampleId.svTools.sort.bam** | **:** | **Bam file** |
| **SReads** | **:** | **Support reads number** |
| **ThreadNumber** | **:** | **Number of threads** |
| **SvLength** | **:** | **Min SV size** |
