# Supplementary material for: Comprehensive and deep evaluation of structural variation detection pipelines with third-generation sequencing data
Source: Genome Biol. 2024 Jul 15;25:188. doi: 10.1186/s13059-024-03324-5 (PMC11247875; doi:10.1186/s13059-024-03324-5)
Supplement: Supplementary file 6 — Additional file 6. Review history. [file 13059_2024_3324_MOESM6_ESM.docx]

**1^st^ round**

**Reviewer 1**

In this paper, Zhi Liu et al aim to evaluate Structural Variation detection pipelines with long read sequencing data. Structural Variation (SV) is an important component of genetic variation and has been increasingly studied in recent years. Detection of SVs remains challenging even with long reads and the numerous tools that have been developed to detect them can have highly variable performance and detection quality depending on SV types and sequencing data features. Therefore, such efforts to compare and evaluate SV detection methods on comprehensive SV sets and conditions are highly valuable and very useful to the community. There are few such benchmarks in the litterature and this is of broad interest to many in the field.

The strengths of this work are the exhaustive set of methods (n=39), obtained by combining 4 read mappers with 10 SV callers, and the numerous important factors that are investigated, such as SV types, breakpoint precision, sequencing depth, genotypes...

However, I found many flaws in the paper by Zhi Liu et al, including the choice and generation of data for evaluation, the poorly readable presentation of the results and the lack of precision and clarity of the paper. In my opinion, this paper is not of sufficient quality to be published in Genome Biology.

Below is a selection of my main major concerns:

1. Simulated SV sets. A critical feature that affects SV calling performance is the type, sequence content, size and genomic location of the SVs. Whether all these features are randomly selected or follow real SV distributions can dramatically change the performance of SV calling. However, the authors do not describe how the simulated SV features were sampled in their simulated datasets. Are the genomic locations sampled uniformly along the genome or do they follow true SV call set position distributions? What is the size distribution of the simulated SVs? What type of sequence is chosen for insertion events ? This is important information that is critically missing from the manuscript.

2. SV truth sets. Except for one individual (HG002), if I understood correctly, all other SV truth sets of the real datasets are obtained by running the same later evaluated methods on the same sequencing datasets and combining their results. This is problematic because this truth set is likely to miss many real SVs, and especially the most difficult ones to call, and may also contain false positive calls that are recurrently and similarly produced by several methods. This prevents a comprehensive and fair evaluation of the methods. Furthermore, no statistics are given on these truth sets that are not publicly available, e.g. how many of each SV type ? Also, how the SV truth sets are obtained should be clearly stated at the beginning of the Result section and better explained in the Method section.

3. SV truth sets. Important community efforts have produced several real human datasets with high quality SV call sets dedicated to SV benchmarking (obtained using high depth sequencing data from different sequencing platforms, and that were carefully curated), such as Genome In A Bottle and HGSVC. I wonder why the authors did not use such datasets for all their real datasets and I strongly recommend them to perform their benchmark with such SV truth sets.

4. The state of the art on SV method benchmarking is poorly developed in the introduction and some important references are missing. For instance, the benchmark paper by Dierckxsens et al, Genome Biology 2021 (doi:10.1186/s13059-021-02551-4) is not cited, even though it is the work most closely related to this study. The authors need to show how their work compare to this one, in which aspects it is similar or different, and what is the added value of their work with respect to this previous important contribution. There is also a critical lack of mention of real publicly available resources dedicated to SV benchmarking, such as the Genome In A Bottle datasets and the Human Genome Structural Variation Consortium.

5. Results of combining several methods. I could not find out on which datasets the shown results of combining multiple pipelines were performed (this information is missing in the main text). If this was done on the real datasets, where the truth sets are obtained by exactly the same method of combining multiple pipelines, it is not surprising that combinations perform better. And this benchmark result does not demonstrate the performance gain of merging approaches. Furthermore, the authors claim that this is a novel finding (l. 2 and 30 p. 17 "we discovered"), although numerous studies have already pointed this out for short and long read SV callers (e.g. Zhou 2019 and Dierckxsens 2021).

6. Result presentation. Evaluation results are presented mainly in large figures, which are difficult to read and interpret due to the large number of pipelines (X axis) and of the mixing of different metrics or features. The trends described in the result sections could be more clearly illustrated in figures by selecting specific pipelines and/or features (e.g. Figures 1-3). The supplementary file contains too many supplementary figures, 38, each with multiple panels. I strongly recommend the authors to make more succinct figures, by selecting the important features to show, in order to guide the reader towards the important messages and findings of the benchmark. Additionally, many figures cannot be interpreted without reading the legend several times, the differences between the panels should be visible on the figure itself. In Figure 4, the x-axis and colour legend are not explained.

7. Evaluation metrics. The paragraph explaining how recall, precision and F1 measures are calculated is confusing, I did not understand if they were only obtained from truvari, as another procedure is described that relies on chunks and thresholds that are not defined (p.15 l.26-29). Most figures show only the F1 value, which is useful for ranking the tools, but does not distinguish between tools that are more specific or more sensitive. This is an important distinction as users may prefer one or the other depending on the application.

8. SV types. One important type of SV is not included in the study: translocations or BNDs. The justification given by the authors is not convincing (translocations are recorded as breakpoints by SV callers). The evaluation could be done at the level of the breakpoints and not at the level of the translocation events. Also, I do not understand what a DUP is in this study. A duplication is the insertion of a duplicated copy. How do the authors differentiate between INS and DUP? Many SV callers report duplication events as insertions, and some output only specific types of duplications as DUP, such as tandem duplications. However, since not all SV callers classify these different types of duplications and insertions in the same way, how was this issue addressed in this benchmark?

9. Absence of HiFi data. PacBio HiFi technology is now increasingly being used to detect and study SVs. Authors should include such sequencing data, as many human datasets are publicly available for the individuals included in their study.

10. Writing. The writing of the manuscript needs to be improved as I found it difficult to read as such. Some sentences are difficult to read because of long lists of tool names or numbers. I found many sentences that are grammatically incorrect, for example without a verb (for example: p.4 l.16, p.4 l.35-39, p.12 l.1-4, p.14 l.4, p.17 l.31).

**Reviewer 2**

The manuscript presents a relatively thorough study to date on evaluating pipelines (combinations of aligners and callers) for SV detection, considering 11 factors, including breakpoint deviation and genotype accuracy, and comprehensively compares the performance of different combinations of aligners and callers for SV detection on third-generation sequencing. The manuscript reveals efficient ways to combine pipelines for improving performance and identifies critical properties of called SVs. However, there are still some doubts for me that need to be explained by the author:

1. In the manuscript, the human reference genome of hs37d5 version was used to simulation and benchmarking. As we all know, the GRCh38 version of the reference genome is more comprehensive and accurate than the GRCh37 version and is the most widely used in current genome studies. Moreover, the recently proposed T2T reference genome has also attracted considerable attention. Thus, why the author chooses the hs37d5 as the reference genome instead of GRCh38 and T2T.

2. It is not easy to conclude that "The different results between simulated and real datasets suggested that the historical superiority of PacBio over Nanopore in SV detection may no longer exist." from the manuscript. First, for both real and simulated datasets, the author still declare that the performance of PacBio datasets were better than that of Nanopore data. Second, the current widely used PacBio data include HiFi (High accuracy) and CLR (Low accuracy), what the pacbio2016 model of VISOR represented? Similar, the accuracy of ONT data deeply influenced by the nanopores (R9 or R10) and the chemistry, so the nanopore2020 model of VISOR can represent the latest of ONT data generation model. Thus, in this manuscript, it insufficient to benchmark the comparison of PacBio and ONT platform. If you really want, more supplement experiments and result data are need.

3. As far as I know, "LRA+cuteSV" and "Minimap2+sniffles2 (v2)" were the officially recommended pipelines of ONT at different stages. But the author excludes the LRA aligner and sniffles2 caller in the manuscript. Moreover, sniffles2 is the upgraded version of sniffles, which has significant improved in the SV detection and genotype assignment. Thus, the author should consider to add LRA and sniffles2 to the benchmark and expect to obtain consistent and advanced results with ONT recommend "minimap2+sniffles2" pipeline.

4. A comprehensive evaluation of SV calling pipeline should also consider the running time and memory usage. The author seems to have forgotten this.

Additionally, there are some typo errors in the manuscript. For example, in Abstract, the "minimap2-cutsv" should be "minimap2-cuteSV", and so on.

**Authors’ response**

Reviewer 1

In this paper, Zhi Liu et al aim to evaluate Structural Variation detection pipelines with long read sequencing data. Structural Variation (SV) is an important component of genetic variation and has been increasingly studied in recent years. Detection of SVs remains challenging even with long reads and the numerous tools that have been developed to detect them can have highly variable performance and detection quality depending on SV types and sequencing data features. Therefore, such efforts to compare and evaluate SV detection methods on comprehensive SV sets and conditions are highly valuable and very useful to the community. There are few such benchmarks in the litterature and this is of broad interest to many in the field.

The strengths of this work are the exhaustive set of methods (n=39), obtained by combining 4 read mappers with 10 SV callers, and the numerous important factors that are investigated, such as SV types, breakpoint precision, sequencing depth, genotypes…

However, I found many flaws in the paper by Zhi Liu et al, including the choice and generation of data for evaluation, the poorly readable presentation of the results and the lack of precision and clarity of the paper. In my opinion, this paper is not of sufficient quality to be published in Genome Biology.

1. Simulated SV sets. A critical feature that affects SV calling performance is the type, sequence content, size and genomic location of the SVs. Whether all these features are randomly selected or follow real SV distributions can dramatically change the performance of SV calling. However, the authors do not describe how the simulated SV features were sampled in their simulated datasets. Are the genomic locations sampled uniformly along the Genome or do they follow true SV call set position distributions? What is the size distribution of the simulated SVs? What type of sequence is chosen for insertion events ? This is important information that is critically missing from the manuscript.

**Response**: we are grateful for the suggestion. According to your advice, we have added a more detailed interpretation regarding the simulation SV type, sequence content, size and genomic location. We have provided detailed explanations for all aspects of the study design review in the "Study Design Review" section of our manuscript. Below are the specific contents:

The evaluation was carried out based on both real and simulated datasets. The simulated dataset comprised 20541 non-overlapping structural variations, including 7214 deletions, 9989 insertions and 51 inversions identified within the CHM1 sample call set (dbVAR database accession nstd137， including INS sequence). Additionally, 2919 duplications and 368 translocations were extracted from the KWS1 sample call set (dbVAR database accession nstd106) [1]. The length distributions of DEL and INS are similar, with two main peaks at 50bp and 300bp. DUP lengths are mainly concentrated in the ranges of 50bp and 3000bp. The peaks of INV are located at 360bp and 10kb(Figure S1-2). All simulated TRA lengths are 10kb. The simulated SV distribution on the genome is generally consistent with the distribution of SVs in real data(**Figure S2**). The VISOR(v1.1) [2] tool was utilized to generate creating simulation reads( Pacbio: CCS, CLR; Nanopore: R9.4, R10.4), using the human reference genome (version: GRCh38). Two platforms, PacBio and Nanopore, for the simulation mean read lengths of 8kb, default error models). As from real datasets, the PacBio CCS datasets included samples from CHM13, HG00096, HG002, HG003, HG004, HG00512, HG005, HG006, HG007 and NA12878 with average read lengths ranging from 10kb to 18kb (**Table S2**), while the CLR datasets included CHM13, HG002, HG003, HG004, HG00512, HG005, HG006 and HG007 with average read lengths ranging from 8kb to 25kb. The Nanopore datasets included samples from CHM13, HG00096, HG002, HG003, HG004, HG00512 and NA12878, with average read lengths ranging from 8kb to 56kb (**Table S2**). Additionally, real family data samples were also used for the assessment: Nanopore (HG002(son), HG003(father), HG004(mother)), PacBio (HG002(son), HG003, HG004), and (HG005(son), HG006(father), HG007(mother)).

The high-confidence benchmark dataset of real data is sourced from publicly available datasets and intersecting multiple pipelines (IMP). For samples HG002 (GIAB), CHM13(HPGP), HG00096, HG00512, and NA12878( HGSV), the benchmarks for DEL and INS are obtained from public datasets. Additionally, for sample HG002, benchmarks for DUP, INV, and TRA are sourced from the work of Melanie Kirsche et al. [3]. Because we found that benchmarks for other sample-specific structural variant types were unavailable, we created benchmarks using IMP for the corresponding SV types of the sample (see the "Methods" section for details). The IMP benchmark has more SV than the real benchmark for deletions (over 1900) and insertions (over 900). The counts of DUP and BND are similar between the IMP and real benchmarks. The number of inversions (INV) in the IMP benchmark is closer to the simulation count (50~70), whereas the real benchmark for HG002 is 689 (**Table S3**).

**Supplementary Fig1 (Sim, CHM13)**. The SV length distribution in benchmark datasets includes both simulated (Sim) and real samples.

**Supplementary Fig2(Sim, CHM13)**. Location distribution of SV on the GRCh38 genome on the benchmark datasets of simulated (Sim) and real samples.

2. SV truth sets. Except for one individual (HG002), if I understood correctly, all other SV truth sets of the real datasets are obtained by running the same later evaluated methods on the same sequencing datasets and combining their results. This is problematic because this truth set is likely to miss many real SVs, and especially the most difficult ones to call, and may also contain false positive calls that are recurrently and similarly produced by several methods. This prevents a comprehensive and fair evaluation of the methods. Furthermore, no statistics are given on these truth sets that are not publicly available, e.g. how many of each SV type ? Also, how the SV truth sets are obtained should be clearly stated at the beginning of the Result section and better explained in the Method section.

**Response:** Thank you for raising this important question. Indeed, there are some issues with constructing benchmarks by intersecting multiple pipelines(IMP). To ensure a comprehensive and fair evaluation, we have taken two approaches. Firstly, we have chosen more reliable public datasets such as HG002, CHM13, HG00096, HG00512, and NA12878 as benchmarks. Secondly, when constructing benchmarks by intersecting multiple pipelines, we have adjusted the number of supported callers based on the quantities of different types of structural variants (SVs) in real benchmarks. This helps minimize issues arising from intersecting pipelines. Since most pipelines exhibit high accuracy in detecting deletions (DEL) and insertions (INS), using too many supported callers might overlook many true SVs. Thus, for DEL and INS benchmarks, we selected an appropriate number of supported callers to exceed the number of real benchmarks slightly. Conversely, as most pipelines perform less performance for duplications (DUP), inversions (INV), and breakpoints (BND), we need to use a higher number of supported callers to ensure dataset accuracy. Regarding your concern about potentially consistent false positive calls in the IMP benchmark from various pipelines, the IMP method cannot address this issue alone. We rely on supplementing with new real sample benchmarks(CHM13, HG00096, HG00512, and NA12878). Finally, we have taken your suggestion to heart and publicly disclosed all statistical data(SVs number, length distribution, genome location) for our benchmarks. In the "Study design review" section of our paper, we clearly explain how different samples and SV types were obtained and provide more detailed explanations in the methods section.

**Table S3:**

| **Sample** | **DEL** | **INS** | **INV** | **DUP** | **DUP_INS** | **BND** |
| --- | --- | --- | --- | --- | --- | --- |
| CHM13 | 7622 | 12448 | 55 | 275 | 12723 | 166 |
| HG00096 | 6151 | 10091 | 71 | 336 | 10427 | 132 |
| HG002 | 5418 | 7266 | 689 | 374 | 7640 | 186 |
| HG003 | 8366 | 10750 | 70 | 282 | 11032 | 199 |
| HG004 | 8523 | 10765 | 79 | 277 | 11042 | 150 |
| HG005 | 8534 | 10733 | 62 | 364 | 11097 | 195 |
| HG00512 | 6135 | 10118 | 65 | 314 | 10432 | 313 |
| HG006 | 8539 | 10883 | 67 | 278 | 11161 | 130 |
| HG007 | 8432 | 10986 | 67 | 289 | 11275 | 109 |
| NA12878 | 6115 | 9956 | 65 | 385 | 10341 | 150 |

3. SV truth sets. Important community efforts have produced several real human datasets with high-quality SV call sets dedicated to SV benchmarking (obtained using high-depth sequencing data from different sequencing platforms, and that were carefully curated), such as Genome In A Bottle and HGSVC. I wonder why the authors did not use such datasets for all their real datasets and I strongly recommend them to perform their benchmark with such SV truth sets.

**Response:** Thank you very much for your suggestion. We use samples from Genome In A Bottle (HG002) and HGSVC (HG00512, HG00096, NA12878) as benchmarks.

Initially, we primarily used samples and benchmarks from the Genome In A Bottle project. However, since only the DEL and INS benchmarks for the HG002 sample were widely available in Genome In A Bottle, we couldn't find usable SV benchmarks for HG003, HG004, HG005, HG006, and HG007. As a result, we ultimately constructed benchmarks for HG003 to HG007 samples, as well as CHM13 and NA12878, by intersecting multiple pipelines(IMP). After seeing your suggestion, we found benchmarks for the NA12878 sample in the HGSVC project and added samples HG00512 and HG00096 for evaluation. However, we only found DEL and INS benchmarks in HGSVC. For the benchmarks of DUP, INV, and BND that we cannot find, we can only use the IMP method. For the well-studied HG002 sample, we found an SV dataset based on de novo assembly in the research of Melanie Kirsche et al. [3]. Using this dataset, we constructed DUP, INV, and BND benchmarks. Additionally, we found usable DEL and INS benchmarks for the CHM13 sample in the HPGP project.

4. The state of the art on SV method benchmarking is poorly developed in the introduction and some important references are missing. For instance, the benchmark paper by Dierckxsens et al, Genome Biology 2021 (doi:10.1186/s13059-021-02551-4) is not cited, even though it is the work most closely related to this study. The authors need to show how their work compare to this one, in which aspects it is similar or different, and what is the added value of their work with respect to this previous important contribution. There is also a critical lack of mention of real publicly available resources dedicated to SV benchmarking, such as the Genome In A Bottle datasets and the Human Genome Structural Variation Consortium.

**Response**: Thank you for your suggestion. We have incorporated the research advancements and citations from the benchmark paper by Dierckxsens et al, Genome Biology 2021 (doi:10.1186/s13059-021-02551-4) into our study. This modification is located in the third paragraph of the "Introduction" section, with the specific details as follows:

"Dierckxsens et al. developed Sim-it, a tool for simulating SVs and long reads, and evaluated the strengths and weaknesses of 7 callers and long-read sequencing platforms[4]. Their study introduced a new method called combiSV, which combines results from SV callers into a higher-quality call set with improved recall and precision. However, these evaluations mainly focused on callers and a few aligners, lacking analysis of the impact of aligners on SV detection. In addition to existing evaluation studies, available benchmark resources for SVs are gradually increasing, such as Genome in a Bottle (GIAB), Human Genome Structural Variation Consortium (HGSV), and The Human Pangenome Reference Consortium (HPRC)."

5. Results of combining several methods. I could not find out on which datasets the shown results of combining multiple pipelines were performed (this information is missing in the main text). If this was done on the real datasets, where the truth sets are obtained by exactly the same method of combining multiple pipelines, it is not surprising that combinations perform better. And this benchmark result does not demonstrate the performance gain of merging approaches. Furthermore, the authors claim that this is a novel finding (l. 2 and 30 p. 17 "we discovered"), although numerous studies have already pointed this out for short and long read SV callers (e.g. Zhou 2019 and Dierckxsens 2021).

**Response**: In our revisions, we utilized publicly available datasets (CHM13, HG002, HG00512, HG00096, NA12878) as benchmarks. We made a mistake in our wording, but it has now been removed(raw 1.2 p.17) and corrected(raw 30 p.17).

The initial benchmarks were obtained by intersecting multiple pipelines. In the revised results, we evaluated the fusion of multiple pipelines using benchmark datasets from HG002, CHM13, HG00096, HG00512, and NA12878 samples. The benchmarks for DEL and INS come from real data, while those for INV and DUP are obtained through the intersection of multiple pipelines (except for HG002).

6. Result presentation. Evaluation results are presented mainly in large figures, which are difficult to read and interpret due to the large number of pipelines (X axis) and of the mixing of different metrics or features. The trends described in the result sections could be more clearly illustrated in figures by selecting specific pipelines and/or features (e.g. Figures 1-3). The supplementary file contains too many supplementary figures, 38, each with multiple panels. I strongly recommend the authors to make more succinct figures, by selecting the important features to show, in order to guide the reader towards the important messages and findings of the benchmark. Additionally, many figures cannot be interpreted without reading the legend several times, the differences between the panels should be visible on the figure itself. In Figure 4, the x-axis and colour legend are not explained.

**Response**: Thank you for your suggestion. We acknowledge that presenting all the figures together may pose challenges for readability. However, considering the large number of pipelines we evaluated, splitting the figures based on features or pipelines would significantly increase the number of supplementary figures, causing further inconvenience for readers. In addition, presenting the results of all pipelines in a single figure may facilitate a more straightforward comparison.

Nevertheless, we have simplified some of the figures by merging results from pipelines with similar trends when possible. For example, the **Supplementary Fig9** **a-c**. in the previous manuscript has been modified to the new version.

**Supplementary Fig9** **a-c**. Effect of sequencing depth on recall, precision and F1 of different SV detection pipelines. Sequencing depth gradients of 5x,10x,15,25x. a: recall; b: precision; c: F1.

The Figure 3 in the previous manuscript has been moved to the supplementary figures(**Supplementary Fig11**) in the revised manuscript because the pipelines seemed less sensitive to the SV size. We have added explanations for the colour legend of the x-axis in Figure 4(now it is Figure 3 in the revised manuscript).

**Figure3**. Distribution of SV length range and length deviation of DEL in different pipelines. The legend depicts colours ranging from deep blue to red, representing different deviation scales (>50bp, 50:10bp, 10:0bp, 0bp, 0:-10bp, -10:-50bp, <-50bp). The x-axis represents five length intervals of SV size (50-100bp, 100-500bp, 500-1kb, 1kb-2.5kb, >2.5kb). The y-axis represents the proportion of different deviation scales within the corresponding length ranges.

7. Evaluation metrics. The paragraph explaining how recall, precision and F1 measures are calculated is confusing, I did not understand if they were only obtained from truvari, as another procedure is described that relies on chunks and thresholds that are not defined (p.15 l.26-29). Most figures show only the F1 value, which is useful for ranking the tools, but does not distinguish between tools that are more specific or more sensitive. This is an important distinction as users may prefer one or the other depending on the application.

**Response**: In the revision, the recall, precision, and F1 scores for DEL, INS, INV, and DUP evaluation come from Truvari. In the revision, we added recall-measure and precision-measure figures for the pipelines.

For BND, if the SV detected by the pipeline meets the conditions of Eq.1, it is considered a true positive(TP) call at the breakpoint level; otherwise, it is a false positive(FP). A ground benchmark SV is labelled as a false negative (FN) if no SV call satisfies Eq.1, following the method proposed by Tao Jiang et al. [1]. Next, we calculated pipelines' accuracy, recall, and F1 score for BND based on Eq2-4.

{compBK1-baseBK1≤1kb compBK2-baseBK2≤1kb compchr1=basechr1 compchr2=basechr2	(1)

Where "comp" refers to the pipeline, while "base" refers to the benchmark. "BK1," "BK2," "Chr1," and "Chr2" represent breakpoints and chromosomes.

Prediction is defined as

Precision=TPTP+FP	(2)

Recall is defined as

Recall=TPTP+FN	(3)

F1 score is defined as

F1=2*Precision*RecallPrecision+Recall	(4)

**Supplementary Fig6**. Performance of SV detection pipelines in different SV types. Precision and recall of DEL, DUP, INS, INV and BND were determined with the simulated (**a**(precision), **c**(recall)) and the real data(**b**(precision), **d**(recall)). Modified precision-measure and recall-measure are shown for the pipelines indicated with orange (for DEL), yellow (for INS), green (for DUP), brown (for INV) and olive(for BND)bars.

8. SV types. One important type of SV is not included in the study: translocations or BNDs. The justification given by the authors is not convincing (translocations are recorded as breakpoints by SV callers). The evaluation could be done at the level of the breakpoints and not at the level of the translocation events. Also, I do not understand what a DUP is in this study. A duplication is the insertion of a duplicated copy. How do the authors differentiate between INS and DUP? Many SV callers report duplication events as insertions, and some output only specific types of duplications as DUP, such as tandem duplications. However, since not all SV callers classify these different types of duplications and insertions in the same way, how was this issue addressed in this benchmark?

**Response**: In the revision, we added breakpoints (BND) to the evaluation of SV types. In this study, we defined duplications (DUP) as SV records in the caller output VCF file with "DUP" in the SVTYPE field（Such as DUP, tDUP, DUP+INS, etc.）. However, there are issues: some callers report duplication events as insertions, some output only specific types of duplications as DUP, and not all SV callers classify these events consistently. To facilitate comparison and analysis of duplications across different pipelines, we unified SV records from different callers with "DUP" in the SVTYPE field as DUP. This addresses the diversity in DUP classification among callers but doesn't resolve the issue of some callers reporting DUP as INS. Therefore, we evaluated all DUP as INS and detected INS together (treating DUP as INS for evaluation).

**Figure1 (a, b)**. Performance of SV detection pipelines in different SV type. Precision and recall of DEL, DUP, INS, INV and BND were determined with the simulated (**a**) and the real data(b, **d**). F1-measures (the combined statistics for precision and recall (see the "Methods" section for details)) are shown for the pipelines indicated with orange (for DEL), yellow (for INS), green (for DUP), brown (for INV) and olive(for BND)bars.

**Supplementary Fig5**. Pipelines performance in DUP_INS. F1-measure for DUP and DUP_INS in simulated and real datasets. **a** Real data; **b** Simulated data

9. Absence of HiFi data. PacBio HiFi technology is now increasingly being used to detect and study SVs. Authors should include such sequencing data, as many human datasets are publicly available for the individuals included in their study.

**Response**: In the revision, we utilized Pacbio HiFi data. We've included PacBio platform data of two types: CLR (Low accuracy) (CHM13, HG002, HG003, HG004, HG00512, HG005, HG006, and HG007) and CCS (HiFi) (CHM13, HG00096, HG002, HG003, HG004, HG00512, HG005, HG006, HG007, and NA12878).

10. Writing. The writing of the manuscript needs to be improved as I found it difficult to read as such. Some sentences are difficult to read because of long lists of tool names or numbers. I found many sentences that are grammatically incorrect, for example without a verb (for example: p.4 l.16, p.4 l.35-39, p.12 l.1-4, p.14 l.4, p.17 l.31).

**Response**: Thank you very much for reviewing our manuscript and providing valuable suggestions and feedback. We greatly appreciate your thorough work. We acknowledge that the manuscript has grammar and writing errors, and we have taken measures to correct them. The following are corrections to the mentioned examples.

p.4 l.16：

We have deleted the error on p.4 l.16, after rewriting the section.

p.4 l.35-39：

In the simulation dataset, some pipelines achieve an F1 score exceeding 0.95(DEL, INS), such as lra-cuteSV, minimap2-DeBreak, and pbmm2-DeBreak. In real datasets, five pipelines achieve an F1 score greater than 0.89(DEL, INS, **Figure 1**), such as minimap2-cuteSV, minimap2-cuteSV2, minimap2-Sniffles2, pbmm2-cuteSV, pbmm2-cuteSV2, and winnowmap-cuteSV.

p.12 l.1-4:

In the top 10 combinations of two pipelines, three combinations demonstrate a good F-measure. (ngmlr-cuteSV.ngmlr-NanoVar(3.16), pbmm2-cuteSV.pbmm2-cuteSV2(3.16), and winnowmap-cuteSV.winnowmap-cuteSV2(3.12)). Meanwhile, in the top 10 combinations of three pipelines, three combinations demonstrate excellent performance(ngmlr-Sniffles2.ngmlr-NanoVar.ngmlr-SVision(3.21), ngmlr-cuteSV2.ngmlr-Sniffles2.ngmlr-NanoVar(3.21), and ngmlr-cuteSV2.ngmlr-Sniffles2.ngmlr-SVision(3.2)).

p.14 l.4:

We have deleted the error on p.4 l.16, after rewriting the section.

p.17.l.31:

This is especially true when combining pipelines utilizing the same aligner, as it improves efficiency and diminishes storage demands.

Reviewer 2

The manuscript presents a relatively thorough study to date on evaluating pipelines (combinations of aligners and callers) for SV detection, considering 11 factors, including breakpoint deviation and genotype accuracy, and comprehensively compares the performance of different combinations of aligners and callers for SV detection on third-generation sequencing. The manuscript reveals efficient ways to combine pipelines for improving performance and identifies critical properties of called SVs. However, there are still some doubts for me that need to be explained by the author:

1. In the manuscript, the human reference genome of hs37d5 version was used to simulation and benchmarking. As we all know, the GRCh38 version of the reference genome is more comprehensive and accurate than the GRCh37 version and is the most widely used in current genome studies. Moreover, the recently proposed T2T reference genome has also attracted considerable attention. Thus, why the author chooses the hs37d5 as the reference genome instead of GRCh38 and T2T.

**Response**: Thank you very much for raising this question. We updated the version of the Genome for evaluation from hs37d5 to GRCh38.

Initially, we mainly considered using samples from the Genome In A Bottle (GIAB) database, which primarily utilizes the hs37d5 reference genome. Therefore, we stuck with hs37d5. However, hs37d5 falls short in terms of genome completeness, accuracy, and widespread application compared to GRCh38. Additionally, it is not as complete or accurate as the T2T genome. The use of T2T genomes is expected to be a major trend in the future. Considering your question, we took into account the availability of public benchmarks corresponding to reference genomes, as well as the completeness, accuracy, and widespread application of these genomes. As a result, we decided to use the GRCh38 genome in our modifications. While T2T genomes have higher completeness and accuracy compared to GRCh38 and are expected to have broader applications, the number of available public sample benchmarks for T2T is significantly lower than for GRCh38. Although SV benchmarks can be converted between different versions of reference genomes using LiftOver, this conversion is not lossless. Therefore, we used GRCh38 in the revised manuscript.

2. It is not easy to conclude that "The different results between simulated and real datasets suggested that the historical superiority of PacBio over Nanopore in SV detection may no longer exist." from the manuscript. First, for both real and simulated datasets, the author still declare that the performance of PacBio datasets were better than that of Nanopore data. Second, the current widely used PacBio data include HiFi (High accuracy) and CLR (Low accuracy), what the pacbio2016 model of VISOR represented? Similar, the accuracy of ONT data deeply influenced by the nanopores (R9 or R10) and the chemistry, so the nanopore2020 model of VISOR can represent the latest of ONT data generation model. Thus, in this manuscript, it insufficient to benchmark the comparison of PacBio and ONT platform. If you really want, more supplement experiments and result data are need.

**Response**: Thank you for pointing out this issue. We have removed this conclusion. After careful consideration and using various data types (R10, R9, CCS, CLR), we still cannot draw this conclusion. In the revision, we used two types of PacBio data: CLR (with lower accuracy) and CCS (HiFi). The PacBio2016 model in Visor represents simulated data based on CLR type. For ONT, in simulated data, we used Visor's Nanore2020 (R9.4) and Nanore2023 (R10.4) models. In real ONT data, due to a lack of the latest R10 samples, we mainly relied on R9 samples. To understand how R10 and R9 data perform in calling SVs in real samples, we used the HG002 sample chr20 chromosome data (R9.4, R10.4) from the study by Kishwar Shafin et al[5].

**Supplementary Fig8 f, g, h**. F1 Distribution of pipelines in Pacbio and Nanopore sample data under 25x sequencing depth. **f**:ALL(DEL, INS, INV, DUP), **g**:HG002 chr20 DEL, **h**:HG002 chr20 INS

3. As far as I know, "LRA+cuteSV" and "Minimap2+sniffles2 (v2)" were the officially recommended pipelines of ONT at different stages. But the author excludes the LRA aligner and sniffles2 caller in the manuscript. Moreover, sniffles2 is the upgraded version of sniffles, which has significant improved in the SV detection and genotype assignment. Thus, the author should consider to add LRA and sniffles2 to the benchmark and expect to obtain consistent and advanced results with ONT recommend "minimap2+sniffles2" pipeline.

**Response**: In the revision, we've taken your suggestion to include the LRA aligner and Sniffles2 caller in the pipeline evaluation. Additionally, in genotype-based evaluation, we found that Sniffles2 indeed improves genotype accuracy compared to Sniffles. In our evaluation results, we indeed found that minimap2-Sniffles2 exhibits higher precision compared to lra-cuteSV.

**Supplementary Fig6**. Performance of SV detection pipelines in different SV types. Precision and recall of DEL, DUP, INS, INV and BND were determined with the simulated (**a**: precision) and the real data(**b**: precision). Precision-measure are shown for the pipelines indicated with orange (for DEL), yellow (for INS), green (for DUP), brown (for INV) and olive(for BND)bars. Pipelines are categorized according to the alignment tools (lra, minimap2, ngmlr, pbmm2, winnowmap).

4. A comprehensive evaluation of the SV calling pipeline should also consider the running time and memory usage. The author seems to have forgotten this. Additionally, there are some typo errors in the manuscript. For example, in the Abstract, the "minimap2-cutsv" should be "minimap2-cuteSV", and so on.

**Response**: Thank you for bringing up this question. In the revision, we have added reports for the runtime and memory usage for all alignment and SV calling tools. We appreciate your pointing out the "typo errors," and we have made the necessary corrections.

In large-scale tasks such as analyzing SV samples from populations, how much resources pipelines use is crucial for getting the job done. We evaluated the runtime and memory consumption of aligners and callers based on 5x sequencing depth HG002 samples. Among the three sequencing technologies, minimap demonstrated the fastest speed (CCS: 10 min; CLR, ONT: ~15 min), while ngmlr was the slowest (CCS: 400 min, CLR: 80 min, ONT: 90 min) (**Figure S7 a**). Our findings align with the trend observed in Jingwen Ren's results, where the same aligner typically demonstrates a sequence of CLR>ONT>CCS in terms of runtime across different data types, such as CCS, CLR, and ONT. Considering our results along with read length and our experience during evaluation, it's clear that read length also affects aligner runtime, particularly ngmlr(**Figure S7 a,e**). In memory consumption, lra performed the best (CCS: 40 GB, CLR: 27 GB, ONT: 25 GB), while Winnowmap consumed the most memory in CCS at 110 GB (**Figure S7 b**). For callers, Sniffles2 is the fastest and consumes the least memory compared to Sniffles, making it ideal for large-scale SV analysis. Additionally, cuteSV(1.6min), cuteSV2(1.5min), and DeBreak(3min) also perform well in speed(**Figure S7 c**). Among the callers, Sniffles2, SVIM, Sniffles, DeBreak, Picky, SVision, cuteSV, and cuteSV2 consume less than 3.5G of memory, while pbsv consumes the most memory (30G) (**Figure S7 d**).

**Supplementary  Fig7 a-d**. The running time and memory usage of aligners and callers in pipelines. **a**. The running time of aligners; **b**. The memory usage of aligners; **c**. The running time of callers; **d**. The memory usage of callers.

**References**

1.	Jiang T, Liu Y, Jiang Y, Li J, Gao Y, Cui Z, Liu Y, Liu B, Wang Y: **Long-read-based human genomic structural variation detection with cuteSV.** *Genome Biol* 2020, **21:**189.

2.	Bolognini D, Sanders A, Korbel JO, Magi A, Benes V, Rausch T: **VISOR: a versatile haplotype-aware structural variant simulator for short- and long-read sequencing.** *Bioinformatics* 2020, **36:**1267-1269.

3.	Kirsche M, Prabhu G, Sherman R, Ni B, Battle A, Aganezov S, Schatz MC: **Jasmine and Iris: population-scale structural variant comparison and analysis.** *Nat Methods* 2023, **20:**408-417.

4.	Dierckxsens N, Li T, Vermeesch JR, Xie Z: **A benchmark of structural variation detection by long reads through a realistic simulated model.** *Genome Biol* 2021, **22:**342.

5.	Shafin K, Pesout T, Chang PC, Nattestad M, Kolesnikov A, Goel S, Baid G, Kolmogorov M, Eizenga JM, Miga KH, et al: **Haplotype-aware variant calling with PEPPER-Margin-DeepVariant enables high accuracy in nanopore long-reads.** *Nat Methods* 2021, **18:**1322-1332.

**2^nd^ round**

**Reviewer 1**

The authors have provided satisfactory answers to some of my previous comments. In particular, I appreciated the inclusion of public real SV set benchmarks, the inclusion of HiFi data, the details given on SV simulations, the use of recall and precision metrics. However, I still have major issues on other important points, such as the use of intersecting multiple pipelines (IMP) as ground truth SV sets, the lack of clarity in the method and result sections, the issue with SV typing as DUP and INS and their evaluation, and I find that the writing still need to be improved.

Major comments :

- I maintain that using the results of pipelines, ie. intersecting multiple pipelines (IMP), even if the stringency of intersection is somehow controlled, to evaluate the same pipelines is not a good way of assessing the quality of predictions. It only allows us to assess the extent to which a given pipeline gives similar results to other pipelines. Therefore, I would not trust these results. Not all "real" results are obtained with this methodology (since public benchmark SV call sets have been added in the paper) but the problem is that all "real" results are grouped together and the reader can not make the difference between results based on curated ground truth datasets obtained with other data and the ones obtained with the IMP method. Moreover, this is not clearly enough stated at the beginning of the result section which sample-SV type is assessed with which method.

- The description of SV datasets, either in the results or method sections, is still not clear enough. I suggest the authors that they more clearly distinguish the acquisition of sequencing data from the ground truth SV call sets (the use of the word "benchmark dataset" is ambiguous: it can refer to the sequencing data or the ground truth SV call set). How the IMP call set is obtained still lacks some clarity, for instance P. 11 l.1 which pipelines were used for the intersection is not indicated, one sentence describes the merging criteria in terms of distance, but the parameter of number of supporting SV callers is not explained and the chosen values are not justified.

- DUP and INS. If I understand correctly the answer of the authors, to avoid the issues that some inserted duplications are either reported as INS or as DUP by different callers (and in the ground truth SV set), they have created an additionnal category of DUP_INS that sums both types. This could work if the same insertion event is always represented with the same breakpoint positions as an INS or DUP type in the different compared vcf files. Have the authors checked this is the case ? A second problem, is that DUP and INS categories seem not to have been modified in the paper and the great majority of figures and numbers given in the paper present results only for the initial DUP and INS categories, that are likely to over-estimate FN and FP rates for some callers due to differences in reporting these variants.

- Concerning the evaluation of INS : it seems that they are evaluated as TP or FP with the same breakpoint distance criterium as other SV types, without comparing the inserted sequences. However, two large insertions located at the same position but with different inserted sequences should not be considered as identical events.

- BND (or TRA) have been included in the benchmark results, but most figures in Supplementary do not include this type of SV.

- Spearman correlation of pipelines. P. 5 l. 6 I do not agree with the statement that "pipelines perform similarly in simulated and real data" since we can find in Sup Fig 3 correlation coefficient values very low, such as O.3 for INS or 0.59 for DEL between SIM and HG002.

- A large part of the results concerns the strategy of combining the results of two or three pipelines, but how the results are combined is not clearly explained. Taking the union or the intersection of call sets leads to opposite results in terms of accuracy and recall. In particular, p. 8, at the beginning of this section in results, the authors should indicate if the combination is the union or the intersection of two sets, and when there are 3 sets, what is precisely the criterium to select SV calls. The authors should also indicate here that they did not test combinations of pipelines with different mappers. Moreover, the interpretations of these results are contradictory between the Result and Discussion sections : in the Results, the authors says it improves the accuracy of results "without significant improvement in recall" (p.8 l.34), whereas in the Discussion it is the reverse it "boosts recall" (p. 14 l. 38).

- The discussion section could be improved.

- Since the added value of this benchmark work, as stated by the authors, is to evaluate all combinations of mappers and callers, I would have expected the authors to discuss which one of the two steps of the pipeline have the most impact on the results.

- I do not agree with some statements of the Discussion :

- p. 14 l. 9 : "the performance of different pipelines is generally consistent in both simulated and real datasets". Figure S3 shows many poor correlation coefficients between performances on simulated data and real data (see previous comment)

- p. 14 l.34 : "This is especially true when combining pipelines utilizing the same aligner" : the experiments performed in this paper can not validate this statement since the authors did not test any combinations with different aligners.

- the last paragraph of discussion should be updated since the reference SV set is no longer totally obtained with the IMP method. I do not understand the last sentence which seems to refer to the multiple pipeline strategy for SV detection, while the beginning of the paragraph refer to SV pipeline evaluation.

- The writing of the manuscript still needs to be improved, as I noticed sentences that are grammatically incorrect, incorrect citations, typos, or undefined abbreviations, ... for instance :

several places : in DEL, in INS -> for DEL variants

p. 3 l. 8 : ref 24 Duan et al, is a benchmark focused on genotyping, which is a different problem than the discovery problem

p. 3 l. 8 : Lorenzo Tattini is not the first author of reference 22

p.3 l. 13 : Sunichi Kosugi should be followed by "et al", his -> their, and the reference number is incorrect (not ref 26)

p. 3 l. 20-22 : GIAB and HGSV have published papers on their benchmark data and they should be cited here.

p. 4 l. 22 : "TRA" is not defined and it is not consistently used in the paper with respect to the BND term

p. 4 l. 24 : grammatical problem with "generate creating"

p. 4 l. 25 : the sentence beginning by "Two platforms" has no verb

p. 5 l.25 : by caller -> by callers

p. 5 l. 28 : DUP_INS is not defined

p. 6 l. 2 : "fastest speed" -> largest speed

p. 6 l. 4 Jingwen Ren's results : misses a reference to a paper

p.6 l. 24 : Figure 10e -> Figure S8e

p.7 l. 5,6,7 and 9 : "support reads" -> "support(ing) read number"

p.7 l. 17 : genotypic -> genotype

p. 8 l. 5 : recall what means MIER at the beginning of the paragraph

p. 8 l.27 : merge -> merging

p. 9 l. 9 : remove "to"

p.9 l.13 : I do not understand the sentence "For SV types, ..."

p. 9 l. 16 : "In INV, Three-pipelines and Three-pipelines"

p. 11 l. 18 : "Call SV set" -> "SV call set"

**Reviewer 2**

All questions are well answered in full and detail

**Authors’ response**

The authors have provided satisfactory answers to some of my previous comments. In particular, I appreciated the inclusion of public real SV set benchmarks, the inclusion of HiFi data, the details given on SV simulations, the use of recall and precision metrics. However, I still have major issues on other important points, such as the use of intersecting multiple pipelines (IMP) as ground truth SV sets, the lack of clarity in the method and result sections, the issue with SV typing as DUP and INS and their evaluation, and I find that the writing still need to be improved.

Major comments :

1. I maintain that using the results of pipelines, ie. intersecting multiple pipelines (IMP), even if the stringency of intersection is somehow controlled, to evaluate the same pipelines is not a good way of assessing the quality of predictions. It only allows us to assess the extent to which a given pipeline gives similar results to other pipelines. Therefore, I would not trust these results. Not all "real" results are obtained with this methodology (since public benchmark SV call sets have been added in the paper) but the problem is that all "real" results are grouped together and the reader can not make the difference between results based on curated ground truth datasets obtained with other data and the ones obtained with the IMP method. Moreover, this is not clearly enough stated at the beginning of the result section which sample-SV type is assessed with which method.

Response: We thank the reviewer for this critical comment! Yes, the IMP benchmark may lead to bias. In the revised manuscript, we have excluded IMP's structural variation (SV) baseline and its associated evaluation results, retaining only real sample SV benchmark sourced from public databases (HG002, CHM13, NA12878, HG00512, HG00096). Additionally, we have clarified which dataset is used for evaluating different types of SVs in the samples (The text in blue with a grey background below; The 10 line of the second paragraph in the Study Design Review section under the Results section of the manuscript.). After removing the IMP-related results, we observed minimal minor changes in the overall results. For example, in the revised manuscript, the frequency of NGMLR in the top 10 combined pipelines exceeded that of pbmm2, becoming the most frequent aligner.

Due to the limited availability of real ground-truth SV datasets, pipelines were benchmarked only against insertion and deletion discovery in samples HG002 (GIAB[29]), CHM13 (HPRC[30]), HG00096, HG00512, and NA12878 (HGSV[31]). However, no benchmarking was conducted for duplications, inversions, or translocations for real samples[12].

Figure1. Performance of SV detection pipelines in different SV types (CCS). Precision and recall of DEL, DUP, INS, INV and BND were determined with the simulated (a, b(DUP_INS)) and the real data(c). F1-measures, which combine precision and recall statistics (see the "Methods" section for details), are depicted for pipelines distinguished by different coloured bars. Pipelines are categorized according to the alignment tools (lra, minimap2, ngmlr, pbmm2, winnowmap).

2. The description of SV datasets, either in the results or method sections, is still not clear enough. I suggest the authors that they more clearly distinguish the acquisition of sequencing data from the ground truth SV call sets (the use of the word "benchmark dataset" is ambiguous: it can refer to the sequencing data or the ground truth SV call set). How the IMP call set is obtained still lacks some clarity, for instance P. 11 l.1 which pipelines were used for the intersection is not indicated, one sentence describes the merging criteria in terms of distance, but the parameter of number of supporting SV callers is not explained and the chosen values are not justified.

Response: We thank the reviewer for this comment and suggestion! We describe the acquisition of sequencing data and the corresponding ground truth SV call set in two paragraphs. In the manuscript, we have revised the descriptions of the results and methods sections regarding the SV dataset to make them clearer. Firstly, in our manuscript, whenever we mention "benchmark," it refers to the ground truth SV call set. Secondly, we remove the evaluation results of the SV benchmark based on the MIP method. Finally, we have segmented the description of how reads and the benchmark SV call set were obtained in the methods section. Additionally, to provide a clearer presentation of the sources of reads and the benchmark SV call set, we have utilized tables (Table S3 in the revised manuscript).

Table S3:

sampleDELDUPINSINVBND

SIM72142919998951368

CHM137622-12448--

HG000966151-10091--

HG005126135-10118--

NA128786115-9956--

The evaluation was conducted using benchmark SVs from both simulated and real datasets. The simulated dataset comprised 20,541 non-overlapping SVs, including 7,214 deletions, 9,989 insertions, and 51 inversions identified within the CHM1 sample call set (dbVAR database accession nstd137[40], which includes INS sequence). Additionally, 2,919 duplications and 368 translocations extracted from the KWS1 sample call set (dbVAR database accession nstd106[41]) were included. The length distributions of deletions and insertions are similar, characterized by two main peaks at 50 bp and 300 bp. Duplication lengths are primarily concentrated in the ranges of 50 bp and 3,000 bp, while inversions exhibit peaks at 360 bp and 10 kb (Figure S1). All simulated translocation lengths are 10 kb[11]. The distribution of simulated SVs across the genome is generally consistent with that observed in real data (Figure S2). Due to the limited availability of real ground-truth SV datasets, pipelines were benchmarked only against insertion and deletion discovery in samples HG002 (GIAB[29]), CHM13 (HPRC[30]), HG00096, HG00512, and NA12878 (HGSV[31]). However, no benchmarking was conducted for duplications, inversions, or translocations for real samples[12]. As the SVs in HG002 are based on GRCh37, we utilized LiftOver to convert them to the GRCh38 version. Following conversion, we obtained 5,418 deletions and 7,266 insertions. For CHM13, there are 7,622 deletions and 12,448 insertions. In HG00096, there are 6,151 deletions and 10,091 insertions. HG00512 exhibits 6,135 deletions and 10,118 insertions, while NA12878 displays 6,115 deletions and 9,956 insertions (Table S3).

Reads utilized for SV detection pipelines were sourced from both simulated and real datasets. The VISOR (v1.1) [42] tool was employed to generate simulation reads (PacBio: CCS, CLR; Nanopore: R9.4, R10.4) using the human reference genome (version: GRCh38). Simulated reads from two platforms, PacBio and Nanopore, had mean read lengths of 8 kb, with default error models. Real read datasets for PacBio CCS included samples from CHM13, HG00096, HG002, HG003, HG004, HG00512, HG005, HG006, HG007, and NA12878, with average read lengths ranging from 10 kb to 18 kb (Table S2). The CLR datasets encompassed samples from CHM13, HG002, HG003, HG004, HG00512, HG005, HG006, and HG007, with average read lengths ranging from 8 kb to 25 kb. Nanopore datasets included samples from CHM13, HG00096, HG002, HG003, HG004, HG00512, and NA12878, with average read lengths ranging from 8 kb to 56 kb (Table S2). Additionally, real family data samples were utilized for assessment: Nanopore (HG002 (son), HG003 (father), HG004 (mother)), PacBio (HG002 (son), HG003, HG004), and (HG005 (son), HG006 (father), HG007 (mother)).

3. DUP and INS. If I understand correctly the answer of the authors, to avoid the issues that some inserted duplications are either reported as INS or as DUP by different callers (and in the ground truth SV set), they have created an additionnal category of DUP_INS that sums both types. This could work if the same insertion event is always represented with the same breakpoint positions as an INS or DUP type in the different compared vcf files. Have the authors checked this is the case ? A second problem, is that DUP and INS categories seem not to have been modified in the paper and the great majority of figures and numbers given in the paper present results only for the initial DUP and INS categories, that are likely to over-estimate FN and FP rates for some callers due to differences in reporting these variants.

Response: We appreciate the reviewer for this comment! We investigated instances where the caller erroneously classified insertions as duplications or vice versa, as illustrated in the table below. Furthermore, we analyzed the proportion of misclassifications across different data types and pipelines in simulated data. Regarding the second issue, both in the main figure and the manuscript, we maintained the results for insertions (INS) and duplications (DUP) while also integrating the results for events categorized as DUP_INS. Additionally, we observed a high consistency in the F1 measure levels of pipelines between DUP_INS and non- DUP_INS scenarios (Spearman correlation coefficient R= 0.82, Pearson correlation coefficient R= 0.95, Regression R-squared= 0.9, as shown in Figure S5). Our definition of an insertion is based on SV records with 'SVTYPE=INS', while a duplication is defined as SV records as long as it contains the 'DUP' character in the 'SVTYPE' field, e.g., 'SVTYPE=DUP', 'SVTYPE=DUP/INS' or 'SVTYPE=INVDUP'. Although different callers may classify duplication events differently, such as "INS/DUP" and "INVDUP", they all fall under the duplication category. Therefore, our evaluation of duplications will not lead to overestimating false negatives (FN) and false positives (FP) rates. The higher FN and FP levels in callers are more likely due to misclassifications of duplication categories. For instance, certain duplication events may exhibit insertion signal types in the alignment BAM files. However, most tools do not proceed with further similarity calculations between insertion signal sequences and genomic sequences for refined category determination.

Moreover, if SV records from different callers have 'DUP' in the SVTYPE field, they are considered duplications. On the other hand, because some callers might classify DUP as INS, we merge DUP and INS as INS for evaluation, labelled as DUP_INS. Our filtering criteria retained only variants marked as 'PASS' in the 'FILTER' field (In SVision, the 'FILTER' field does not use 'PASS'; we choose 'Covered' as the 'PASS' record.)

Furthermore, we conducted an in-depth analysis of INS and DUP types using simulation, as some tools do not distinguish between them. Initially, we observed that a significant fraction of DUP events were incorrectly identified as INS events by callers (30~60%), whereas only a minority of INS events were erroneously reported as DUP events by callers (0.153%) (Table S4). Moreover, minimap2 was found to exacerbate the proportion of DUP events reported as INS by callers (~70%) compared to other aligners. Conversely, NGMLR increased the proportion of INS events reported as DUP by callers compared to other aligners (~10%). To address this discrepancy, we re- evaluated them using a merged DUP_INS type in simulated data (i.e., transforming all duplications and insertions into insertions for evaluation). After excluding the aligner LRA due to its poor performance, we observed a high consistency in the F1 measure levels of pipelines between DUP_INS and non-DUP_INS scenarios (Spearman correlation R= 0.82, Pearson correlation R= 0.9, R^2= 0.95 Figure S5). Among the top 10 pipelines based on the F1 measure, eight were consistent for DUP_INS and non-DUP_INS scenarios (Figure 1). This high consistency suggests that the relative performance of most pipelines may not be significantly affected by the misclassification of DUP and INS events.

The caller misclassifies INS as DUP in simulated data (Note: this table is not included in the manuscript)：

chr1026940117Sim-INS.121END=26940118;SVTYPE=INS;SVLEN=4727

chr1026935700CCS-25x-DUP-lra-

cutesv2.27END=26940379;SVTYPE=DUP;SVLEN=4679

chr1186171245Sim-INS.5579END=86171246;SVTYPE=INS;SVLEN=4253

chr1186169371CCS-25x-DUP-lra-

cutesv2.40END=86173661;SVTYPE=DUP;SVLEN=4290

chr1927725359Sim-INS.2119END=27725360;SVTYPE=INS;SVLEN=4628

chr1927724068CCS-25x-DUP-lra-

cutesv2.93END=27728698;SVTYPE=DUP;SVLEN=4630

chr1927728623Sim-INS.7120END=27728624;SVTYPE=INS;SVLEN=4628

chr1927724068CCS-25x-DUP-lra-

cutesv2.93END=27728698;SVTYPE=DUP;SVLEN=4630

chr199019754Sim-INS.2240END=9019755;SVTYPE=INS;SVLEN=3161

chr199018097CCS-25x-DUP-lra-

cutesv2.92END=9021257;SVTYPE=DUP;SVLEN=3160

chr22295538Sim-INS.7725END=2295539;SVTYPE=INS;SVLEN=130

chr22295539CCS-25x-DUP-lra-cutesv2.95END=2295665;SVTYPE=DUP;SVLEN=126

The caller misclassifies DUP as INS in simulated data (Note: this table is not included in the manuscript)：

chr15214524Sim-DUP.1836END=5214791;SVTYPE=DUP;SVLEN=267

chr15214734CCS-25x-INS-lra-cutesv2.115END=5214734;SVTYPE=INS;SVLEN=264

chr18344791Sim-DUP.513END=8344891;SVTYPE=DUP;SVLEN=100

chr18344869CCS-25x-INS-lra-cutesv2.149END=8344869;SVTYPE=INS;SVLEN=95

chr1206924371Sim-DUP.1653END=206924624;SVTYPE=DUP;SVLEN=253

chr1206924372CCS-25x-INS-lra-

cutesv2.822END=206924372;SVTYPE=INS;SVLEN=253

chr1108453178Sim-DUP.71END=108458978;SVTYPE=DUP;SVLEN=5800

chr1108453793CCS-25x-INS-lra-

cutesv2.552END=108453793;SVTYPE=INS;SVLEN=5804

chr1161061314Sim-DUP.143END=161061436;SVTYPE=DUP;SVLEN=122

chr1161061407CCS-25x-INS-lra-

cutesv2.670END=161061407;SVTYPE=INS;SVLEN=122

chr128410088Sim-DUP.1724END=28410213;SVTYPE=DUP;SVLEN=125

chr128410089CCS-25x-INS-lra-

cutesv2.278END=28410089;SVTYPE=INS;SVLEN=114

Figure1. Performance of SV detection pipelines in different SV types (CCS). Precision and recall of DEL, DUP, INS, INV and BND were determined with the simulated (a, b(DUP_INS)) and the real data(c). F1-measures, which combine precision and recall statistics (see the "Methods" section for details), are depicted for pipelines distinguished by different coloured bars. Pipelines are categorized according to the alignment tools (lra, minimap2, ngmlr, pbmm2, winnowmap).

Figure S5. In simulated data, the correlation coefficient of F1-measure between pipelines using DUP_INS and non-DUP_INS. R1 represents the Pearson correlation coefficient, R2 represents the Spearman correlation coefficient, and R3 is the square of the Pearson correlation coefficient. The "INS.DUP" F1-measure represents the cumulative F1 scores of DEL and INS. The DUP_INS F1-measure represents the F1 scores of DUP_INS.

4. Concerning the evaluation of INS: it seems that they are evaluated as TP or FP with the same breakpoint distance criterium as other SV types, without comparing the inserted sequences. However, two large insertions located at the same position but with different inserted sequences should not be considered as identical events.

Response: We thank the reviewer for this comment and suggestion! We have updated the INS evaluation to incorporate considerations for the consistency of inserted sequences. Additionally, we have introduced a consistency gradient with similarity levels ranging from 0.9 to 0.3. However, during the detection of insertions, deviations in position and length, coupled with uncertainty regarding whether the INS sequences provided by the caller originate from randomly selected read signals or consensus sequences in multiple sequence alignments, can influence INS assessment based on sequence similarity. Thus, when the similarity level between two SV sequences at the same position falls below a certain threshold, it does not necessarily imply that they are truly different. Given these complexities, we have maintained the previous evaluation results while supplementing them with INS assessments under higher sequence similarity levels. This approach ensures a more comprehensive evaluation while acknowledging the nuances that can affect SV assessment.

Due to breakpoint deviations, length discrepancies, and the lack of INS sequences from some callers, most researchers do not consider sequence differences when evaluating pipeline performance on INS. Therefore, we initially assessed pipeline performance without considering INS sequence consistency, as shown in Figure S6-7.

We then analyzed performance changes under varying levels of INS sequence consistency. Our findings indicated that in simulated data, pipelines exhibited smaller declines in F1 scores when INS sequence consistency ranged from 0.3 to 0.5 compared to 0.6 to 0.9 (Figure S7a). A similar performance decrease pattern was observed in real data. The decline in F1 scores was smaller when INS sequence consistency ranged from 0.3 to 0.5 compared to 0.6 to 0.9 (Figure S7b). This suggests that while most pipelines generally detect INS positions accurately, they often do not achieve high sequence consistency.

Figure S6. The F1 score of insertions considering sequence consistency (0.7; CCS) in simulated and real sample data. The y-axis F1-measure reflects the cumulative INS F1 score across different samples.

SIM:

Figure S7. The distribution of F1 scores for samples SIM and HG002 under different insertions considering sequence consistency(0.3, 0.4, 0.5, 0.6, 0.8, 0.9).

5. BND (or TRA) have been included in the benchmark results, but most figures in Supplementary do not include this type of SV.

Response: We apologize for the confusion here! We have expanded the presentation of additional results concerning BND (or TRA) in the supplementary figures. However, as mentioned above, we have removed the results related to the IMP SV benchmark in this version, so only the simulated data section addresses BND (or TRA).

Figure S1. The SV length distribution in benchmark datasets includes both simulated (Sim).

Figure S12. F1 Distribution of pipelines in Pacbio and Nanopore sample data under 25x sequencing depth. e: BND.

Figure S8. Performance of SV detection pipelines in different SV types(CSS). Precision and recall of DEL, DUP, INS, INV and BND were determined with the simulated (a(precision), c(recall)) and the real data(b(precision), d(recall)). Precision-measure and recall-measure are shown for the pipelines indicated with orange (for DEL), yellow (for INS), green (for DUP), brown (for INV) and olive (for BND)bars. Pipelines are categorized according to the alignment tools (lra, minimap2, ngmlr, pbmm2, winnowmap).

6. Spearman correlation of pipelines. P. 5 l. 6 I do not agree with the statement that "pipelines perform similarly in simulated and real data" since we can find in Sup Fig 3 correlation coefficient values very low, such as O.3 for INS or 0.59 for DEL between SIM and HG002.

Response: We apologize for the confusion here! Indeed, the statement "pipelines perform similarly in simulated and real data" is not precise. We have rephrased it in the revised manuscript.

First, the relative performance of different pipelines shows moderate consistency across most simulated and real datasets. For example, the Spearman correlation of F1 values of pipelines' performance ranged from 0.4 to 0.7 for most SV types across different sequencing platforms (Figure S3-4).

Figure S4. Pipelines on simulation (SIM) and real data (HG002, HG00512, HG00096, CHM13, NA12878) for SV detection performance F1 in inter-sample "spearman"correlation.

7. A large part of the results concerns the strategy of combining the results of two or three pipelines, but how the results are combined is not clearly explained. Taking the union or the intersection of call sets leads to opposite results in terms of accuracy and recall. In particular, p. 8, at the beginning of this section in results, the authors should indicate if the combination is the union or the intersection of two sets, and when there are 3 sets, what is precisely the criterium to select SV calls. The authors should also indicate here that they did not test combinations of pipelines with different mappers. Moreover, the interpretations of these results are contradictory between the Result and Discussion sections : in the Results, the authors says it improves the accuracy of results "without significant improvement in recall" (p.8 l.34), whereas in the Discussion it is the reverse it "boosts recall" (p. 14 l. 38).

Response: We thank the reviewer for this comment and suggestion! In the methods section, we explained how to merge the results of multiple pipelines. We employed both intersection and union strategies and clarified the criteria for accurately selecting SV calls when there are three sets. The results and related discussion sections concerning merging multiple pipelines have been rewritten.

Figure 6. The significance of F1, recall, and precision for different SV types in the top 10 combined pipelines compared to the individual pipelines that constitute the combination. a. Overall improvement levels of DEL and INS in the top 10 combined pipelines across different combination methods in both simulated and real datasets. b. Improvement levels of different SV types in the top 10 combined pipelines across various combination methods in simulated and real datasets. 'Caller' represents combined pipelines based on the same aligner with different callers. In contrast, 'Aligner' represents combined pipelines based on the same caller with different aligners. The table header format, such as "2U, 2I, 3U...", indicates the combination method: the first number represents the number of combined pipelines, and the second character indicates whether the combination is based on union (U) or intersection (I). On the left side of the table, 'REAL' denotes real data, and 'SIM' denotes simulated data. The arrows in the table represent whether the performance of the combined pipeline increased or decreased compared to the individual pipelines that constitute the combination. The integer following the arrow indicates the number of combined pipelines with and without significance after merging in the top 10 combinations. The final percentage represents the extent of the performance improvement of the combined pipeline compared to the individual pipelines. Lastly, white shading represents combinations with no significance, orange indicates a significant improvement in performance, and green denotes a significant decrease in performance.

Some studies employ a combination of multiple pipelines or algorithms to enhance the detection accuracy of SV calling. However, the optimal strategy for merging multiple pipelines based on TGS data has not been systematically investigated. To address this gap, we calculated the accuracy, recall, and median F1 scores of merged pipelines consisting of two, three, and four individual pipelines, employing different merge strategies for various SV types (DEL, INS, INV, DUP; Figure 6). Specifically, to assess the influence of aligners and callers on the merged results, we categorized our pipeline combinations into two groups: pipelines with the same aligner but different callers (the caller-based combination group) and pipelines with the same caller but different aligners (the aligner-based combination group). Moreover, considering that the choice of merge strategy (intersection or union) can significantly affect the results, we employed 'minimum number of supporting caller' values of 1 (union) and 2 (intersection) when using SURVIVOR to combine the results of two or more pipelines. Consequently, the combination categories were designated as <caller/aligner><pipelines number><union/intersection>, for example, caller_2U, caller_2I, aligner_3U, etc. Due to the large number of possible combinations resulting from the 41 pipelines (see Methods), we ranked them based on the median F1 score of the combined pipelines and focused our analysis on the top 10 combinations. The performance enhancement achieved by combined pipelines was more pronounced in simulated data than in real data, although pipeline combinations consistently improved performance (Figure 6, S23). For instance, in real data, the caller_2U combination improved precision by only 2.4%, with no significant changes in F1 score and recall before and after the pipeline merge. In contrast, in simulated data, there were moderate improvements across all metrics: F1 score (4.7%), recall (4.7%), and precision (4%) (Figure 6a). The enhancement of the caller- and aligner-based combination groups was very similar in simulated data (Figure 6, S23). However, in real data, these two groups exhibited slight differences. Specifically, the 3U and 4U combinations based on callers focused more on improving recall, while those based on aligners emphasized enhancing precision. Regarding the number of combined pipelines, incorporating more pipelines led to greater improvements. In addition, choosing the appropriate merging strategy (union or intersection) based on the number of pipelines being combined was crucial. When the number of pipelines was small, for instance, two, using the union strategy might result in more significant improvements than the intersection strategy. Conversely, if three or more pipelines were used, the intersection strategy led to more enhancement in performance (Figure 6). The analysis showed that pbmm2 had the highest frequency among the top ten combined pipelines regardless of the sequencing platforms (ONT: 0.451, CCS: 0.452, CLR: 0.455, Figure S24a). Following pbmm2, LRA (CCS: 0.153), minimap2 (CLR: 0.261), and winnowmap (ONT: 0.19) were the aligners with the second-highest frequencies in the ten combined pipelines. Additionally, we noticed that the choice of aligner in combined pipelines displayed specificity in detecting specific SV types. For instance, pbmm2 was more prevalent in the top 10 merged pipelines for DEL (0.5) and INS variants (0.78) (Figure S24c). Conversely, NGMLR (0.97) was the most common aligner in the top 10 merged pipelines for INV, while winnowmap (0.93) was frequently observed in pipelines targeting DUPs.

Finally, we compiled the frequency of variant callers among the top 10 merged pipelines. Among the combined pipelines (sorted by median F1 measure for DEL and INS), callers such as cuteSV (0.17-0.20), cuteSV2 (0.14-0.17), DeBreak (0.23-0.27), and Sniffles2 (0.07-0.16) exhibited higher frequencies (Figure S24b). Moreover, the distribution of callers varied among the top 10 merged pipelines for different SV types. Among the top 10 merged pipelines (ranked by median F1 score) for various SV types, cuteSV2 and SVIM were more frequently observed for detecting DEL (cuteSV2: 0.272, SVIM: 0.25). For INS variants detection, callers cuteSV, cuteSV2, and DeBreak had higher frequencies among the top 10 merged pipelines (cuteSV: 0.18, cuteSV2: 0.16, DeBreak: 0.17, Figure S24d). Additionally, callers SVIM (0.24) and SVision (0.23) displayed elevated frequencies for INV. In the case of DUP, callers cuteSV (0.18), cuteSV2 (0.19), and pbsv (0.18) exhibited higher frequencies in the top 10 merged pipelines (Figure S24d). Consequently, these callers with a high frequency among the top 10 merged pipelines should be given higher priority when considering pipeline combinations to enhance the performance of SV detection in TSG data.

8. The discussion section could be improved.

- Since the added value of this benchmark work, as stated by the authors, is to evaluate all combinations of mappers and callers, I would have expected the authors to discuss which one of the two steps of the pipeline have the most impact on the results.

- I do not agree with some statements of the Discussion :

- p. 14 l. 9 : "the performance of different pipelines is generally consistent in both simulated and real datasets". Figure S3 shows many poor correlation coefficients between performances on simulated data and real data (see previous comment)

Response: We apologize for the confusion here! We have revised this section in the latest version of the manuscript. The updated content is as follows:

First, the relative performance of different pipelines shows moderate consistency across most simulated and real datasets. For example, the Spearman correlation of F1 values of pipelines' performance ranged from 0.4 to 0.7 for most SV types across different sequencing platforms (Figure S3-4).

- p. 14 l.34 : "This is especially true when combining pipelines utilizing the same aligner": the experiments performed in this paper can not validate this statement since the authors did not test any combinations with different aligners.

Response: We apologize for the confusion here! In the revised manuscript, we have added multiple pipeline combination schemes based on callers (with aligner fixed) and aligners. Additionally, we have rewritten the related results and discussion sections. When analyzing the pipeline-combination results, we observed that both caller-based combinations (with aligner fixed) and aligner-based combinations can similarly improve performance.

- the last paragraph of discussion should be updated since the reference SV set is no longer totally obtained with the IMP method. I do not understand the last sentence which seems to refer to the multiple pipeline strategy for SV detection, while the beginning of the paragraph refer to SV pipeline evaluation.

Response: We apologize for the confusion here! We have rewritten the last two paragraphs of the discussion section. We also evaluated all multi-pipeline combinations based on aligners and callers and discussed the impact of aligners and callers on the results. Additionally, we have removed lines 9 and 34 from page 14 in the previous version of the manuscript.

When analyzing the pipeline-combination results, we observed that both caller-based combinations (with aligner fixed) and aligner-based combinations can similarly improve performance. However, we also noted that the performance variability due to callers was greater than that due to aligners among the 53 individual pipelines. This difference may stem from the greater variability among the twelve callers compared to the five aligners in the 53 pipelines. Thus, the choice of callers leads to greater performance variability in pipelines than the choice of aligners. Despite this, when merging results based on callers and aligners, we selected combinations from the top 10 performing pipelines, excluding those associated with weaker callers. This selection resulted in similar performance improvements when comparing the caller-based to the aligner- based combinations results. Nevertheless, considering computational storage and time, merging multiple pipelines based on different callers is more feasible. Additionally, when merging a small number of pipelines (e.g., two pipelines), using a union method might be more effective than an intersection method. Conversely, the intersection method is more reliable for merging a larger number of pipelines. This is likely because, when combining two pipelines using the union method, the number of correctly identified SVs shared between the two pipelines is greater than the number of incorrect SVs. However, as the number of combined pipelines increases, the growth rate of incorrect SVs exceeds that of correct SVs.

Our study has several areas that need improvement. First, due to the lack of benchmark datasets for DUP, INV, and TRA in real data, evaluating pipeline performance for these SV types only based on simulated data may introduce bias. For example, in real data, the detection performance for DEL and INS by the pipelines is approximately 10% lower than in simulated data. Additionally, the datasets used in our experiments might be limited in scale and representativeness. Due to resource constraints, we only utilized a few publicly available datasets, which might restrict the generalizability of our findings. Future research should incorporate larger and more diverse datasets to enhance the reliability and generalizability of the results.

9. The writing of the manuscript still needs to be improved, as I noticed sentences that are grammatically incorrect, incorrect citations, typos, or undefined abbreviations, ... for instance :

several places : in DEL, in INS -> for DEL variants

Response: We thank the reviewer for this comment and suggestion! We have noted the points and carefully revised the manuscript. We have revised instances of "in DEL, in INS" to "for DEL variants" throughout the manuscript. For example:

Our findings revealed that for DEL variant detection, the pipelines performed significantly better on CCS datasets compared to R9 and CLR (p=8.1e-8, p=8.9e-5, t- test) (Figure S12a).

Regarding precision, the top five pipelines for DEL variant detection in real samples are pbmm2-cuteSV, NGMLR-cuteSV, minimap2-cuteSV, LRA-cuteSV, and LRA- cuteSV2 (precision > 0.91) However, for some pipelines, the F1 scores initially increased and then decreased with an increase in minimal supporting reads for INS variants, owing to the balance between precision and recall.

p. 3 l. 8: ref 24 Duan et al, is a benchmark focused on genotyping, which is a different problem than the discovery problem

Response: Thanks for pointing out the problem! We have removed the citation to Duan et al. from the revised manuscript.

Despite previous evaluations bringing attention to SV pipeline calling, assessments based on TGS are still limited and lack comprehensiveness, indicating the need for further improvement and supplementation. For example, the evaluation of Anbo Zhou et al. provided interesting insights into the usage and performance of pipelines at an earlier stage. However, the aligners (e.g., GraphMap[1] and LAST[2]) they assessed are now less commonly used due to the evolution of tools and technologies[3]. Moreover, Davide Bolognini et al.'s pipeline evaluation [4] and the above studies did not consider the genotype accuracy in terms of Mendelian error rate (MIER). Due to the lack of precise reference data, the MIER assessment may offer a favourable method to evaluate pipelines' detection capabilities in depth. Additionally, most studies overlooked the length and breakpoint deviation of SVs detected by pipelines, which is crucial for SV functionality annotation and prediction. While Shunichi Kosugi et al. considered breakpoint and length deviation in their evaluation, the primary focus was on SGS calling algorithm performance rather than TGS[5]. Dierckxsens et al. developed Sim-it, a tool for simulating SVs and long reads, and evaluated the strengths and weaknesses of 7 callers and long-read sequencing platforms[6]. Their study introduced a new method called combiSV, which combines results from SV callers into a higher-quality call set with improved recall and precision. However, these evaluations mainly focused on callers and a few aligners, lacking analysis of the impact of aligners on SV detection. In addition to existing evaluation studies, available benchmark resources for SVs are gradually increasing, such as Genome in a Bottle (GIAB)[7], Human Genome Structural Variation Consortium (HGSV)[8], and The Human Pangenome Reference Consortium (HPRC)[9]. Although the studies and datasets mentioned above have provided many insights and assistance for better SV detection, a comprehensive evaluation of SV detection pipelines remains essential.

p. 3 l. 8: Lorenzo Tattini is not the first author of reference 22

Response: We have updated the author names to match the first author in the cited references.

Moreover, Davide Bolognini et al.'s pipeline evaluation [23] and the above studies did not consider the genotype accuracy in terms of Mendelian error rate (MIER). p.3 l. 13: Sunichi Kosugi should be followed by "et al", his -> their, and the reference number is incorrect (not ref 26)

Response: We have added "et al." and updated the citation format accordingly.

While Shunichi Kosugi et al. considered breakpoint and length deviation in their evaluation, the primary focus was on SGS calling algorithm performance rather than TGS[5].

p. 3 l. 20-22: GIAB and HGSV have published papers on their benchmark data and they should be cited here.

Response: We have added citations for GIAB and HGSV-related references. For example:

Due to the limited availability of real ground-truth SV datasets, pipelines were benchmarked only against insertion and deletion discovery in samples HG002 (GIAB[7]), CHM13 (HPRC[8]), HG00096, HG00512, and NA12878 (HGSV[9]).

p. 4 l. 22: "TRA" is not defined and it is not consistently used in the paper with respect to the BND term

Response: We have defined TRA and explained the relationship between TRA and BND in our evaluation.

Performance comparison was based on the F1 measure, aggregating F1 scores across different SV types (DEL, INS, INV, DUP, BND), as well as precision and recall measures for each SV type, ranging from 0 to 5. Currently, many callers report translocations (TRA) in the form of breakpoints (BND), lacking complete TRA information. Therefore, referring to the work of Tao Jiang et al.[11], we used the BND records in the VCF file, which might represent the TRA type.

p. 4 l. 24: grammatical problem with "generate creating"

Response: The content has been revised. The updated text is as follows: The VISOR (v1.1) [10] tool was employed to generate simulation reads (PacBio: CCS, CLR; Nanopore: R9.4, R10.4) using the human reference genome (version: GRCh38).

p. 4 l. 25: the sentence beginning by "Two platforms" has no verb

Response: The content has been revised. The updated text is as follows:

Simulated reads from two platforms, PacBio and Nanopore, had mean read lengths of 8 kb, with default error models.

p. 5 l.25: by caller -> by callers

Response: We have rewritten the relevant section due to the removal of results related to the IMP SV benchmark. This revision has helped avoid this type of error.

p. 5 l. 28 : DUP_INS is not defined

Response: We added an explanation for "DUP_INS" when it first appeared in the Results section. The specific content is as follows:

To address this discrepancy, we re-evaluated them using a merged DUP_INS type in simulated data (i.e., transforming all duplications and insertions into insertions for evaluation)

p. 6 l. 2: "fastest speed" -> largest speed

Response: We have revised "fastest speed" to "largest speed." The specific content is as follows:

Among the three sequencing technologies, minimap demonstrated the largest speed (CCS: 10 min; CLR, ONT: ~15 min) based on 5x sequencing depth, while NGMLR was the slowest (CCS: 400 min, CLR: 80 min, ONT: 90 min, Figure S11 a).

p. 6 l. 4: Jingwen Ren's results: misses a reference to a paper

Response: The relevant citations have been added to the latest version of the manuscript.

Our findings align with the trend observed in Jingwen Ren's results[15], where the same aligner typically demonstrates a sequence of CLR>ONT>CCS in terms of runtime across different data types.

p.6 l. 24: Figure 10e -> Figure S8e

Response: We have carefully reviewed and corrected similar errors in figure legends. We conducted a comparative analysis of Nanopore (R9, R10) and PacBio (CCS, CLR) data across multiple sample datasets using various pipelines (Figure S12). Our findings revealed that for DEL variant detection, the pipelines performed significantly better on CCS datasets compared to R9 and CLR (p=8.1e-8, p=8.9e-5, t-test) (Figure S12a). Similarly, the F1 score of pipelines for INS variants detection on real CCS datasets was significantly better than R9 and CLR (p=2.9e-11, p=7.6e-16, t-test) (Figure S12b). Additionally, while no significant differences were observed between the R9 and CLR datasets for DEL variants detection, significant differences were evident for INS variants detection (p=5.7e-9, t-test). However, no significant differences were observed in simulated data among the R9, R10, CLR, and CCS datasets for every SV type (Figure S12a-e).

p.7 l. 5,6,7 and 9 : "support reads" -> "support(ing) read number"

Response: Thanks! We have updated all instances of "support reads" to "minimal supporting reads."

p.7 l. 17: genotypic -> genotype

Response: We have revised the term "genotypic" to "genotype."

p. 8 l. 5: recall what means MIER at the beginning of the paragraph

Response: We have removed the initial mention of "MIER" in this section. Additionally, we have revised the title of this section to "Mendelian Error Rate Calculation" to aid in understanding the abbreviation MIER. Furthermore, the last three lines of this section: "Therefore, pipeline performance and genotype accuracy can be evaluated by analyzing Mendelian errors in SVs called among family members," also help to clarify the concept of MIER mentioned later.

p. 8 l.27: merge -> merging

Response: The subheading in the Results section was titled "Pipeline results merge to improve performance." This has now been revised to "Pipeline results merging to improve performance."

p. 9 l. 9: remove "to"

Response: We have completely revised the entire results section corresponding to the multi-pipeline merging.

p.9 l.13: I do not understand the sentence "For SV types, ..."

Response: We have completely revised the entire results section corresponding to the multi-pipeline merging.

p. 9 l. 16: "In INV, Three-pipelines and Three-pipelines"

Response: We have completely rewritten the results section corresponding to the multi-pipeline merging.

p. 11 l. 18 : "Call SV set" -> "SV call set"

Response: The "Call SV set filtering." has been revised to "SV call set filtering."

**3^rd^ round**

**Reviewer 2**

All questions are well answered in full and detail.
